# Synthesis, Characterization, and Study of Catalytic Activity of Chiral Cu(II) and Ni(II) Salen Complexes in the α-Amino Acid C-α Alkylation Reaction

**DOI:** 10.3390/molecules28031180

**Published:** 2023-01-25

**Authors:** Anna S. Tovmasyan, Anna F. Mkrtchyan, Hamlet N. Khachatryan, Mary V. Hayrapetyan, Robert M. Hakobyan, Artavazd S. Poghosyan, Avetis H. Tsaturyan, Ela V. Minasyan, Victor I. Maleev, Vladimir A. Larionov, Armen G. Ayvazyan, Norio Shibata, Giovanni N. Roviello, Ashot S. Saghyan

**Affiliations:** 1 Scientific and Production Center “Armbiotechnology” of NAS RA, 14 Gyurjyan Str., Yerevan 0056, Armenia; 2 Institute of Pharmacy, Yerevan State University, 1 Alex Manoogian Str., Yerevan 0025, Armenia; 3 Scientific and Technological Center of Organic and Pharmaceutical Chemistry of NAS RA, 26 Azatutyan Ave., Yerevan 0014, Armenia; 4 A.N. Nesmeyanov Institute of Organoelement Compounds of Russian Academy of Sciences, Vavilov Str. 28, 119991 Moscow, Russia; 5 Faculty of Science, Peoples’ Friendship University of Russia (RUDN University), Miklukho-Maklaya Str. 6, 117198 Moscow, Russia; 6 Department of Life Science and Applied Chemistry, Nagoya Institute of Technology, Showa-ku, Nagoya 466-8555, Japan; 7 Institute of Biostructures and Bioimaging, Italian National Council for Research (IBB-CNR), Area di Ricerca Site and Headquarters, Via Pietro Castellino 111, 80131 Naples, Italy

**Keywords:** phase transfer catalysis, asymmetric catalysis, salen complexes, alkylation, amino acids

## Abstract

A new family of Cu(II) and Ni(II) salen complexes was synthesized and fully characterized through various physicochemical methods. Their catalytic activity was evaluated in the phase transfer C_α_-alkylation reaction of the Schiff bases of D,L-alanine ester and benzaldehyde derivatives. It was found that the introduction of a chlorine atom into the *ortho*- and *para*-positions of the phenyl ring of the substrate resulted in an increase in both the chemical yield and the asymmetric induction (*ee* 66–98%). The highest enantiomeric excess was achieved in the case of a Cu(II) salen complex based on (*S*,*S*)-cyclohexanediamine and salicylaldehyde at −20 °C. The occurrence of a bulky substituent in the ligand present in the complexes led to a drastic decrease in *ee* and chemical yield. For instance, the introduction of bulky substituents at positions 3 and 5 of the phenyl ring of the catalyst resulted in a complete loss of the stereoselectivity control in the alkylation reaction.

## 1. Introduction

Chiral salen transition metal complexes have a significant role in the stereoselective synthesis being efficient chiral catalysts usable in a plethora of asymmetric transformations [1,2,3,4]. Their simple synthesis and the versatility in their structural modification have contributed to the current increased interest in the synthesis and characterization of new salen metal complexes [5,6,7,8,9,10]. From an applicative perspective, such complexes of the first-row transition metals are known for their attractive catalytic, magnetic and biological properties [11,12,13,14,15,16,17,18]. Due to their structural diversity and selectivity in the coordination with metal ions, Schiff bases have found wide application in the asymmetric catalysis as ligands. For example, dinuclear positively charged Cu(II) complexes of Schiff base derivatives were used as catalysts in Chan–Evans–Lam C-N coupling reactions [19].

**Figure 1:**
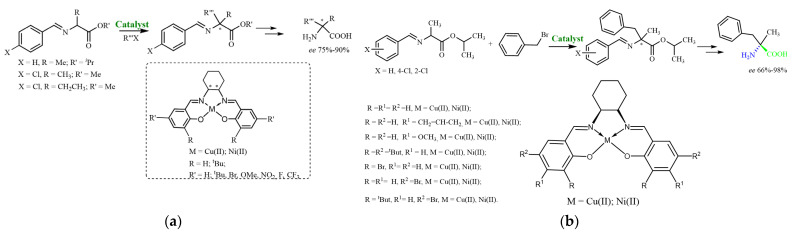
Asymmetric alkylation of amino acid enolates under PTC according to the previous scientific literature (**a**) and the current work (**b**).

The research group led by Klein reported studies devoted to the realization of an unprecedented high valent Ni^III^ bisphenoxyl diradical species [20]. Moreover, Ni(II) salen complexes were used as highly active Ni catalysts for the reductive amination of ketones by ammonia [21,22]. Chiral transition metal complexes were used to catalyze a wide range of asymmetric transformations, and North and Belokon’s group has shown that these complexes can be used to catalyze the asymmetric alkylation of amino acid enolates under phase transfer catalysis (PTC) [23,24,25,26]. Before that, most work in this area had focused on the use of quaternary ammonium salts as the phase transfer catalyst using cinchona alkaloid derivatives and C2-symmetric binaphthyl compounds [27,28,29]. In 1999, North and Belokon reported that Ni(II) salen complex (10 mol%) could catalyze the asymmetric benzylation of alanine enolate, leading to α-methyl phenylalanine with 30% enantiomeric excess [23,24]. The corresponding Cu(II) salen complex was found to be a far more effective catalyst, and 2 mol% of this complex was sufficient to catalyze the same reaction with 88% enantiomeric excess. Cu(II) salen complex also catalyzed the asymmetric alkylation of alanine enolate with other alkyl halides, affording substituted α-methyl α-amino acids with 75–90% enantiomeric excess [23,24] (Figure 1a).

Different chiral metal salen complexes were tested as catalysts for the PTC C_α_-alkylation of aldimine Schiff bases of alanine esters with benzyl bromide under phase transfer conditions [30]. It was shown that the structure of the imine in the substrate significantly affected the enantioselectivity of the asymmetric phase transfer of the alkylation reaction of the alanine methyl ester [25].

Due to the importance of the chiral salen complexes in the asymmetric catalysis, the rational design of such complexes for their evaluation as effective catalysts in various chemical reactions is clearly required to understand the implications of their structural characteristics on their activity, but also the role of their coordination ability and of their specific physicochemical properties required to develop new effective ligand systems. 

Thus, in this work, we report on the synthesis of Cu(II) and Ni(II) salen complexes that were studied and characterized by analytical, spectroscopic and other physicochemical methods. The catalytic activity and the stereoinduction ability of the obtained complexes were evaluated in the phase transfer alkylation of racemic amino acid precursors in order to obtain α,α-disubstituted amino acids. 

We attempted to determine the dependence of the asymmetric yield on the type of substituents present in the salicylidene fragment of the complexes that were used as catalysts. Considering the fact that *N*-benzylidenealanine isopropyl ester is not a practical substrate in the PTC reactions, we have obtained new derivatives of *N*-benzylidenealanine isopropyl ester—*N*-2-Cl-benzylidenealanine and *N*-4-Cl-benzylidenealanine isopropyl esters—which were tested as the initial amino acid substrates. It was found that the introduction of chlorine atoms at the *ortho*- and *para*-positions of the substrate led to increased chemical and asymmetric yields (*ee* up to 98.7%), as well as an increase in the stability of the substrate itself.

**Scheme 1:**
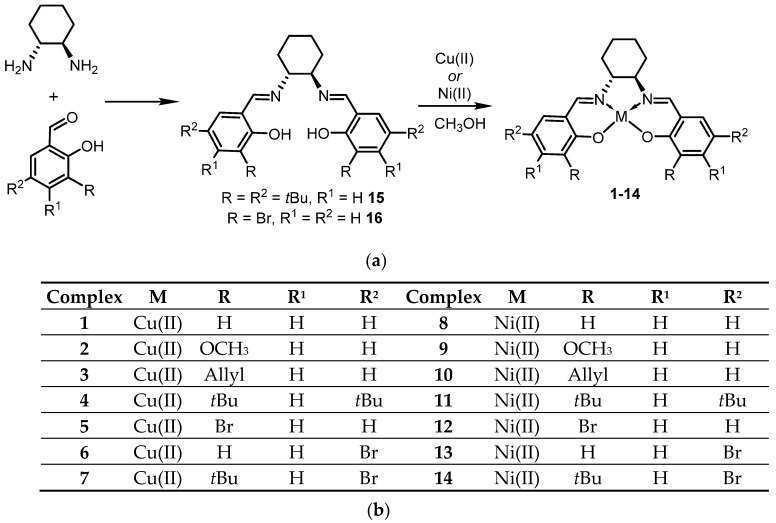
Synthetic procedure adopted by us for the synthesis of the Cu(II) and Ni(II) salen complexes described in our work (**a**). The identity of metal and substituents is indicated for all complexes (**1**–**14**) in part (**b**) of the Scheme 1.

It should be noted that **1**, **4**, **8**, **9** and **11** compounds were previously synthesized [20,23,24,25,26], but were never evaluated before as catalysts in the phase transfer alkylation with the *N*-2-Cl-benzylidenealanine and *N*-4-Cl-benzylidenealanine isopropyl esters (Scheme 1).

## 2. Results and Discussion

### 2.1. Synthesis of Salen Complexes

Chiral Cu(II) and Ni(II) salen complexes were synthesized as depicted in Scheme 1. The synthesis of all the metal complexes was mainly carried out under in situ conditions at room temperature under vigorous stirring. 

The condensation of (*S*,*S*)-cyclohexanediamine and salicylaldehyde derivatives in a 1:2 molar ratio followed by the complexation with metal ions furnished the desired complexes in good yields. Notably, in the case of complexes **4**, **6**, **11** and **13**, the synthesis was realized in two steps via formation of the corresponding Schiff bases **15** and **16**. As for their physical description, the resulting metal complexes were all air-stable brown solids. While complexes **3**, **5**–**7**, **9**, **10** and **12**–**14** were synthesized for the first time in the context of the current study, **1**, **2**, **4**, **8** and **11** had been synthesized previously [20,23,24,25,26].

### 2.2. Spectroscopic Studies (IR)

The FT-IR spectra of all synthesized complexes are shown in the Supplementary Materials. No peaks associable to aldehyde groups in the 1700–1800 cm^−1^ range were detected in the spectra, which suggested that all samples were free from any traces of initial aldehydes. A strong C=N stretching vibration was found in all spectra in the 1610–1620 cm^−1^ range, confirming the Schiff base formation in all cases. More interestingly, while C=N peaks are generally detected in the 1620–1640 cm^−1^ range, in our case all Schiff base peaks shifted to lower wavenumbers, indicating the formation of complexes with metals [31]. In the 400–600 cm^−1^ range new stretching vibration signals were detected, which corresponded to the coordinated bond between metal and nitrogen atoms. The intensity of all peaks corresponding to the Ni(II) complexes was lower than that found for the Cu(II) complexes, which suggests that salen ligands are binding Cu^2+^ ion more specifically and with higher affinity than Ni^2+^.

It should be noted that complexes **3**, **5**–**7**, **9**, **10** and **12**–**14** were studied spectrophotometrically for the first time in this work.

### 2.3. TG/MS Studies

**Figure 2:**
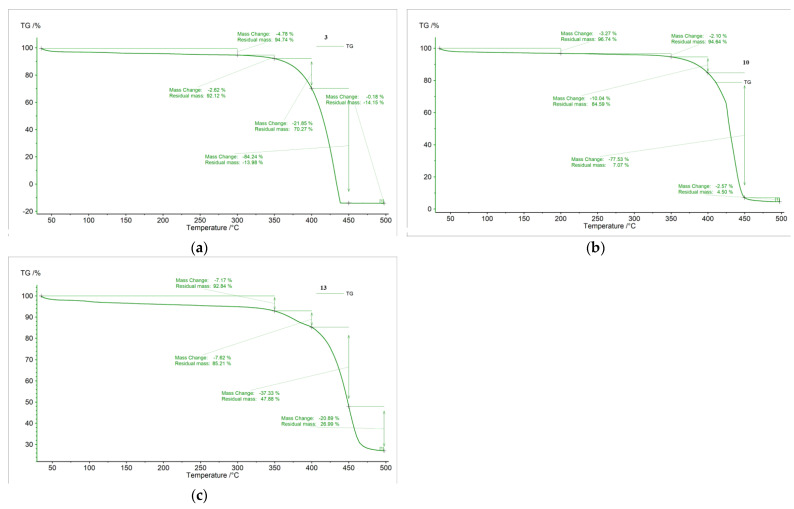
Thermogravimetric analysis of the complexes **3** (**a**), **10** (**b**) and **13** (**c**).

Complexes **3**, **10** and **13** were studied by thermogravimetric methods, in analyses during which the samples were heated in a helium medium with a temperature ramp of 10 °C/min from room temperature (25 °C) to 500 °C. As a result, the following graphics were obtained (Figure 2).

According to the obtained data (Figure 2), practically the compounds were stable up to 300 °C, with their decomposition mainly occurring in the 300–450 °C temperature range. The experiments performed on samples of the three complexes furnished different results (Figure 2a,b,c, respectively). The thermogravimetric data collected for these complexes after the above-mentioned analyses are given in Table 1.

During the study, we used a mass spectrometer, and the obtained data are presented in SI. Unfortunately, we were not able to trace a clear fragmentation. Anyway, according to the mass-spectrometric data the main fragments for all complexes during the destruction process coincided with the corresponding fragments of ligand residues. Because of the decomposition, it was not possible to isolate individual molecules, but it was possible to identify some of their fragments and ions that proved that the decomposition of the experimental samples took place. Furthermore, the structures of the obtained Ni(II) salen complexes were characterized by NMR as described in the experimental part.

### 2.4. X-ray Analysis

**Figure 3:**
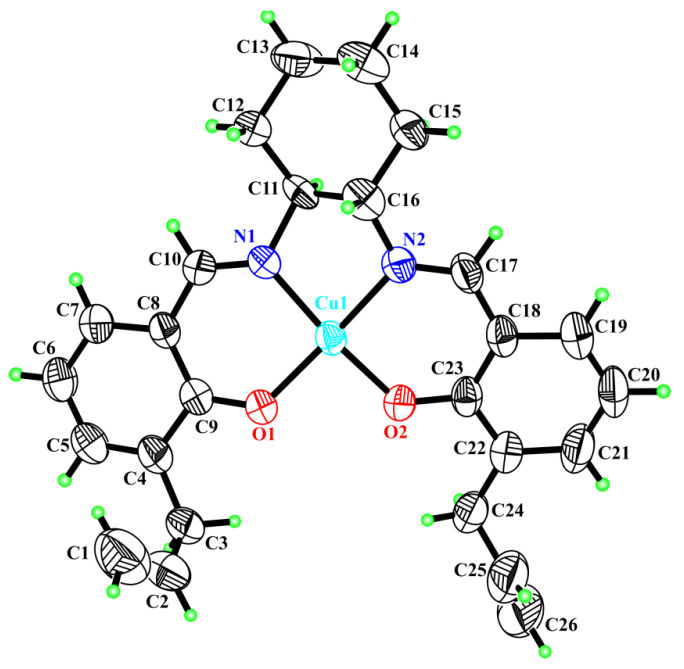
The X-ray structure for complex **3** with thermal displacement ellipsoids drawn at the 50% probability level (disordered atoms omitted for clarity).

We were able to grow a crystal from the racemic metal complex **3** that underwent X-ray analysis. We observed that the Cu atom in the complex was four-coordinated in a distorted planar geometry with a mean deviation of 0.0450(5) Å from the plane generated by the atoms of the Schiff base ligand (Figure 3).

The copper was linked to two oxygen and two nitrogen atoms; the bond lengths for Cu1-O1, Cu1-O2, Cu1-N1 and Cu1-N2 were 1.893(3) Å, 1.889(3) Å, 1.924(4) Å and 1.935(4) Å, respectively. The above-mentioned bond distances are typical for Cu(II) and were previously crystallographically characterized [32,33]. The analysis of ellipsoids of anisotropic thermal vibrations and difference electron density Fourier maps showed that atoms C11, C13, C14 and C16 of the cyclohexane cycle were statistically disordered between two positions. The final model was refined by splitting the atomic positions of the above-mentioned atoms, and the occupancy factors of these two positions were about 52% and 48%. Complex **3** was endowed with chirality with the asymmetric center on atoms C11 and C16 belonging to the cyclohexane ring. Therefore, the complex was crystallized in the centrosymmetric space group P-1 and chiral atoms were statistically disordered in two positions corresponding to the conformers (*S*,*S*) and (*R*,*R*). Thus, we can conclude that the crystal was a racemic mixture of (*S*,*S*) and (*R*,*R*) conformers in a ratio of 52% and 48%. In both statistically disordered positions, the cyclohexane ring showed a well-expressed “chair” conformation. Four atoms (C11, C12, C14A(B), C15A(B)) of the ring were in the same plane, the maximum deviation of atoms from the mean-squared plane did not exceed 0.0070(5)Å, and atoms C13A(B) and C16A(B) were out of plane with a shift from “chair” plane on 0.7183(5)Å (0.7425(5)Å) and 0.7621(5)Å (−0.7663(5)Å), respectively. In 3D packing, the intermolecular interactions could be described mainly via van der Waals forces.

### 2.5. DFT Analysis of Complex ***3***

#### Conformational Analysis

To this scope, we used GAMESS-US, a computational chemistry software that allows one to simulate a single crystal of the complex. Thus, we carried out a geometry optimization analysis with the DFT/B3LYP method under the 6-31G(d) basis set and CH_2_Cl_2_ PCM model for the synthesized complex **3**. All atom coordinates and 3D structures of optimized complexes are presented in the Supplementary Materials. The obtained simulation analysis results illustrated their similarity and high accuracy with X-ray crystallographic data of complex **3.** Bond lengths of O and N atoms linked with Cu atoms are presented in Table 2.

The obtained bond lengths presented in Table 2 showed an accuracy in ±0.02 Å interval with respect to the crystallographic data. The Cu atom in simulations provoked a distorted planar geometry, which was also confirmed by our crystallographic data.

**Figure 4:**
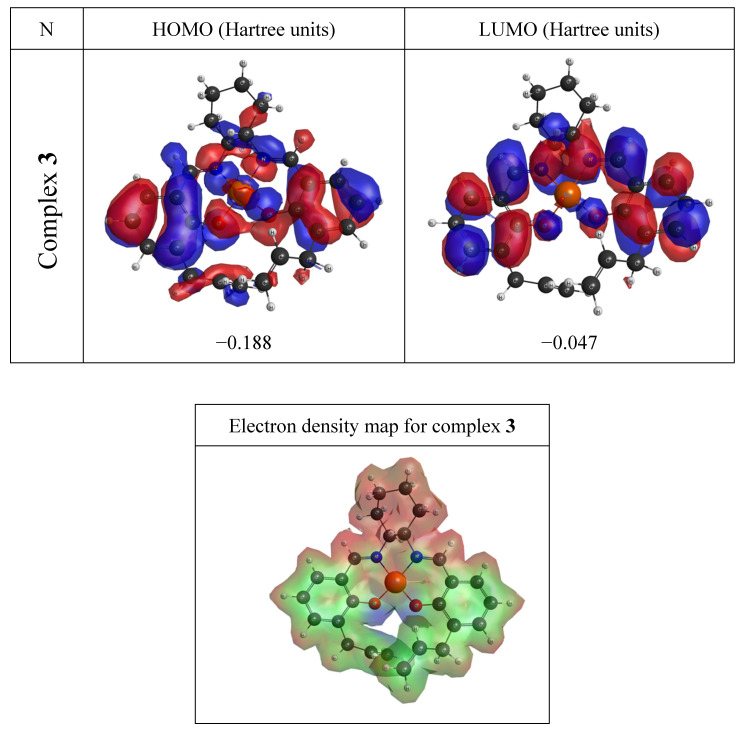
The electron density surfaces of complex **3** including representations of the frontier molecular orbitals (**up**) and of the molecular electrostatic potential and total electronic density (**bottom**).

Figure 4 shows the colored electron density surfaces of complex **3** with molecule electrostatic potential (MEP) values. The MEP is a useful descriptor for understanding which sites in the molecules have affinity with a proton (charge-controlled hard–hard interactions) [34] and the relative polarity of the molecule [35,36,37]. Regions in blue indicate attractive regions, while repulsive regions are in red and regions neutral to positive charges are green.

### 2.6. Evaluation of the Cu(II) and Ni(II) Salen Complexes as Catalysts in the PTC Reaction

**Scheme 2:**
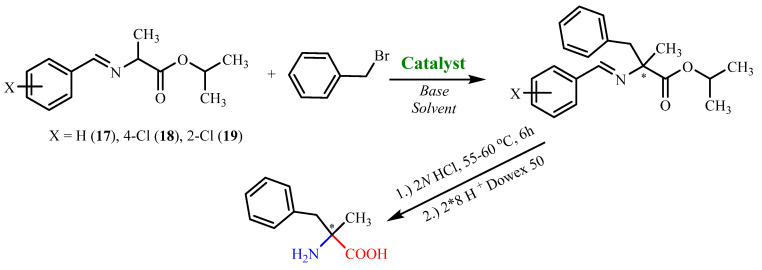
C_α_-alkylation of the amino acid derivatives described in this work.

Subsequently, the synthesized complexes were tested as chiral catalysts in the benchmark reaction of the asymmetric C_α_-alkylation of the amino acid derivative **17** (N-benzylidenealanine isopropyl ester) with benzyl bromide. The choice of this substrate is explained by the consequent formation of a quaternary carbon center and by the potential biological and synthetic importance of the final products, which are unnatural α-substituted analogues of α-amino acids. Remarkably, this reaction was well-studied by Belokon’s group [23,24,25,26,27], but in the present work, we decided to optimize the reaction conditions by increasing the conversion rates of the alkylated product (Scheme 2, Table 3).

Based on the results presented in Table 3, it can be deduced that the optimal conditions for the alkylation reaction are as follows: 1:1.2 substrate:alkylating agent equivalent ratio, 10 mol% of catalyst, 1.5 equivalent NaOH, CH_2_Cl_2_ as solvent (Table 3, entry 7). The reaction was carried out with constant stirring at room temperature for 24 h. Subsequently, the synthesized complexes **1**–**13** were investigated as chiral catalysts in the reaction of C-alkylation of the amino acid derivatives **17** and **18** under the optimized conditions (Table 4). The analysis of the asymmetric synthesis yields indicates that the best result was obtained in the case of substrate **17** using the salen complex **1** which does not contain any substituent in the salicylic fragment (Table 4, entry 1). The bulkier the substituent in the salicylic fragment, the lower the stereoselective yield was. An interesting finding was that when a substituent was present at position 3, >30% yields and low enantiomeric excesses were observed (Table 4, entries 2, 3), but in the case of substituents at positions 3 and 5, both the chemical and the asymmetric yields dropped significantly (Table 4, entries 4, 7, 11, 14). The stereoselectivity also varied depending on the nature of the metal. In fact, after exchanging Cu(II) ion with Ni(II), the stereoselectivity of the obtained product decreased. Our studies have shown that the use of substrate **17** is not practical due to its instability and rapid decomposition. In this regard, we decided to replace the isopropyl ester of N-benzylidenealanine with N-4-Cl-benzylidenealanine isopropyl ester. In fact, this substrate is more stable and can be stored much longer; moreover, the chemical yield of the obtained product was found to have increased. The newly synthesized Schiff base of alanine isopropyl ester and 4-chloro-benzaldehyde was tested as a substrate in the C-alkylation reaction with benzyl bromide (Table 4). Based on our experimental findings, the best results were achieved in the case of the Cu(II) and Ni(II) complexes with unsubstituted salicylic fragment (Table 4, entries 15 and 22).

The same trend previously observed was also found for substrate **18** and in all cases, an increase in both the asymmetric and chemical yields was observed. As expected, the introduction of a chlorine atom into the benzaldehyde fragment of the substrate led to the increase in enantiomeric excesses. Taking the obtained results as a basis, we aimed at investigating the dependence of the asymmetric yield on the nature of the substrate (in particular with regard to the chlorine position in the benzaldehyde fragment). For this reason, the asymmetric C_α_-alkylation reactions of the amino acid isopropyl ester of N-2-chlorobenzylidenealanine (substrate **19**) were studied. The complexes that provided the highest enantiomeric excess (more than 25%) in the case of alkylating substrates **17** and **18** were used as catalysts (Table 5). The data obtained indicate that the presence of chlorine in the *ortho*-position of the substrate leads to increased chemical and asymmetric yields. The complexes that led to the highest asymmetric yields were also tested under low temperature conditions. The results obtained at −20 °C revealed a dramatic increase in the asymmetric yield (Table 4, entries 1 and 15; Table 5, entries 1 and 5). 

**Figure 5:**
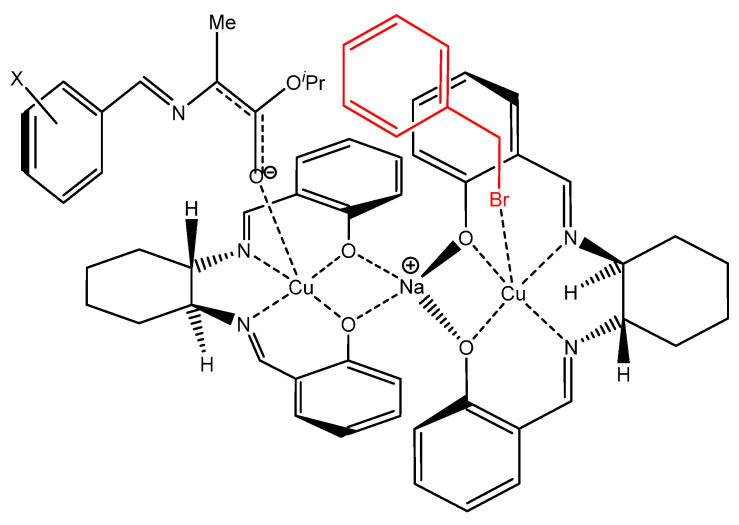
Proposed structure of the complex formed by the carbanion of the substrate and a chiral salen metal complex.

When the Cα-alkylation reaction was carried out at −40 °C, a low (5–8%) yield of the product was observed (data not shown). Overall, we were able to test all the newly synthesized metal complexes as catalysts in the reaction of C_α_-alkylation. The modification of the substrate, and in particular the introduction of chlorine atoms in the *para*- and *ortho*-positions, led to an increase in the stability of the substrate itself and enhanced enantiomeric excesses and chemical yields. In all substrates (**17**–**19**), the switching from Ni(II) complexes to Cu(II) ones also increased the stereoselectivity control. Remarkably, the highest enantiomeric excess was achieved in the case of the simple Cu(II) complex **1** without substituents in the salicylidene fragment. The presence of bulky substituents led to a decrease in the enantioselectivity and chemical yield as well. From the data obtained, it follows that the introduction of substituents of different nature into the aromatic ring of the salicylidene fragment of the metal complexes leads to a decrease in the degree of conversion and in the asymmetric yield of the C_α_-alkylation reaction of the amino acid derivatives. This is obviously caused by an increase in spatial hindrances in the plane of the bis-Schiff base around the coordination sphere of the metal ions. The mechanism of the catalytic action of the salen complexes is at present a matter of conjecture. According to the mechanistic hypothesis previously proposed for this reaction, two units of the complex participate in the form of ionically-bound dimeric binuclear particles in the transition state of alkylation [38]. Nevertheless, the transition state of the alkylation possibly involves both transition and alkali metal ions (Na^+^). Figure 5 illustrates a hypothetical structure of the complex generated from the carbanion of substrates **17**–**19** and complex **1**. 

The salient feature of the complex is the formation of a bimetallic derivative in which the sodium ion is chelated by the oxygen atoms of the salen moiety of the complex, whereas the ionized substrate is coordinated to both the transition metal and the alkali ion. They additionally coordinate both the substrate and the alkylating agent. This resulted in their interaction preferably on one side of the Schiff base plane, which leads to the stereoselective synthesis of the target products, i.e., synthetic α-substituted α-amino acids. The introduction of additional structurally-bulky substituents into the salicylic fragment of metal complexes apparently creates additional spatial hindrance in the coordination plane, which hinders the approach of the reacting particles and leads to a drastic decrease in enantioselectivity. It can also be assumed that the evident increase in the asymmetric yield passing from the substrate **17** (unsubstituted phenyl ring) to **18** and **19** (that contain chlorine atoms at *para*- and *ortho*-positions of the phenyl ring, respectively) is caused by an increase in spatial hindrance around the newly forming chiral center. DFT calculations were used to estimate the charge on Ni and Cu metals (see SI). Our calculations showed that the partial charge of copper is higher than that of nickel, which would make Cu-based complexes able to coordinate the carbanion more efficiently than those containing Ni. In other words, this difference in the charge values could explain why Cu(II) complexes provide greater enantioselectivity and higher chemical yields than Ni(II) complexes.

## 3. Materials and Methods

All reagents were obtained from commercial sources and used without further purification. Thin-layer chromatography (TLC) was carried out on aluminum foil-backed sheets precoated with 0.2 mm Kielselgel 60 F254 (Merck, Darmstadt, Germany). The spots were visualized by UV irradiation (λ = 254 nm). Column chromatography was performed on Fluka silica gel 60 (0.063–0.200 mm, 70–320 mesh) on a glass column. Melting points (mp) were determined by the digital melting point instrument Stuart SMP30 Melting Point (Bibby Scientific Limited, Staffordshire, U.K.). NMR spectra were recorded in deuterated solvents using Varian Mercury Vx 300 MHz (Palo-Alto, CA, USA). Chemical shifts (δ) are reported in parts per million (ppm) relative to tetramethyl silane. Signals were referenced to the residual solvent peak 7.26 (CDCl_3_), 3.31 (CD_3_OD), 4.79 (D_2_O), 2.50 ((CD_3_)_2_SO) for ^1^H and 77.10 (CDCl_3_), 49.15 (CD_3_OD), 39.52 ((CD_3_)_2_SO) for ^13^C NMR spectra. The ^13^C NMR spectra were measured with proton decoupling. Coupling constants are reported in Hertz (Hz). Abbreviations for splitting patterns are as follows: s, singlet; d, doublet; t, triplet; q, quartet; qt, quintet; sext, sextet; hept, heptet; m, multiplet; br., broad. The optical rotation was measured on a Perkin Elmer-341 polarimeter (Waltham, MA, USA). Elemental analysis was performed by the “Euro EA3000” elemental analyzer (Eurovector, Pavia, Italy). For the cation exchange, a Dowex-50 (H+ form) column was used. The chromatographic system used to determine the enantiomeric purity of the amino acids was a Waters Alliance 2695e Separation Module HPLC system equipped with a PDA detector (Waters Corporation, Milford, MA, USA). The separation was accomplished in isocratic mode on a Nautilus-E 5µ” 4.0 × 250 mm column (BioChimMac ST Company, Moscow, Russia) at 30 °C. The mobile phase consisted of methanol and monosodium phosphate buffer (25 mmol/L). The compound’s enantiomeric excess was proved by chiral HPLC analysis of the isolated amino acids. Complexes **1**, **2**, **4** were obtained according to literature procedures [16,17,18]. Infrared spectra were recorded on an IRTracer 100 spectrometer (SHIMADZU, Kyoto, Japan) in the 4000–400 cm^−1^ range using KBr pellets. The thermogravimetric analysis was carried out under helium atmosphere (NETZSCH STA 449 F3 Jupiter, Hanau, Germany). Samples were heated from RT to 500 °C at a rate of 10 °C/min. The X-ray diffraction was performed on a CAD-4 Enraf-Nonius X-ray monocrystal diffractometer (Delft, The Netherlands) working with both low (120–300 K) and high (300–800 K) temperature systems.

### 3.1. (1S,2S)-diaminocyclohexane

It was resolved according to a literature procedure [39] showing a satisfactory elemental analysis and [α]_D_ = +25.215 (c = 5, aq. 1*N* HCl).

### 3.2. Synthesis of the Catalysts

For the synthesis of the complexes **1**–**3**, **5**, **7**, **8**–**10**, **12** and **14**, we added to a (*S*,*S*)-cyclohexanediamine solution in 5 mL methanol the corresponding salicylic aldehyde derivative with a 1:2 molar ratio and stirred for three hours at RT. After the corresponding metal salt (Cu(OAc)_2_xH_2_O or Ni(NO_3_)_2_ × 6H_2_O) was added, the mixture was refluxed for an additional two hours and then filtered. The precipitate was washed with cold methanol. The solid product was collected and dried under vacuum. The synthesis of complexes **4**, **6**, **11** and **13** was accomplished via two steps from corresponding Schiff bases **15** and **16**.

#### 3.2.1. Cu(II) Complex of (1*S*,2*S*)-[N,N’-bis(2’-hydroxy-benzylidene)]-1,2-diaminocyclohexane (**1**)

Chemical yield 75%. Mp dec. 290 °C without melting. Elemental analysis: found, C, 62.77; H, 5.27; N, 7.32; calculated for C_20_H_20_N_2_O_2_Cu: C, 62.57; H, 5.25; N, 7,30.

#### 3.2.2. Cu(II) Complex of (1*S*,2*S*)-[N,N’-bis(2’-hydroxy-3’-methoxy-benzylidene)]-1,2-diaminocyclohexane (**2**)

Chemical yield 41%. Mp dec. 295 °C without melting. Elemental analysis: found, C, 60.02; H, 5.55; N, 6․34; calculated for C_22_H_24_N_2_O_4_Cu: C, 59.52; H, 5.45; N, 6.31.

#### 3.2.3. Cu(II) Complex of (1*S*,2*S*)-[N,N’-bis(2’-hydroxy-3’-allyl-benzylidene)]-1,2-diaminocyclohexane (**3**)

Chemical yield 34%. Mp dec. 300 °C without melting․ Elemental analysis: found, C, 67.33; H, 6.11; N, 6.14; calculated for C_26_H_28_N_2_O_2_Cu: C, 67.29; H, 6.08; N, 6.04.

#### 3.2.4. Cu(II) Complex of (1*S*,2*S*)-N,N’-bis [2’-hydroxy-3’,5’-di-tert-butyl-benzylidene]-1,2-cyclohexanediamine (**4**)

Chemical yield 70%. Mp dec. 292 °C without melting․ Elemental analysis: found, C, 71.11; H, 8.63; N, 4.62; calculated for C_36_H_52_N_2_O_2_Cu: C, 71.07; H, 8.62; N, 4.60.

#### 3.2.5. Cu(II) Complex of (1*S*,2*S*)-[N,N-bis(2’-hydroxy-3’-bromo-benzylidene)]-1,2-diaminocyclohexane (**5**)

Chemical yield 75%. Mp dec. 297 °C without melting. Elemental analysis: found, C, 44.37; H, 3.39; N, 5.19; calculated for C_20_H_18_N_2_O_2_Br_2_Cu: C, 44.34; H, 3.35; N, 5.17.

#### 3.2.6. Cu(II) Complex of (1*S*,2*S*)-[N,N-bis(2’-hydroxy-4’-bromo-benzylidene)]-1,2-diaminocyclohexane (**6**)

Chemical yield 70%. Mp. 294.5 °C. Elemental analysis: found, C, 44.38; H, 3.40; N, 5.20; calculated for C_20_H_18_N_2_O_2_Br_2_Cu: C, 44.34; H, 3.35; N, 5.17.

#### 3.2.7. Cu(II) Complex of (1*S*,2*S*)-[N,N-bis(2’-hydroxy-3’-tert-butyl-5’-bromo-benzylidene)]-1,2-diaminocyclohexane (**7**)

Chemical yield 64%. Mp dec. 272.8 °C with melting․ Elemental analysis: found, C, 51.47; H, 5.29; N, 4.31; calculated for C_28_H_34_N_2_O_2_Br_2_Cu: C, 51.43; H, 5.24; N, 4.28.

#### 3.2.8. Ni(II) Complex of (1*S*,2*S*)-[N,N-bis(2’-hydroxy-benzylidene)]-1,2-diaminocyclohexane (**8**)

Chemical yield 92%. Mp dec. 360 °C without melting. Elemental analysis: found, C, 63.41; H, 5.34; N, 7.41; calculated for C_20_H_20_N_2_O_2_Ni: C, 63.37; H, 5.32; N, 7.39. ^1^H NMR (300 MHz, CDCl_3_): δ 7.31 (s, 2H), 7.19 (ddd, *J* = 8.6, 6.8, 1.9 Hz, 2H), 7.05–6.95 (m, 4H), 6.51 (ddd, *J* = 7.9, 6.7, 1.1 Hz, 2H), 3.50 (s, 1H), 3.25 (s, 2H), 2.42 (s, 1H), 1.93 (s, 2H), 1.35 (d, *J* = 6.0 Hz, 4H) ppm. ^13^C NMR (75 MHz, CDCl_3_): δ 164.7, 157.57, 133.89, 132.7, 121.9, 120.1, 114.9, 96.2, 70.2, 28.8, 24.5 ppm.

#### 3.2.9. Ni(II) Complex of (1*S*,2*S*)-[N,N-bis(2’-hydroxy-3’-methoxy-benzylidene)]-1,2-diaminocyclohexane (**9**)

Chemical yield 97%. Stable under 300 °C. Elemental analysis: found, C, 60.21; H, 5.53; N, 5.61; calculated for C_22_H_24_N_2_O_4_Ni: C, 60.17; H, 5.51; N, 6.38. ^1^H NMR (300 MHz, DMSO-d6): δ 7.35 (s, 2H), 6.67 – 6.54 (m, 4H), 6.33 (t, *J* = 7.8 Hz, 2H), 3.68 (s, 6H), 3.12 (s, 2H), 2.37 (s, 2H), 1.83 (s, 2H), 1.28 (d, *J* = 6.2 Hz, 4H). ^13^ C NMR (75 MHz, CDCl_3_) δ 157.0, 155.1, 150.3, 123.5, 119.1, 113.1, 77.5, 69.4, 54.5, 27.9, 23.7ppm.

#### 3.2.10. Ni(II) Complex of (1*S*,2*S*)-[N,N-bis(2’-hydroxy-3’-allyl-benzylidene)]-1,2-diaminocyclohexane (**10**)

Chemical yield 55%. Mp dec. 350 °C without melting. Elemental analysis: found, C, 68.10; H, 6.19; N, 6.19; calculated for C_26_H_28_N_2_O_2_Ni: C, 68.0; H, 6.15; N, 6.10. ^1^H NMR (300 MHz, CDCl_3_): δ 7.34 (s, 1H), 7.11 (d, *J* = 7.1 Hz, 1H), 6.95 (dd, *J* = 7.9, 1.9 Hz, 1H), 6.54–6.43 (m, 1H), 6.09 (ddt, *J* = 17.0, 10.0, 6.9 Hz, 1H), 5.20–4.99 (m, 3H), 3.43 (d, *J* = 6.9 Hz, 2H), 3.23 (s, 1H), 2.40 (s, 1H), 1.91 (s, 1H), 1.37–1.24 (m, 2H) ppm. ^13^C NMR (75 MHz, CDCl_3_) δ 163.2, 157.5, 137.8, 133.2, 131.8, 130.9, 119.6, 115.3, 114.5, 96.2, 70.2, 34.9, 28.7, 24.5 ppm.

#### 3.2.11. Ni(II) Complex of (1*S*,2*S*)-N,N’-bis[2’-hydroxy-3’,5’-di-tert-butyl-benzylidene]-1,2-cyclohexanediamine (**11**)

Chemical yield 59%. Mp dec. 350 °C without melting. Elemental analysis: found, C, 71.67; H, 8.71; N, 4.69; calculated for C_36_H_52_N_2_O_2_Ni: C, 71.65; H, 8.69; N, 4.64. ^1^H NMR (300 MHz, CDCl_3_): δ 7.40 (s, 1H), 7.31 (d, *J* = 2.6 Hz, 1H), 6.88 (d, *J* = 2.6 Hz, 1H), 2.97 (s, 1H), 2.46 (s, 1H), 1.93 (s, 1H), 1.42 (s, 8H), 1.33 (d, *J* = 6.0 Hz, 2H), 1.27 (s, 9H) ppm. ^13^C NMR (75 MHz, Chloroform-*d*): δ 157.8, 129.1, 126.3, 96.3, 69.9, 35.9, 33.9, 31.5, 29.8, 29.0, 24.6 ppm.

#### 3.2.12. Ni(II) Complex of (1*S*,2*S*)-[N,N-bis(2’-hydroxy-3’-bromo-benzylidene)]-1,2-diaminocyclohexane (**12**)

Chemical yield 55%. Stable under 300 °C; Elemental analysis: found, C, 44.79; H, 3.41; N, 5.25; calculated for C_20_H_18_N_2_O_2_Br_2_Ni: C, 44.74; H, 3.38; N 5.22.

#### 3.2.13. Ni(II) Complex of (1*S*,2*S*)-[N,N-bis(2’-hydroxy-5’-bromo-benzylidene)]-1,2-diaminocyclohexane (**13**)

Chemical yield 99%. Mp dec. 340 °C without melting. Elemental analysis: found, C, 44.76; H, 3.42; N, 5.24; calculated for C_20_H_18_N_2_O_2_Br_2_Ni: C, 44.74; H, 3.38; N, 5.22. ^1^H NMR (300 MHz, DMSO-*d6*): δ 7.75 (s, 2H), 7.61 (d, *J* = 2.7 Hz, 2H), 7.24 (dd, *J* = 9.1, 2.6 Hz, 2H), 6.65 (d, *J* = 9.1 Hz, 2H), 3.13 (s, 1H), 1.78 (s, 2H), 1.29 (d, *J* = 6.1 Hz, 1H), 1.27 (s, 6H) ppm.

#### 3.2.14. Ni(II) Complex of (1*S*,2*S*)-[N,N-bis(2’-hydroxy-3’-tert-butyl-5’-bromo-benzylidene)]-1,2-diaminocyclohexane (**14**)

Chemical yield 57%. Mp dec. 292.8 °C with melting․ Elemental analysis: found, C, 51.89; H, 5.31; N, 4.35; calculated for C_28_H_34_N_2_O_2_Br_2_Ni: C, 51.81; H,5.28; N, 4.32. ^1^H NMR (300 MHz, CDCl_3_): δ 7.30 (s, 2H), 7.23 (d, *J* = 2.7 Hz, 2H), 7.07 (d, *J* = 2.7 Hz, 2H), 3.09 (s, 2H), 2.40 (s, 2H), 1.90 (s, 2H), 1.37 (s, 18H), 1.29 (m, 4H). 13 C NMR (75 MHz, CDCl_3_) δ 163.4, 157.2, 143.5, 133.6, 132.6, 121.8, 105.7, 70.1, 36.0, 29.5, 28.9, 24.4 ppm.

The ligands for complexes **4**, **11** and **6**, **13** were synthesized from (*S*,*S*)-cyclohexanediamine and the corresponding salicylic aldehyde derivatives. To a (*S*,*S*)-cyclohexanediamine solution in 7 mL methanol, the corresponding salicylic aldehyde derivatives with a 1:2 molar ratio were added. The mixture was stirred for 3 h at room temperature. The pale yellow precipitates of ligands were then formed and subsequently filtered, washed with a small amount of methanol and dried under vacuum.

#### 3.2.15. (1*S*,2*S*)-[N,N-bis(2’-hydroxy-3’,5’-tert-butyl-benzylidene)]-1,2-diaminocyclohexane

Chemical yield 94%. Mp 203.5 °C. ^1^H NMR (300 MHz, CDCl_3_): δ 13.69 (s, 1H), 8.30 (s, 1H), 7.31 (d, *J* = 2.5 Hz, 1H), 6.98 (d, *J* = 2.5 Hz, 1H), 3.50 (s, 0H), 3.37–3.25 (m, 1H), 1.93 (dd, *J* = 21.3, 11.2 Hz, 2H), 1.75 (d, *J* = 12.0 Hz, 1H), 1.33 (d, *J* = 53.5 Hz, 16H) ppm. ^13^C NMR (75 MHz, Chloroform-*d*): δ 165.9, 158.1, 140.00, 136.5, 126.8, 126.1, 118.0, 72.5, 35.1, 33.4, 31.5, 29.55, 24.47 ppm.

#### 3.2.16. (1*S*,2*S*)-[N,N-bis(2’-hydroxy-5’-bromo-benzylidene)]-1,2-diaminocyclohexane

Chemical yield 96%. Mp 186.5 °C․ ^1^H NMR (300 MHz, CDCl_3_): δ 13.20 (s, 1H), 8.18 (s, 1H), 7.39–7.23 (m, 1H), 6.80 (d, J = 8.8 Hz, 1H), 3.38–3.26 (m, 1H), 2.00–1.86 (m, 2H), 1.59–1.43 (m, 1H) ppm. ^13^C NMR (75 MHz, CDCl_3_): δ 163.60, 160.11, 135.07, 133.63, 120.02, 119.00, 110.24, 72.79, 33.05, 24.18 ppm.

The 3-allyl-2-hydroxybenzaldehyde for the synthesis of complexes 3 and 10 was prepared from salicylic aldehyde via two steps according to a literature procedure [40].

### 3.3. Catalytic Asymmetric Alkylation Procedure

A flask containing NaOH (100 mg, 2.5 mmol) was filled with argon, then catalysts 1–14 (0.082 mmol), CH_2_Cl_2_ (6 mL) and substrate (200 mg, 0.84 mmol) were added. A solution of 0.1 mL of benzyl bromide was added under stirring, and the reaction proceeded for 24 h at room temperature. Then, the mixture was treated as described earlier for the recovery of the α-amino acids [23,24,25,26]. The isolated amino acids were analyzed by chiral HPLC without any purification.

### 3.4. Schiff Bases of (R,S)-alanine

Substrates 17–19 were synthesized from benzaldehyde, 2-chlorobenzaldehyde, 4-chlorobenzaldehyde and α-alanine isopropyl ester [41].

#### 3.4.1. Isopropyl N-benzylidene-(*R*,*S*)-alaninate (**17**)

Chemical yield 70%, light yellow oil.

^1^H NMR (300 MHz, DMSO-*d6*/CCl_4_): δ 8.29 (br.s, 1H), 7.72–7.76 (m, 2H), 7.37–7.43 (m, 3H), 4.97 (sp, *J* = 6.2 Hz, 1H) ), 4.05 (qd, *J* = 6.7, 0.8 Hz, 1H), 1.43 (d, *J* = 6.7 Hz, 3H), 1.26 (d, *J* = 6.2 Hz, 3H),1.24 (d, *J* = 6.2 Hz, 3H) ppm. ^13^C NMR (75.46 MHz, DMSO/CCl_4_): δ 170.3, 161.6, 135.5, 130.2, 127.9, 67.0, 66.8, 21.25, 21.23, 18.6 ppm.

#### 3.4.2. Isopropyl N-4-chlorobenzylidene-(*R*,*S*)-alaninate (**18**)

Chemical yield 90%, light yellow oil.

^1^H NMR (300 MHz, DMSO-*d6*/CCl_4_): δ 8.29 (br, 1H), 7.72–7.77 (m, 2H), 7.37–7.42 (m, 2H), 4.97 (sp, *J* = 6.2 Hz, 1H), 4.06 (qd, *J* = 6.7, 0.6 Hz, 1H), 1.42 (d, *J* = 6.7, 3H), 1.25 (d, *J* = 6.2 Hz, 3H),1.23 (d, *J* = 6.2 Hz, 3H) ppm. ^13^C NMR (75.46 MHz, DMSO/CCl_4_): δ 170.2, 160.5, 135.8, 134.1, 129.2, 128.1, 67.1, 66.7, 21.2, 21.2, 18.6 ppm.

#### 3.4.3. Isopropyl N-2-chlorobenzylidene-(*R*,*S*)-alaninate (**19**)

Chemical yield 88%, light yellow oil.

^1^H NMR (300 MHz, DMSO-*d6*/CCl_4_): δ 8.70 (br, 1H), 8.05–8.08 (m, 1H), 7.30–7.42 (m, 3H), 4.98 (sp, *J* = 6.1 Hz, 1H), 4.13 (qd, *J* = 6.7,0.8 Hz, 1H), 1.45 (d, *J* = 6.7 Hz, 3H), 1.27 (d, *J* = 6.1 Hz, 3H), 1.25 (d, *J* = 6.1 Hz, 3H) ppm. ^13^C NMR (75.46 MHz, DMSO-*d6*/CCl_4_): δ 170.0, 158.0, 134.5, 132.3, 131.4, 129.1, 128.1, 126.4, 67.2, 66.9, 21.23, 21.20, 18.6 ppm.

### 3.5. Theoretical Studies

The GAMESS-US, Avogadro and the wxMacMolPlt suite of programs were employed to carry out the ab initio and the DFT computations on the Ni and Cu complexes. The geometry optimization of complexes was carried out using the 6–31G(d) basis set for all atoms and Becke’s three-parameter hybrid exchange functional (B3) and the Lee–Yang–Parr correlation available (B3LYP) with a polarized continuum model (PCM) for CH_2_Cl_2_ (dielectric ε = 8.94), which provides a good description of the energy profiles for transition metals complexes and shows results similar to the experimental data [42,43]. As initial states for the geometry optimization, symmetric structures of complexes were used.

## 4. Conclusions

Taking into consideration the increasing attention currently paid to salen complexes, we decided to devote our efforts to further synthetic and characterization studies of chiral Cu(II) and Ni(II) salen complexes. The complexes herein described by us were fully characterized using various physicochemical methods and proved to be suitable phase transfer catalysts for the asymmetric C_α_-alkylation reaction of the Schiff base of the D,L-alanine esters and benzaldehydes. It was found that the introduction of chlorine atoms at the *ortho*- and *para*-positions of the substrate led to increased chemical and asymmetric yields (*ee* up to 98.7%), as well as an enhanced stability of the substrate itself. The highest enantiomeric excess was achieved in the case of a simple Cu(II) complex based on unsubstituted salicylaldehyde at −20 °C. An improvement in the asymmetric yield upon switching from the substrate **17** (unsubstituted phenyl ring) to **18** and **19** (bearing chlorine atoms in *para*- and *ortho*-positions of the phenyl ring, respectively) was shown in our synthetic work. Overall, our findings suggest that a decrease in the spatial hindrance around the newly forming chiral center could be of crucial importance to achieve high stereocontrol in the above-described reactions.

## Figures and Tables

**Table 1:** Thermogravimetric data for complexes **3**, **10** and **13**.

Complex	Residual Weight, %	Start of Weight Loss, T(°C)	End of Weight Loss, T(°C)	Peak (Max) Decomposition, T(°C)
**3**	10.78	300	500	362
**10**	26.99	350	465	384
**13**	56.45	340	460	387

**Table 2:** The crystallographic and DFT data for complex **3**.

Bond	Crystallographic Data (Å)	DFT Data (Å)
Cu-O (I)	1.893(3)	1.913
Cu-O (II)	1.889(3)	1.885
Cu-N (I)	1.924(4)	1.942
Cu-N (II)	1.935(4)	1.947

**Table 3:** Experimental data for the C-alkylation of substrate **17** with benzyl bromide in the presence of complex **1**.

Entry	Catalyst 1 (mol%)	Base (Equiv.)	Time (h)	Conversion (%) *
1	10	NaOH (1.2)	24	74
2	2	NaOH (1.2)	24	traces
3	10	NaOH (1.2)	16	39
4	10	KOH (2.0)	72	73
5	10	KOH (1.2)	24	89
6	5	NaOH (1.5)	24	95
7	10	NaOH (1.5)	24	>99
8	10	NaOH (3.0)	24	95

* Conversion rates were determined by NMR. Reaction conditions: 1:1.2 equivalent ratio of substrate and alkylating agent, solvent CH_2_Cl_2_.

**Table 4:** Experimental outcomes for C-alkylation of amino acid derivatives **17** and **18**.

Catalyst	Entry	17		Entry	18	
		*ee*, ^a^ %	Yield, ^b^ %		*ee*, ^a^ %	Yield, ^b^ %
**1**	1	66.66/90.90 ^c^	65.6/45.5	15	70.2/95.8 ^c^	79.8/60.4
**2**	2	22.44	43.4	16	28.56	42.6
**3**	3	15.67	32.5	17	17.95	50.2
**4**	4	7.32	5.60	18	9.92	7.3
**5**	5	10․12	33.5	19	17	7.3
**6**	6	7.44	7.0	20	15.1	18.5
**7**	7	7.88	5.2	21	14.24	42.5
**8**	8	26.23	41.2	22	37.97	37.4
**9**	9	10.12	25.6	23	20.15	51.6
**10**	10	3.4	36.9	24	17.6	48.9
**11**	11	0	11.2	25	0	70.4
**12**	12	4.08	35.6	26	17.52	9.4
**13**	13	9.89	57.0	27	17.6	8.5
**14**	14	0	4.6	28	0	0.4

^a^ *ee* was determined by chiral HPLC analysis. ^b^ Yields were determined by HPLC analysis. ^c^ Reaction was carried out at −20 °C.

**Table 5:** C-alkylation of amino acid derivative **19**.

Entry	Catalyst	*ee*, ^a^ %	Yield, ^b^ %
1	**1**	78.25/98.38 ^c^	81.3/65.4
2	**2**	27.67	72.3
3	**5**	30.36	35.5
4	**6**	21.53	69.5
5	**8**	42.88/79.78 ^c^	49.4
6	**9**	16.28	72.3
7	**12**	15.6	66.3
8	**13**	10.97	47.3

^a^ *ee* was determined by chiral HPLC analysis. ^b^ Yields were determined by HPLC analysis. ^c^ The reaction was carried out at −20 °C.

## Data Availability

Not applicable.

## Supplementary Materials

The following supporting information can be downloaded at: https://www.mdpi.com/article/10.3390/molecules28031180/s1, Experimental data and other supporting materials are available. References from [23,44,45] are cited from Supplementary Materials.

## Sample Availability

Samples of all the compounds are available from the authors.
