# Supplementary material for: Synthesis, Characterization, and Study of Catalytic Activity of Chiral Cu(II) and Ni(II) Salen Complexes in the α-Amino Acid C-α Alkylation Reaction
Source: Molecules. 2023 Jan 25;28(3):1180. doi: 10.3390/molecules28031180 (PMC9919381; doi:10.3390/molecules28031180)
Supplement: Supplementary file 1 [file molecules-28-01180-s001.zip › molecules-2116618-supplementary.pdf]

Supplementary materials

Synthesis, Characterization, and Study of Catalytic Activity of Chiral Cu(II) and Ni(II) Salen Complexes in the α-Amino Acid C-α Alkylation Reaction

Anna S. Tovmasyan [1], Anna F. Mkrtchyan [1,2,*], Hamlet N. Khachatryan [2], Mary V. Hayrapetyan [2], Robert M. Hakobyan [3], Artavazd S. Poghosyan [1], Avetis H. Tsaturyan [1,2], Ela V. Minasyan [1,2], Victor I. Maleev [4], Vladimir A. Larionov [4,5], Armen G. Ayvazyan [3], Norio Shibata [6], Giovanni N. Roviello [7,*] and Ashot S. Saghyan [1,2,*]

Contents

Materials and methods 2
DFT date of compound 3 2
X-Ray study 4
NMR Spectra 5
HPLC analysis of α-amino acids 20
FT-IR spectroscopy 61

S1. Materials and methods

All reagents were obtained from commercial sources and used without further purification. Thin-layer chromatography (TLC) was carried out on Merck aluminum foil backed sheets precoated with 0.2 mm Kielselgel 60 F_{254}. The spots were visualized by UV irradiation (λ 254nm). Column chromatography was performed on Fluka silica gel 60 (0.063-0.200 mm, 70-320 mesh) on a glass column. Melting points (mp) were determined by «Electrothermal». ^1H and ^{13}C NMR spectra («Mercury-300 Varian» 300 MHz) were recorded using TMS as an internal standard (0 ppm). The NMR spectra were calibrated by solvent at 7.26 ($CDCl_3$), 3.31 (D_3COD), 4.79 (D_2O), 2.50 ($(CD_3)_2SO$) for ^1H and 77.23 ($CDCl_3$), 49.15 (D_3COD), 39.52 ($(CD_3)_2SO$) for ^{13}C NMR spectra. The ^{13}C NMR spectra were measured with proton decoupling. The optical rotation was measured on a Perkin Elmer-341 polarimeter. Elemental analysis was done by Euora EA3000. For the cation exchange column Dowex-50 (H^+ form) was used. The chromatographic system used for enantiomeric purity of the amino acids was a Waters alliance 2695e Separation Module(USA) HPLC system equipped with PDA detector. Separation was done in isocratic mode on a Nautilus-E 5μ" 4.0 x 250mm column (BioChimMac ST Company, Moscow, Russia) at 30˚C temperature. The mobile phase consisted of methanol and monosodium phosphate buffer (25mmol/L) with composite enantiomeric yield was proved by chiral HPLC analysis of the isolated amino acids. **1,2,4** complexes were obtained according to the literature data[i]. Infrared spectra were recorded on a SHIMADZU FT-IR spectrometer in the range of 4000-400 cm^{-1} using KBr pellets. The thermogravimetric analysis was carried out under the helium atmosphere (NETZSCH STA 449 F3 Jupiter). Samples was heated from RT to 500°C with 10°C/min speed. The X-Ray was done by Enraf-Nonius CAD4.

S2. DFT date of compound 3

Complex 3

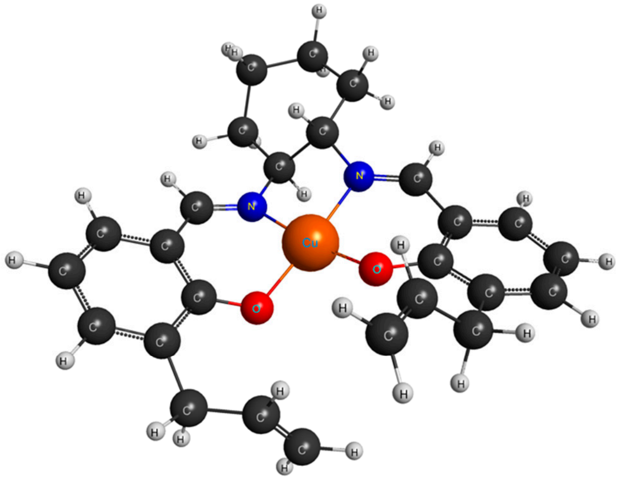

C	6.0	-1.68766785	-2.47320819	3.74538803
C	6.0	-2.16050959	-1.01010430	3.77816725
C	6.0	-0.25685608	-2.62887836	3.17264986
H	1.0	-2.36486673	-3.03395510	3.09425378
H	1.0	-1.78365588	-2.92849112	4.73486185
C	6.0	0.35647342	-1.28798378	2.67286181
N	7.0	0.81473732	-3.17477441	4.03697252
H	1.0	-0.29497790	-3.29883337	2.30259490

C	6.0	-2.02625847	-0.37470841	2.38992238
H	1.0	-1.59392214	-0.44132146	4.52359486
H	1.0	-3.20870447	-0.97944170	4.10346031
C	6.0	-0.56948900	-0.39381048	1.84018397
N	7.0	1.65726995	-1.66311193	2.09929228
H	1.0	0.59223050	-0.72069329	3.58748031
H	1.0	-0.58168286	-0.69188911	0.78569609
H	1.0	-0.14173548	0.61536425	1.87318850
H	1.0	-2.66954327	-0.93100429	1.70153606
H	1.0	-2.40614414	0.65294939	2.40032768
C	6.0	2.02222061	-1.39323199	0.88523221
C	6.0	0.70916343	-3.39668918	5.30599117
C	6.0	6.97214031	-1.99867976	1.56043148
C	6.0	5.73842716	-1.91552532	0.67411399
C	6.0	4.41208982	-2.07154679	1.20195055
O	8.0	4.24027443	-2.48707938	2.42017722
C	6.0	3.31975532	-1.68495798	0.34393296
C	6.0	3.53330135	-1.46145153	-1.03593087
H	1.0	2.67023516	-1.24330354	-1.66627228
C	6.0	4.80360985	-1.51757324	-1.56889760
H	1.0	4.97370052	-1.38845634	-2.63213682
C	6.0	5.89244843	-1.68450701	-0.68790126
H	1.0	6.90305662	-1.62778342	-1.09147990
H	1.0	1.33768809	-0.86722159	0.21672024
H	1.0	2.43789744	-4.04440403	9.50389957
C	6.0	2.58375072	-4.12746811	8.43091202
C	6.0	1.58423543	-3.76306081	7.55246162
H	1.0	0.62010229	-3.41239524	7.91899300
C	6.0	1.79159534	-3.82200837	6.15504980
C	6.0	3.05032825	-4.27011490	5.61760378
O	8.0	3.30090356	-4.29168797	4.33810568
C	6.0	4.03552341	-4.74932003	6.54542494
C	6.0	5.28055143	-5.49378061	6.08207464
C	6.0	3.79404092	-4.62309694	7.90844393
H	1.0	4.57074928	-4.94059706	8.60241890
H	1.0	-0.25229955	-3.24719667	5.80511808
Cu	29.0	2.56672549	-2.96969295	3.21260953
C	6.0	6.85703802	-1.43705022	2.96568608
C	6.0	5.99957800	-5.03320169	4.84058714
C	6.0	7.77194929	-1.63881767	3.91768837
C	6.0	6.10075474	-5.78499365	3.74418998
H	1.0	7.77398157	-1.44898367	1.04099631
H	1.0	7.33461761	-3.03672814	1.62651920
H	1.0	4.99890661	-6.54284620	5.92149973
H	1.0	5.98318863	-5.50473738	6.92473459
H	1.0	5.98555851	-0.82985163	3.18332744
H	1.0	8.65371895	-2.26229405	3.74154925
H	1.0	7.67193890	-1.19562542	4.91045094
H	1.0	6.44925785	-4.03918982	4.85893440
H	1.0	5.62851954	-6.76626348	3.69480300
H	1.0	6.60486841	-5.44049120	2.84399343

S3. X-Ray study

The unit cells parameters of crystals of the compounds **3** were measured on an Enraf-Nonius automated diffractometer CAD-4 at room temperature using the diffraction angles of 24 reflections. The diffraction experiment was performed on the same diffractometer using graphite monochromator, Mo-Kα radiation, $\theta/2\theta$-scan The initial structure model was determined by direct methods and the positional parameters of non-hydrogen atoms were refined together with anisotropic thermal parameters using the software package SHELXTL[ii]. The absorption correction was made by psi-scan method[iii]. The hydrogen atoms were positioned geometrically and refined using riding model, with C-H=0.93÷0.97Å, $U_{iso}(H)=1.2U_{eq}(C)$. Crystallographic and experimental data are listed in table 1.

The full crystallographic data in CIF format are available at: http://www.ccdc.cam.ac.uk/products/csd/request/ (free of charge), deposition number are CCDC 2120026.

Crystallographic and experimental data

Crystal Data			
Compound	**3**		
Formula	$C_{26}H_{28}N_2O_2Cu$		
Formula Weight	464.04		
Crystal System	triclinic		
Space group	P-1		
a, b, c [Å]	7.7142(15)	12.750(3)	13.306(3)
α, β, γ [deg]	112.91(3)	103.62(3)	100.64(3)
V [Å^3]	1114.4(6)		
Z	2		
D(calc) [g/cm^3]	1.383		
μ(MoKα) [mm^{-1}]	1.005		
F(000)	486		
Crystal Size [mm]	0.4×0.1×0.1		
Data Collection			
Temperature (K)	293		
Radiation [Å]	MoKα 0.71073		
θ_{min}, θ_{max} [Deg]	1.8, 27.0		
Dataset	$-9\le h\le 9$; $-16\le k\le -16$; $-16\le l\le 16$		
Tot., Uniq. Data, R(int)	10418, 4842, 0.040		
Observed data [I > 2.0 σ(I)]	3640		
Refinement			
Nref, Npar	4842, 317		
R, wR2, S	0.0639, 0.1681, 1.13		

S4. NMR Spectra

Complex 8. H1 NMR (300 MHz, Chloroform-*d*)

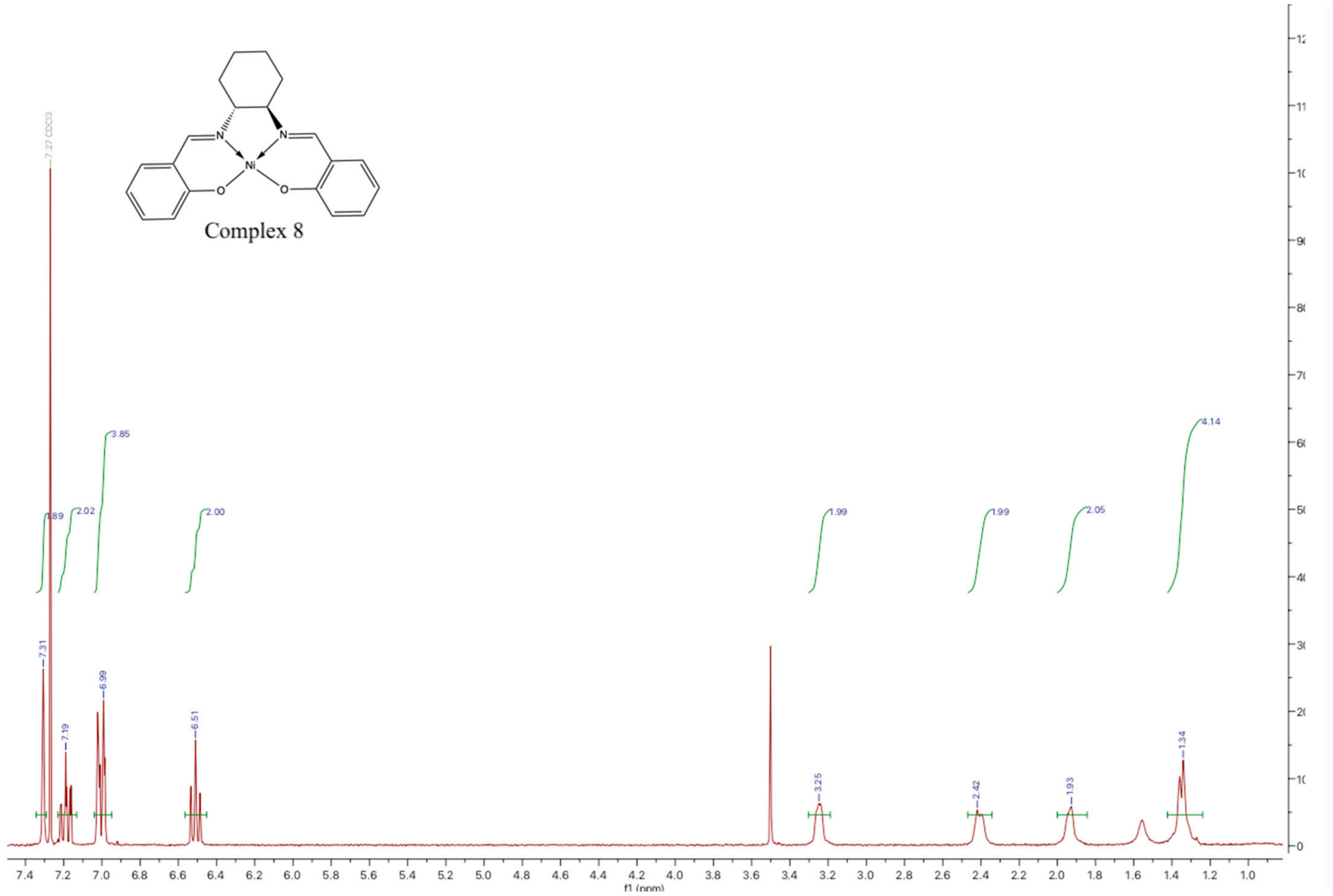

Complex 8. C13 NMR (75 MHz, Chloroform-*d*)

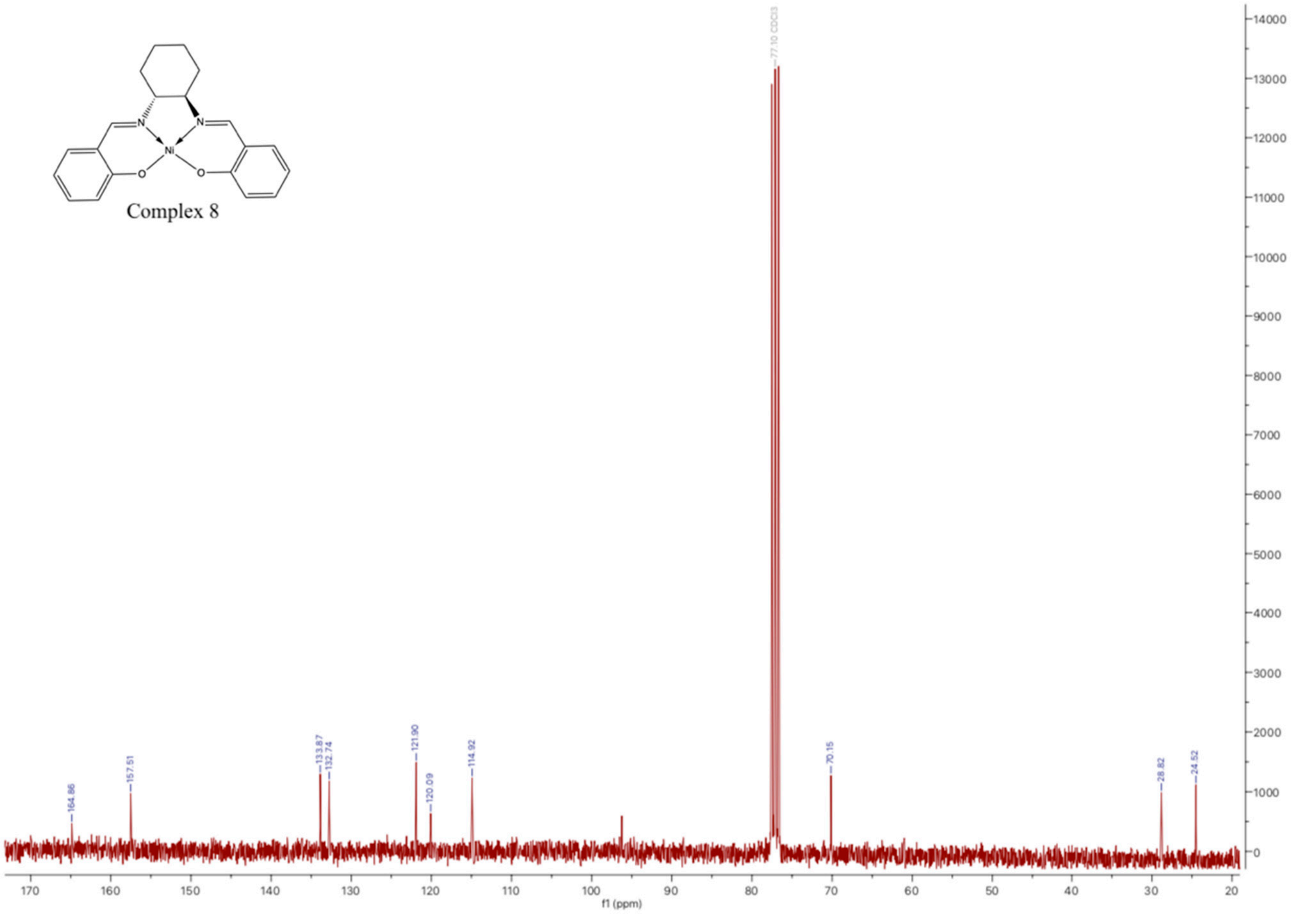

Complex 9. H1 NMR (300 MHz, DMSO-d_6)

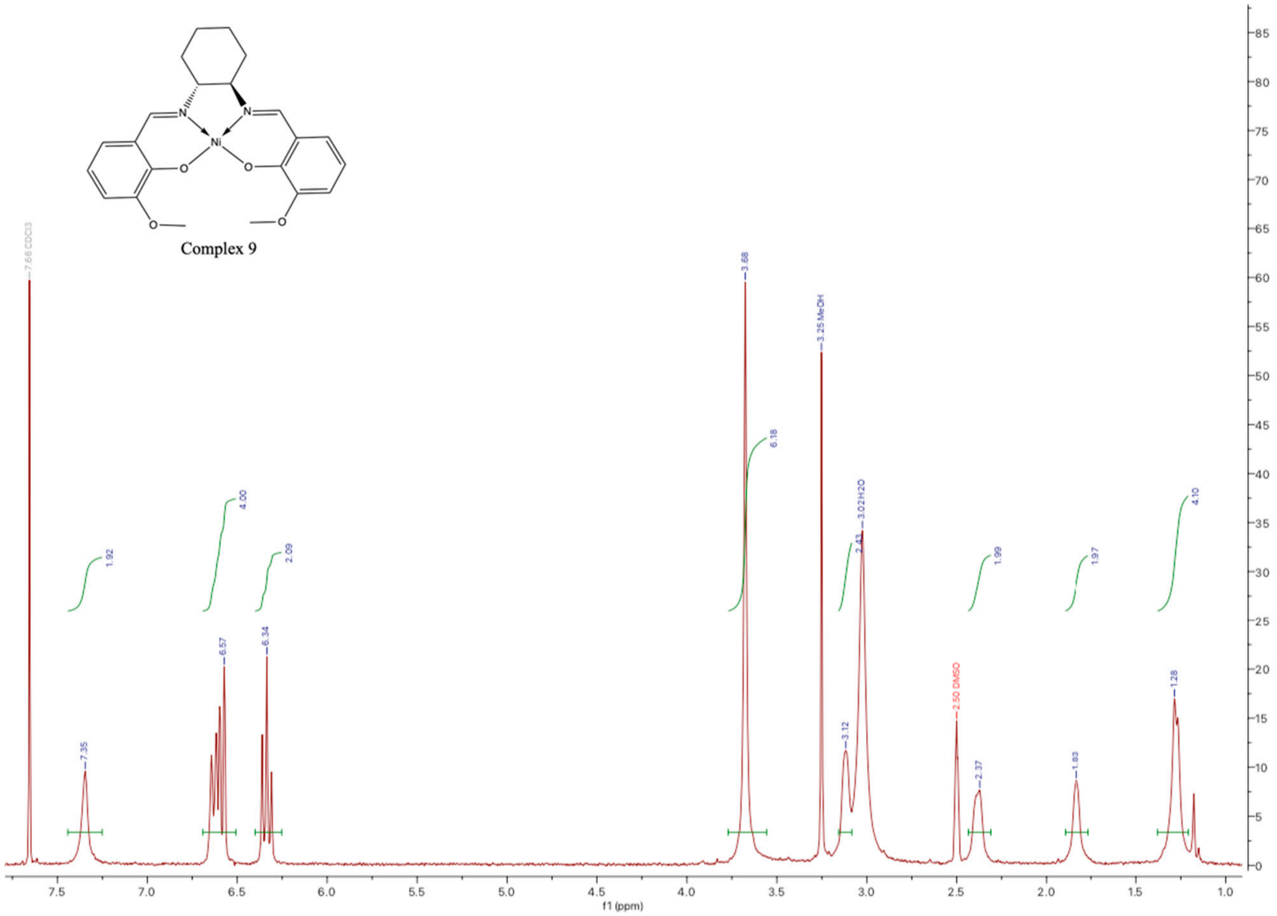

Complex 9. C13 NMR (75 MHz, DMSO-d_6)

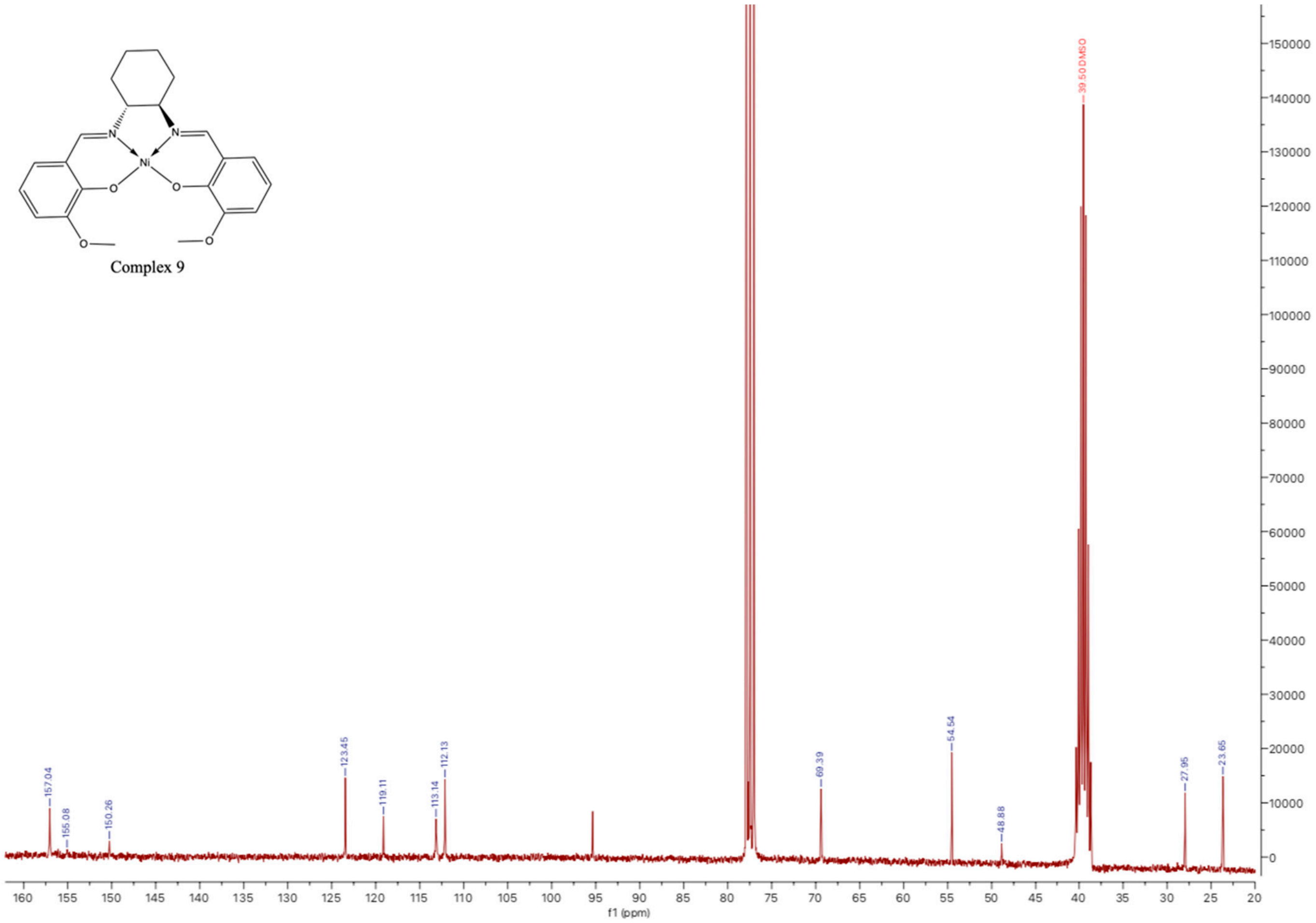

Complex 10. H1 NMR (300 MHz, Chloroform-*d*)

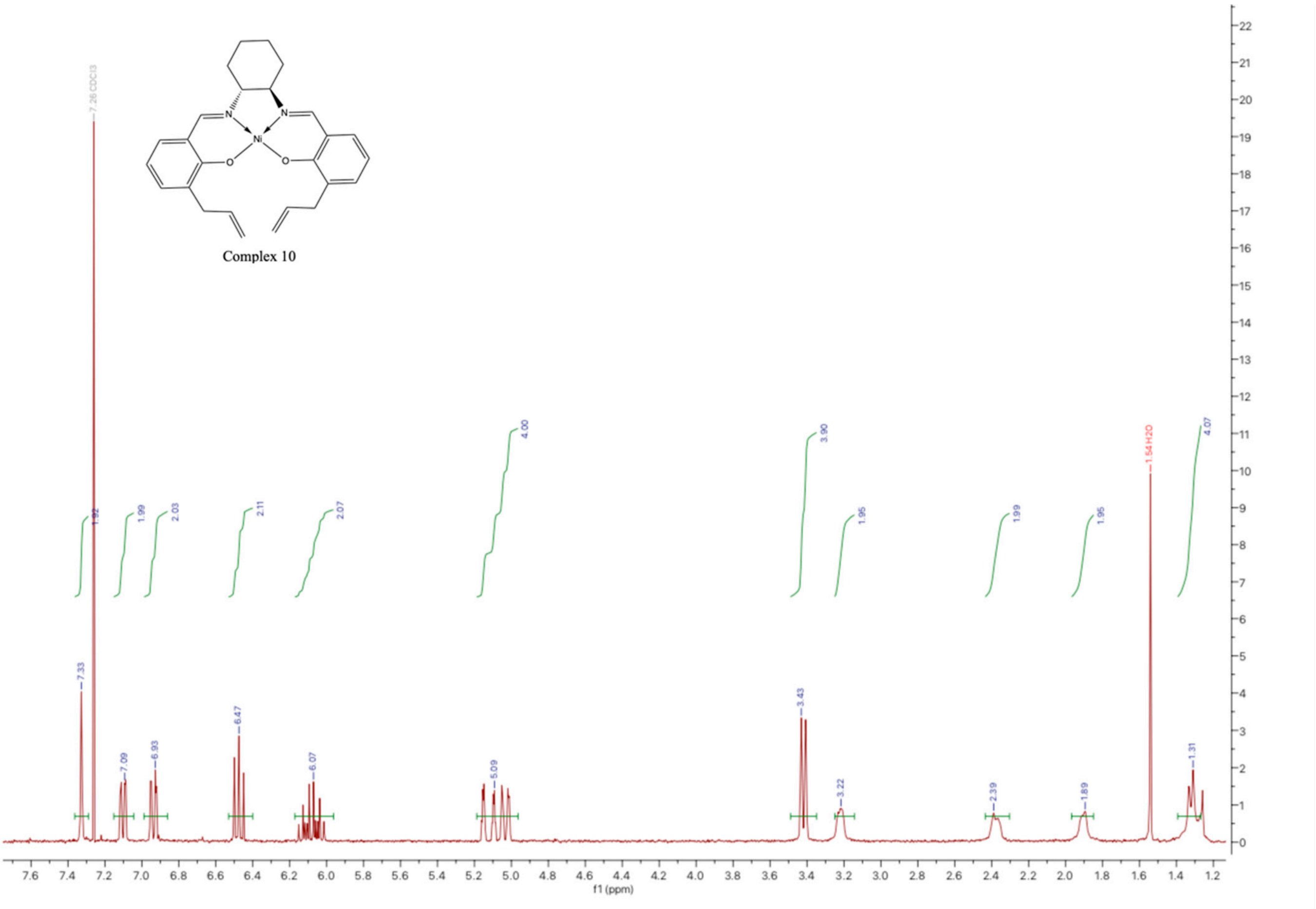

Complex 10. C13 NMR (75 MHz, Chloroform-*d*)

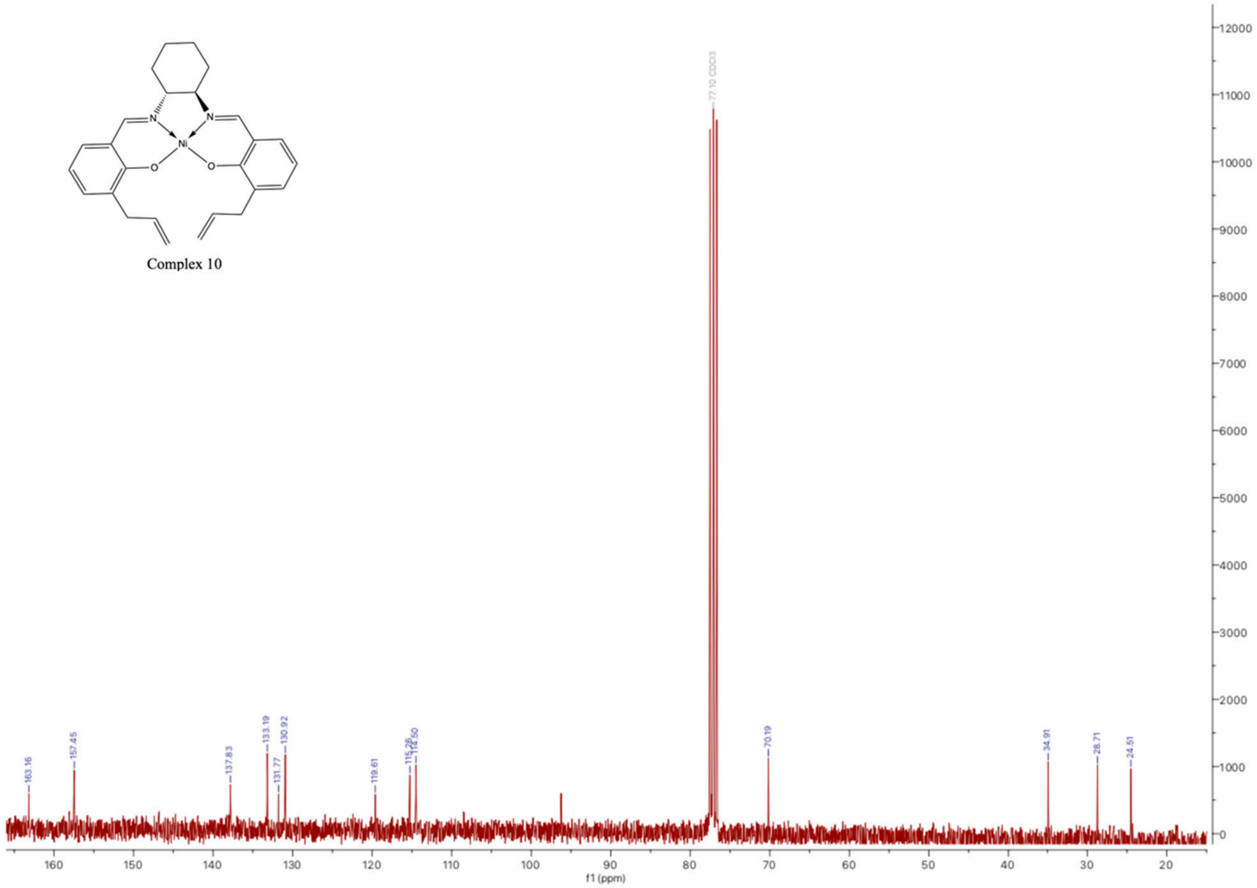

Complex 11. H1 NMR (300 MHz, Chloroform-*d*)

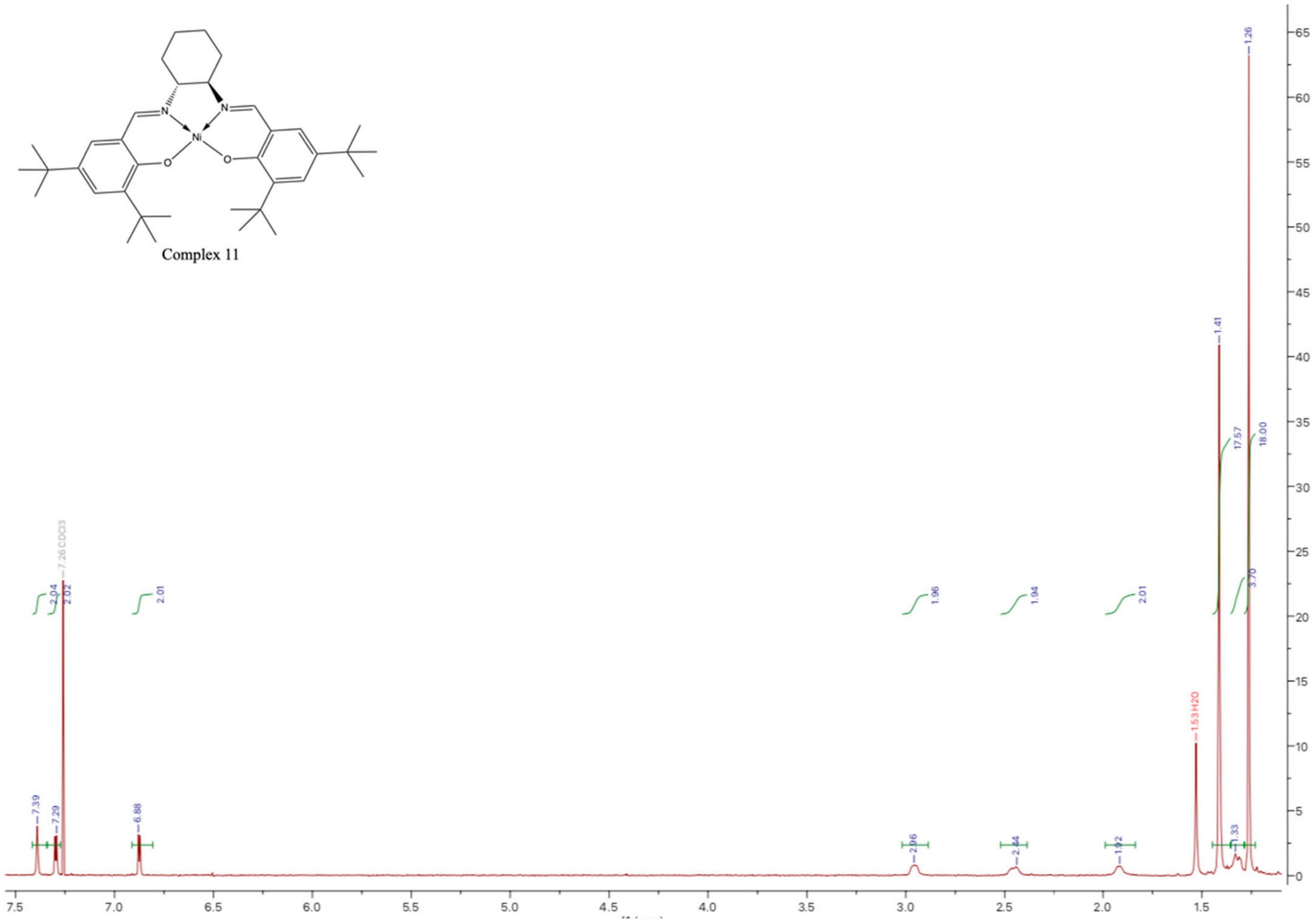

Complex 11. C13 NMR (75 MHz, Chloroform-*d*)

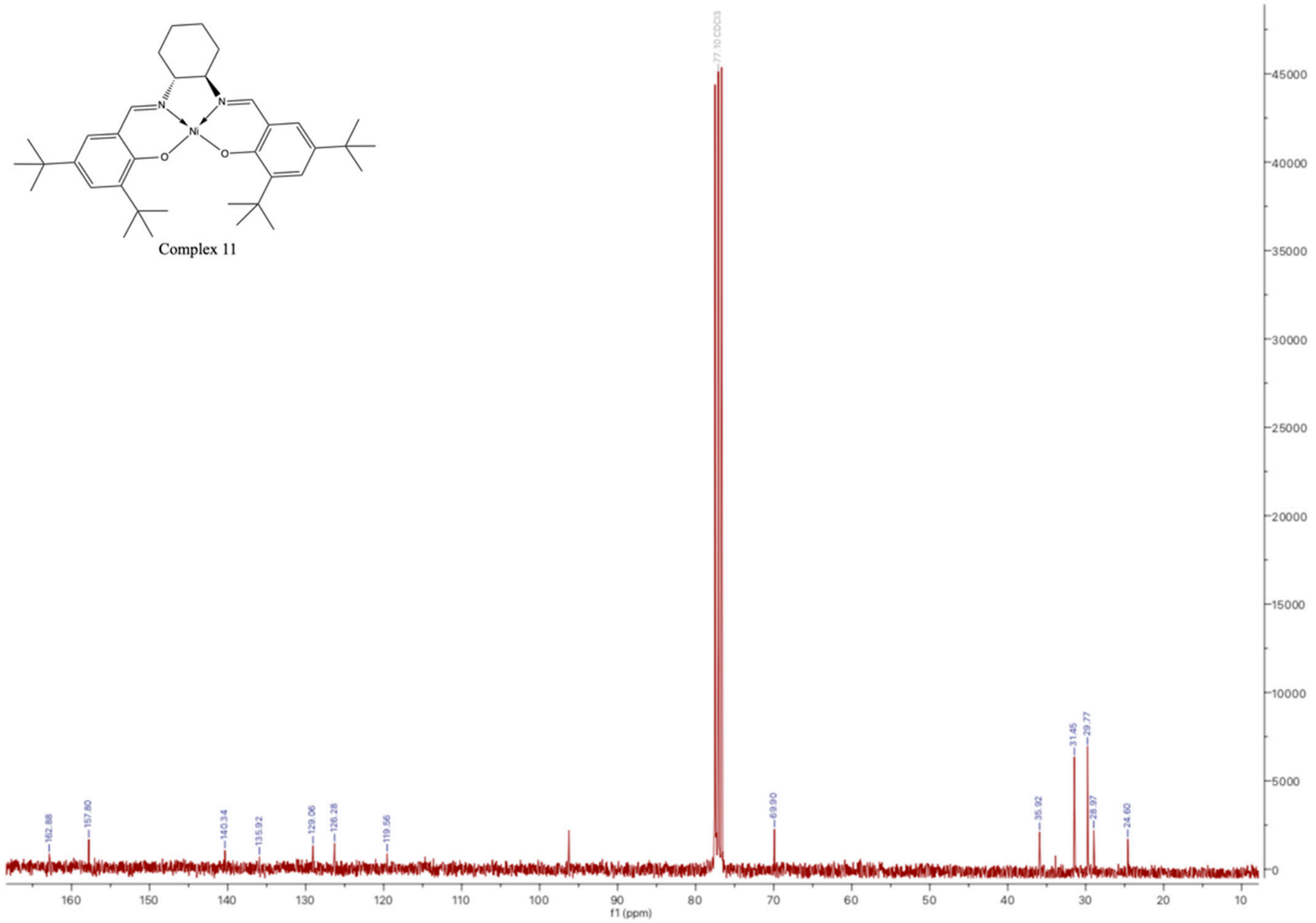

Complex 13. H1 NMR (300 MHz, DMSO-d_6)

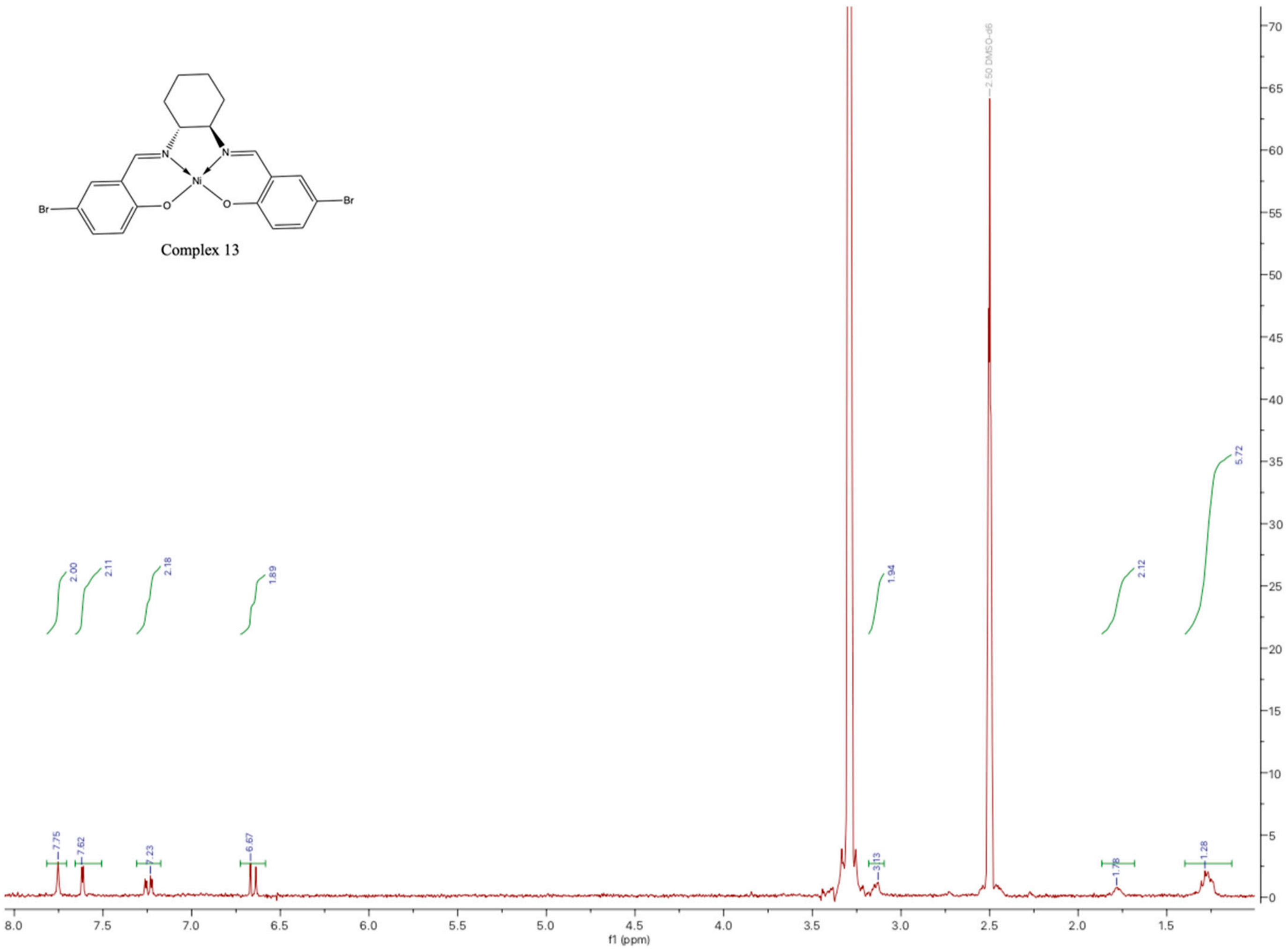

Complex 14. H1 NMR (300 MHz, Chloroform-*d*)

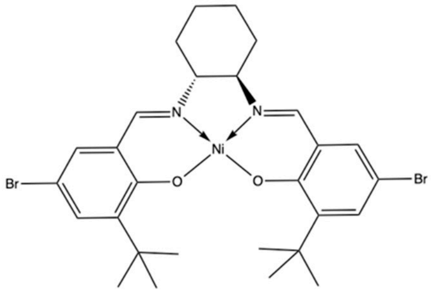

Complex 14

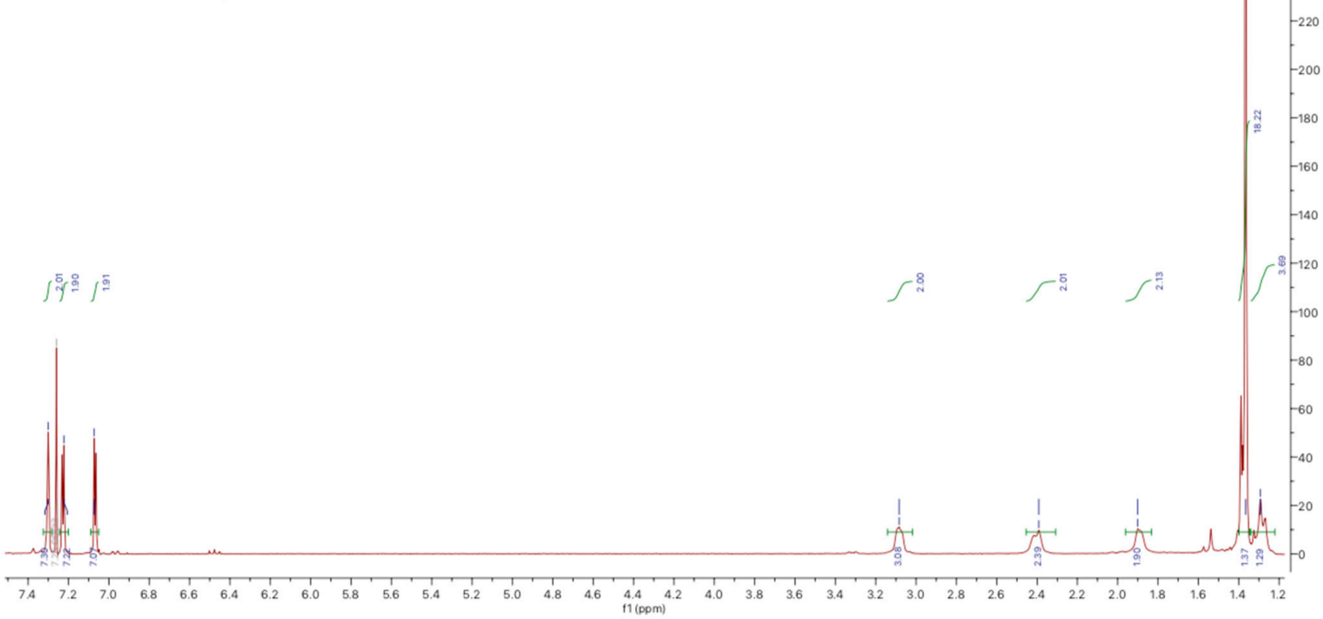

Complex 14. C13 NMR (75 MHz, Chloroform-*d*)

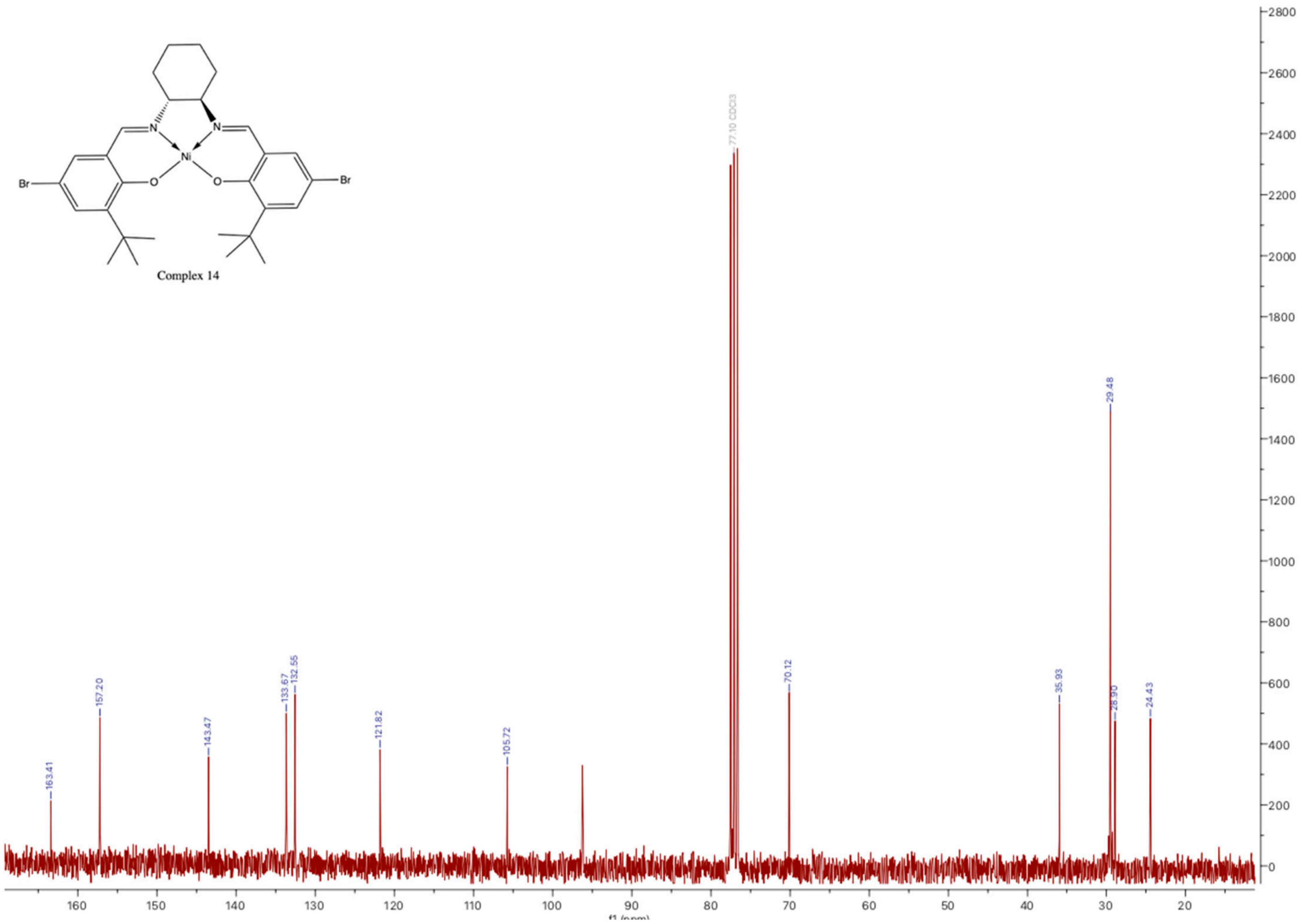

Complex 14. Dept NMR (300 MHz)

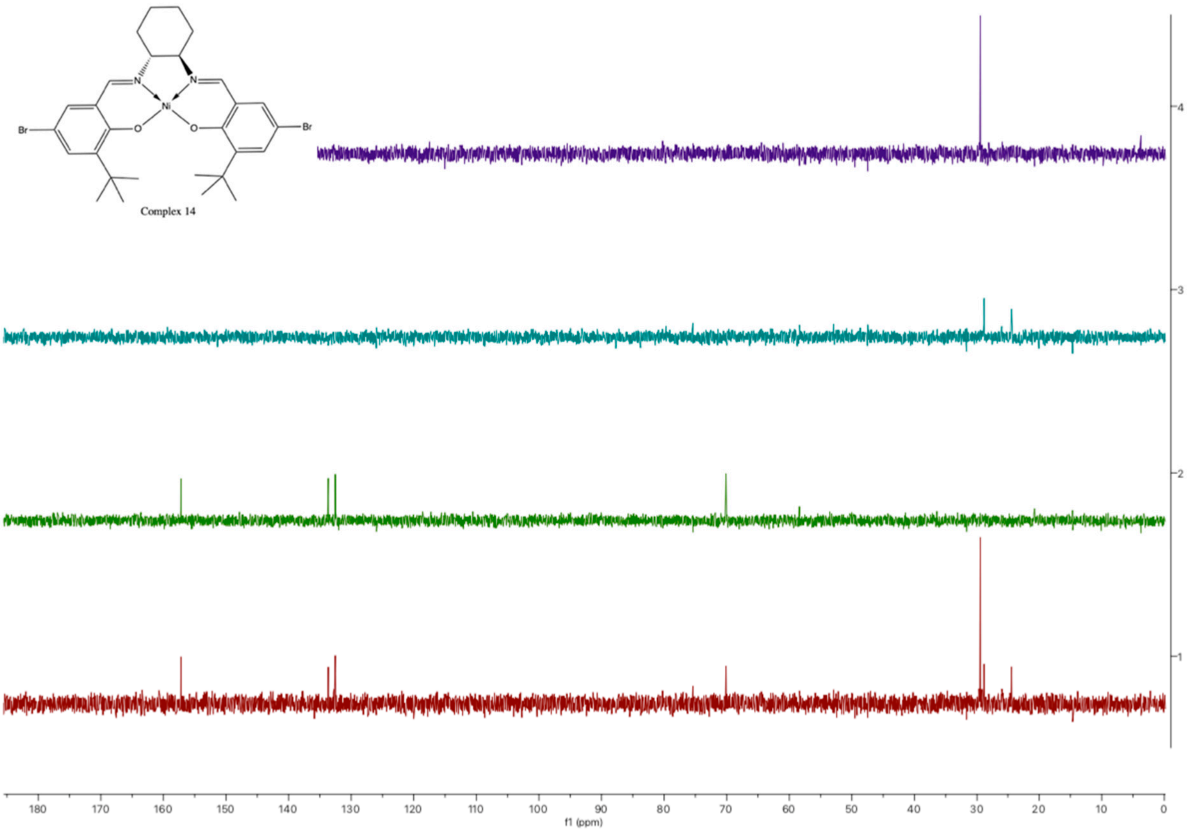

Substrate 17; ^{1}H NMR, DMSO/CCl_4

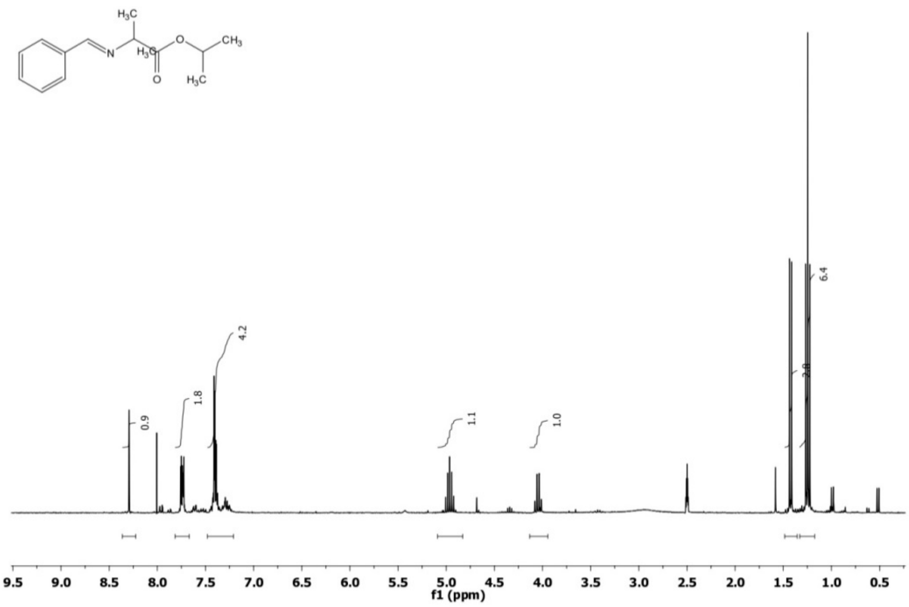

Substrate 17; ^{13}C NMR, DMSO/CCl_4

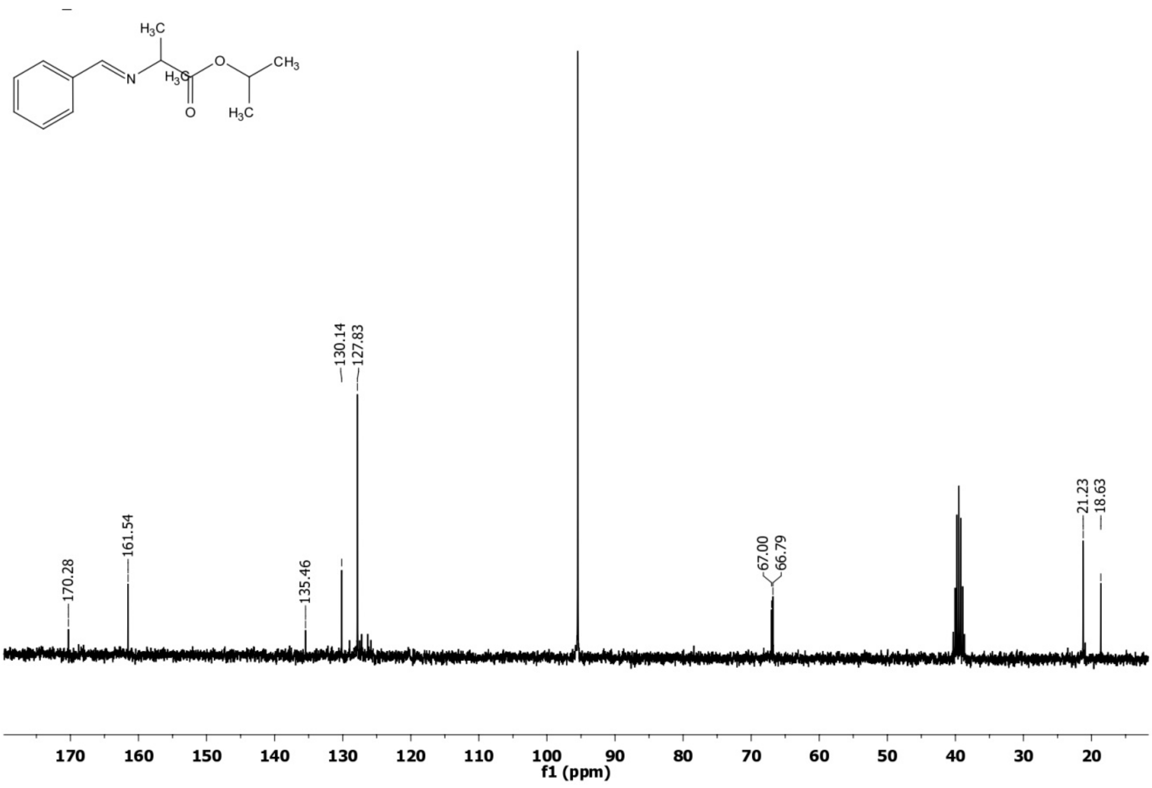

Substrate 18, ^{1}H NMR, DMSO/CCl_4

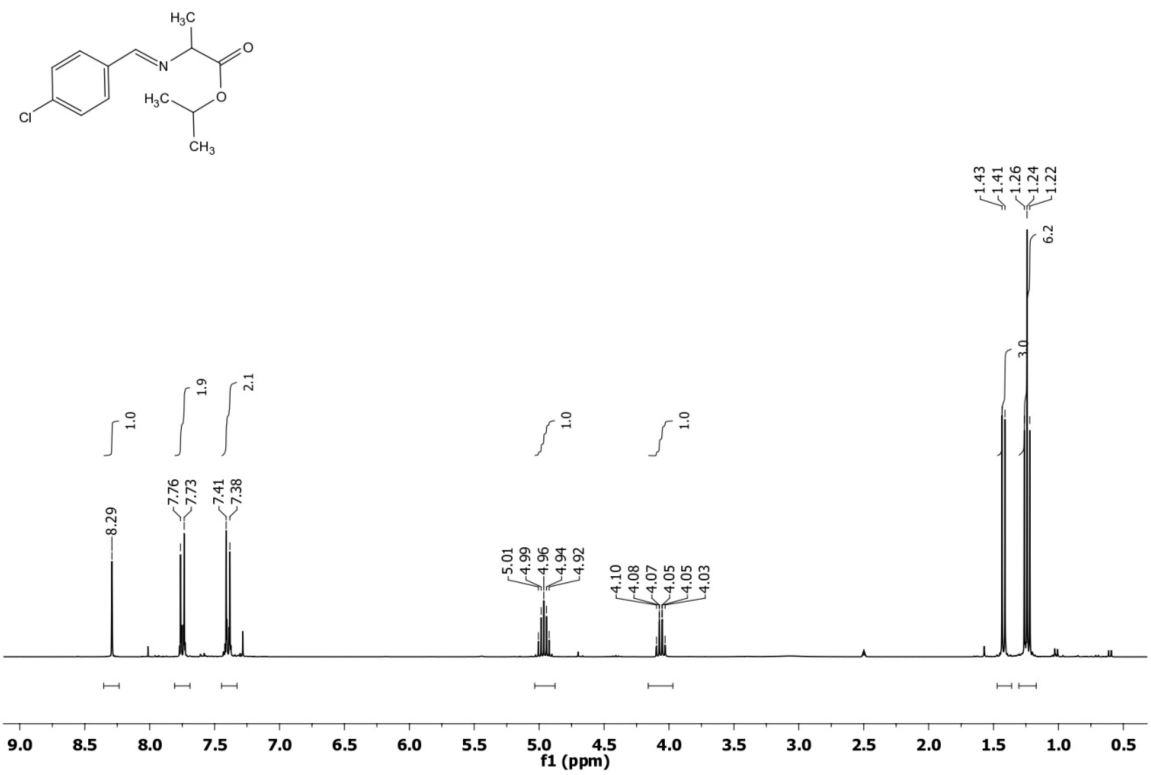

Substrate 18, ^{13}C NMR, DMSO/CCl_4

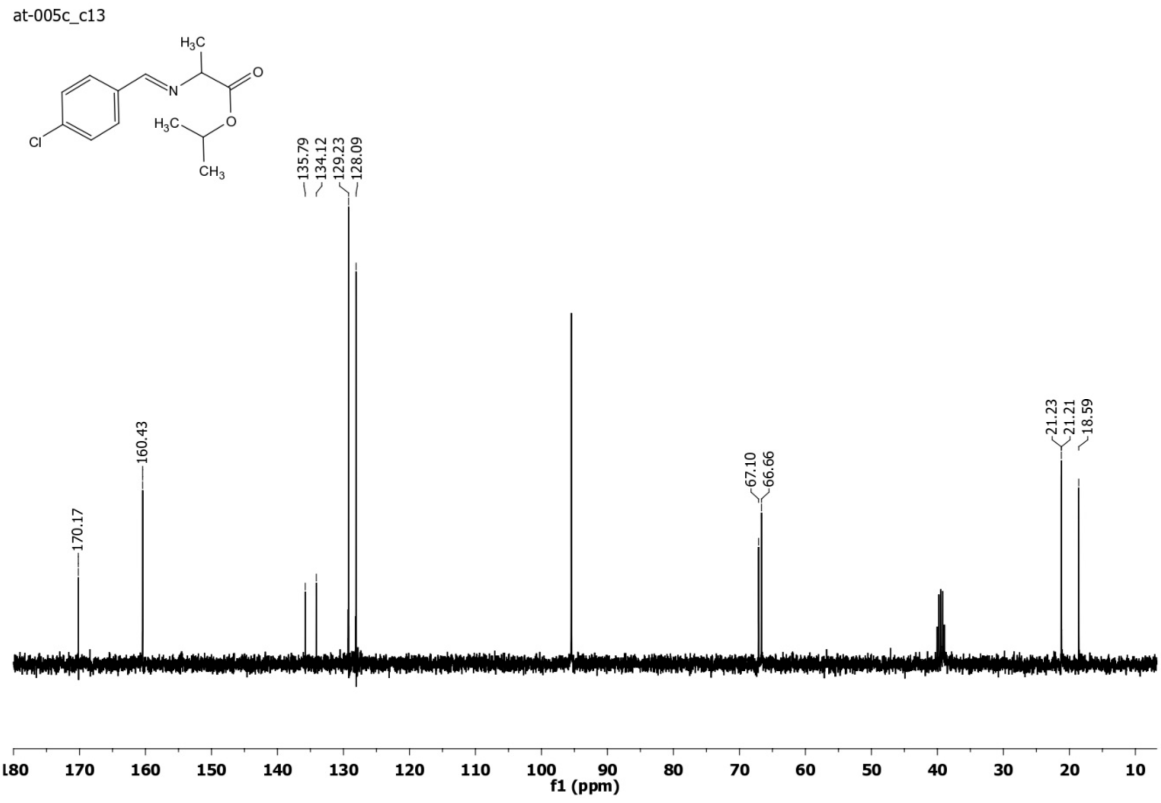

Substrate 19, ^{1}H NMR, DMSO/CCl_4

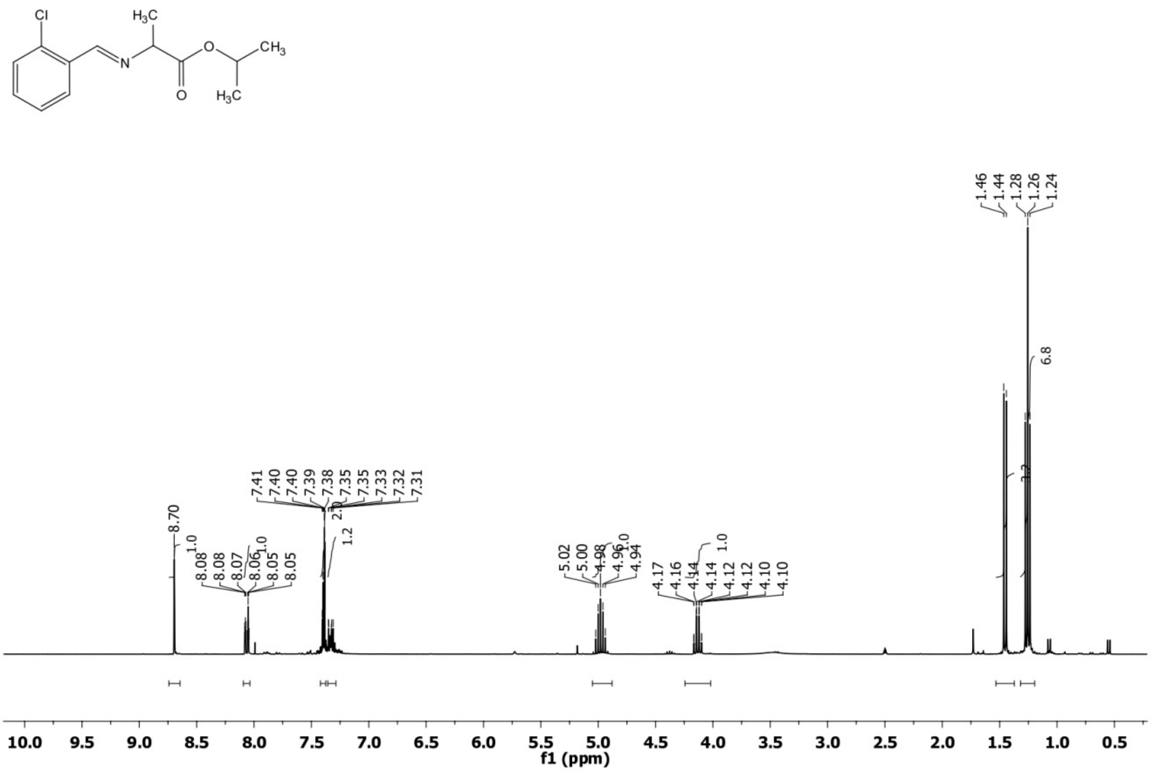

Substrate 19, ^{13}C NMR, DMSO/CCl_4

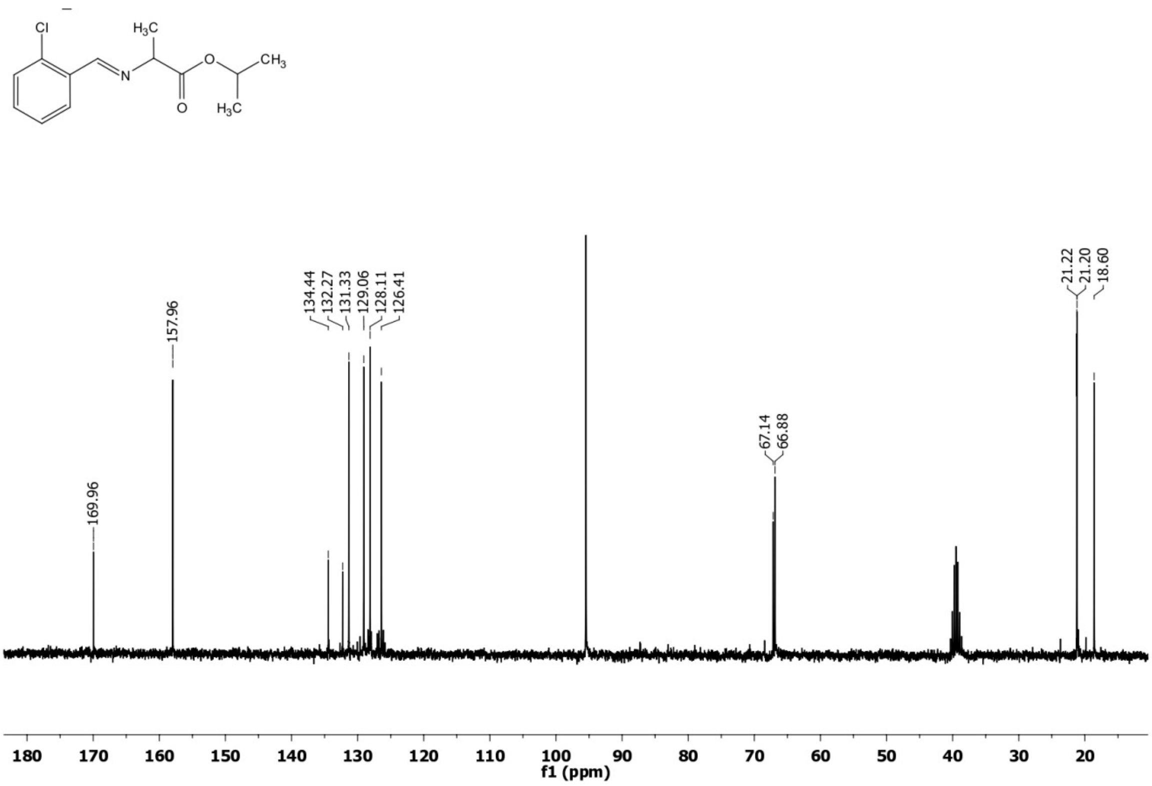

S5. HPLC analysis of α-amino acids

Nautilus-E 5µ" 4.0 x 250mm column (BioChimMac ST Company, Moscow, Russia).

Table 3.

S5.1. Cα-Alkylaton of substrate 17.

Sample name:Racemate, *(S,R)*-Me-Phenylalanine

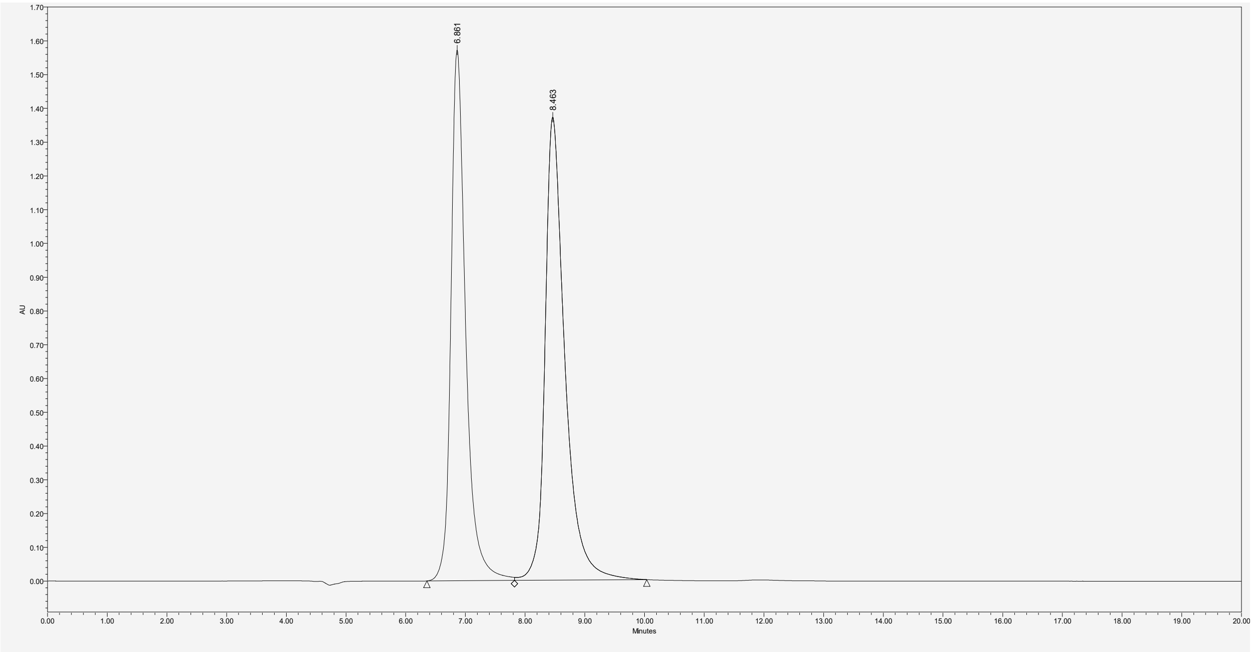

	Name	Retention Time	Area	% Area	Height
1	*(S)*-α-Me-Phe	6.861	27060139	46.01	1571897
2	*(R)*-α-Me-Phe	8.463	31756877	53.99	1371294

Complex №1
ee% =66.66%

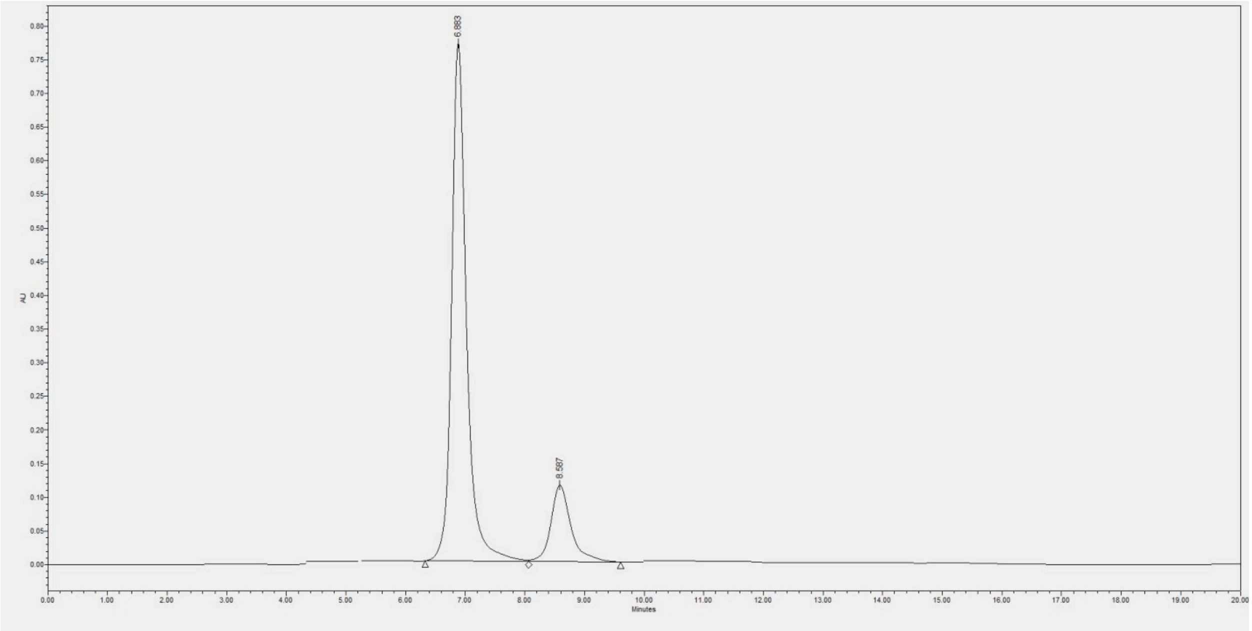

	Name	Retention Time	Area	% Area	Height
1	*(S)*-α-Me-Phe	6.883	13285255	83.33	769083
2	*(R)*-α-Me-Phe	8.587	2657350	16.67	113527

Complex №1, -20 ^{0}C

ee% =90.9%

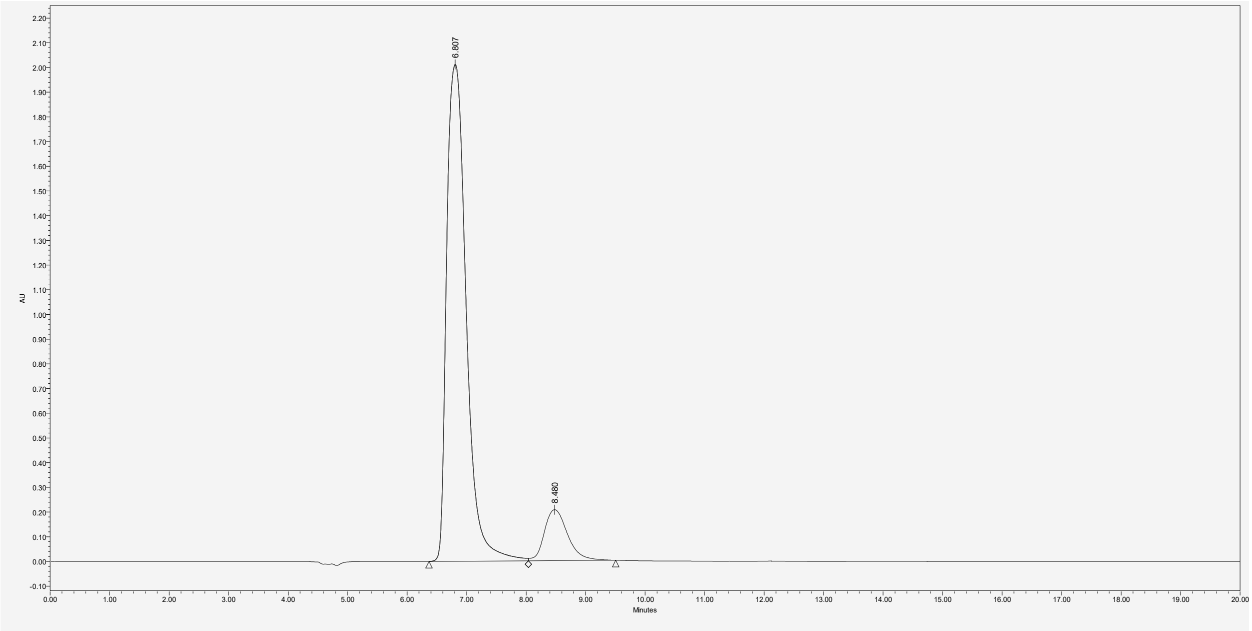

	Name	Retention Time	Area	% Area	Height
1	*(S)*-α-Me-Phe	6.807	49112287	95.45	2117294
2	*(R)*-α-Me-Phe	8.48	2341130	4.55	100929.1

Complex №2
ee% =22.44 %

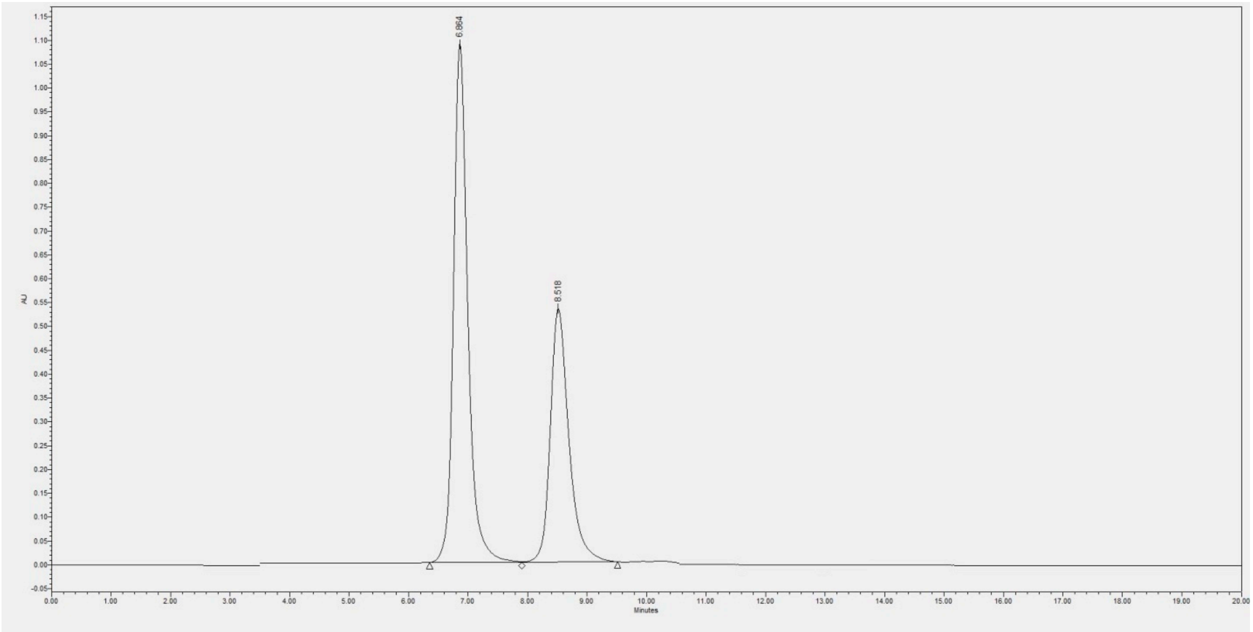

	Name	Retention Time	Area	% Area	Height
1	*(S)*-α-Me-Phe	6.864	18159383	61,22	1086931
2	*(R)*-α-Me-Phe	8.518	11501026	38,78	531424

Complex №3
ee%=15.67%

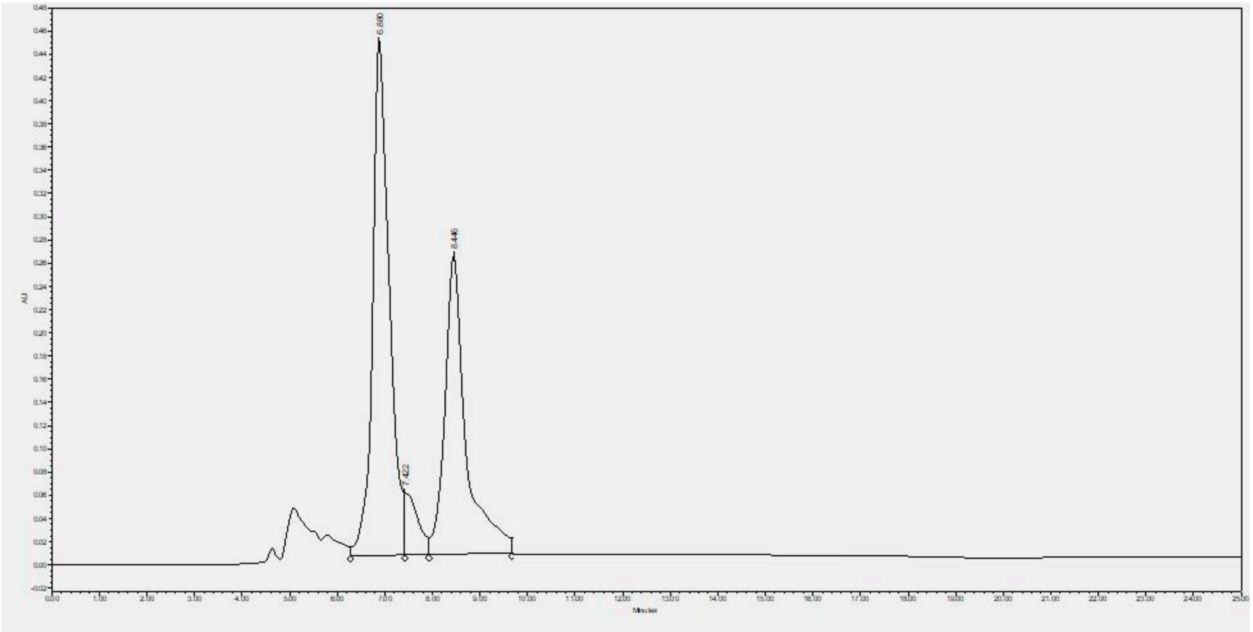

	Name	Retention Time	Area	% Area	Height
1	(S)-α-Me-Phe	6.880	10810719	57.83	442774
2	(R)-α-Me-Phe	8.446	7881017	42.17	256460

Complex №4
ee%=7.32%

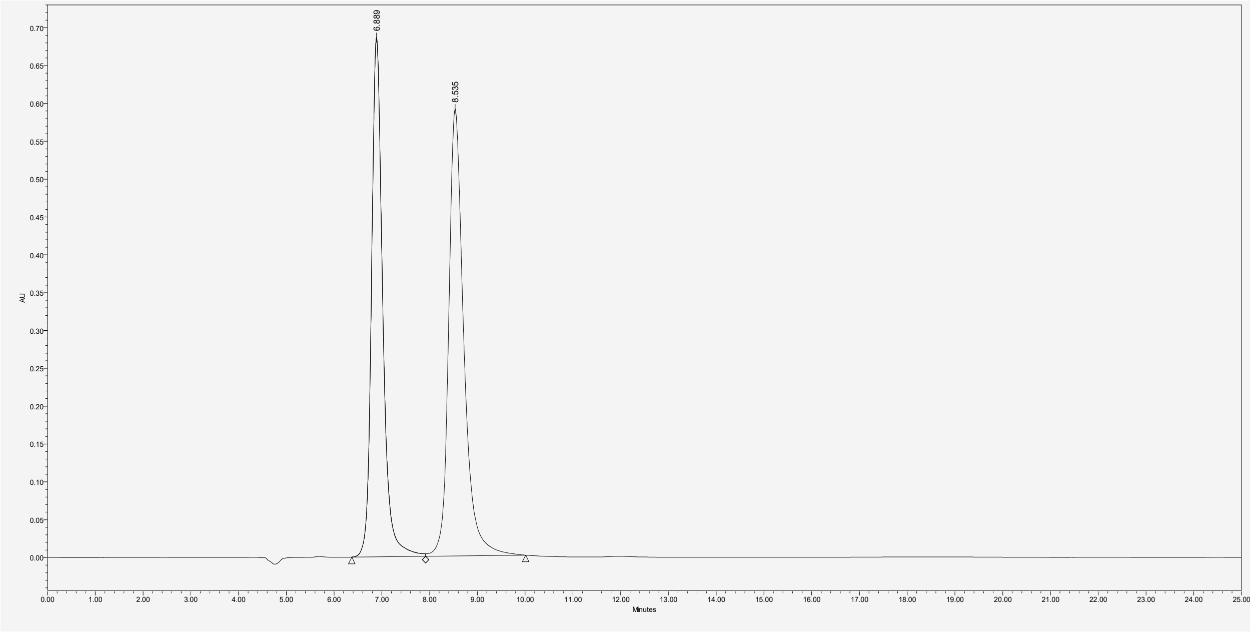

	Name	Retention Time	Area	% Area	Height
1	*(S)*-α-Me-Phe	6.889	11268935	46.34	685931
2	*(R)*-α-Me-Phe	8.535	13049528	53.66	590488

Complex № 5
ee% =10.12%

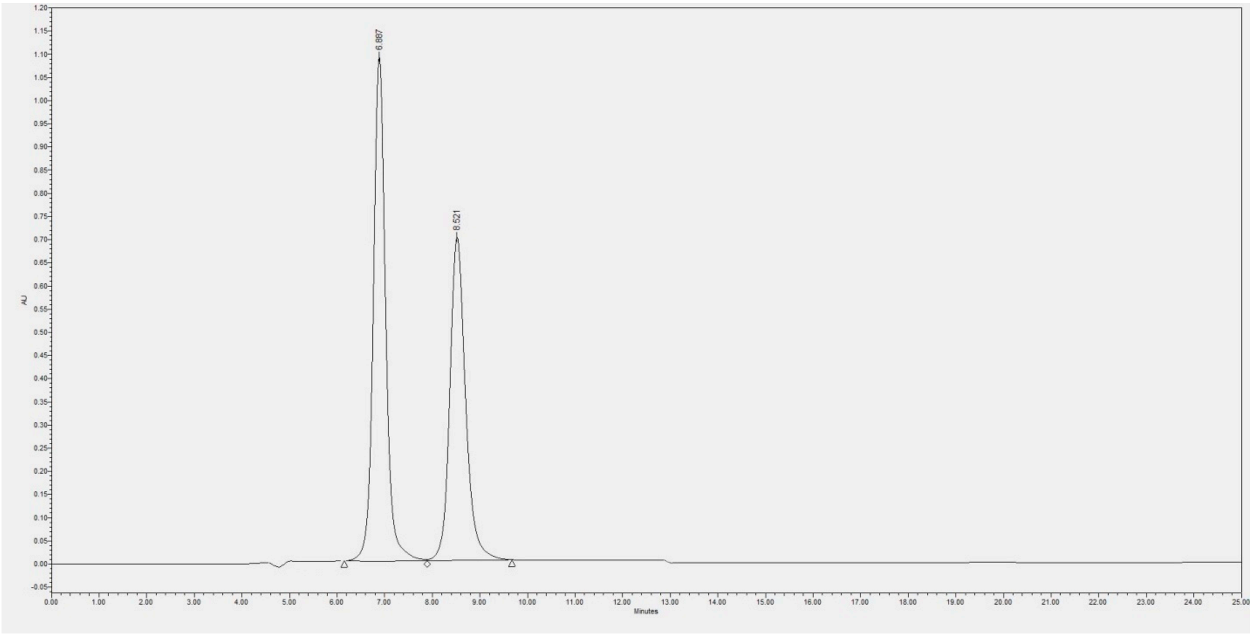

	Name	Retention Time	Area	% Area	Height
1	*(S)*-α-Me-Phe	6.887	19682500	55.06	1089126
2	*(R)*-α-Me-Phe	8.521	16063183	44.94	697692

Complex №6
ee% =7.44%

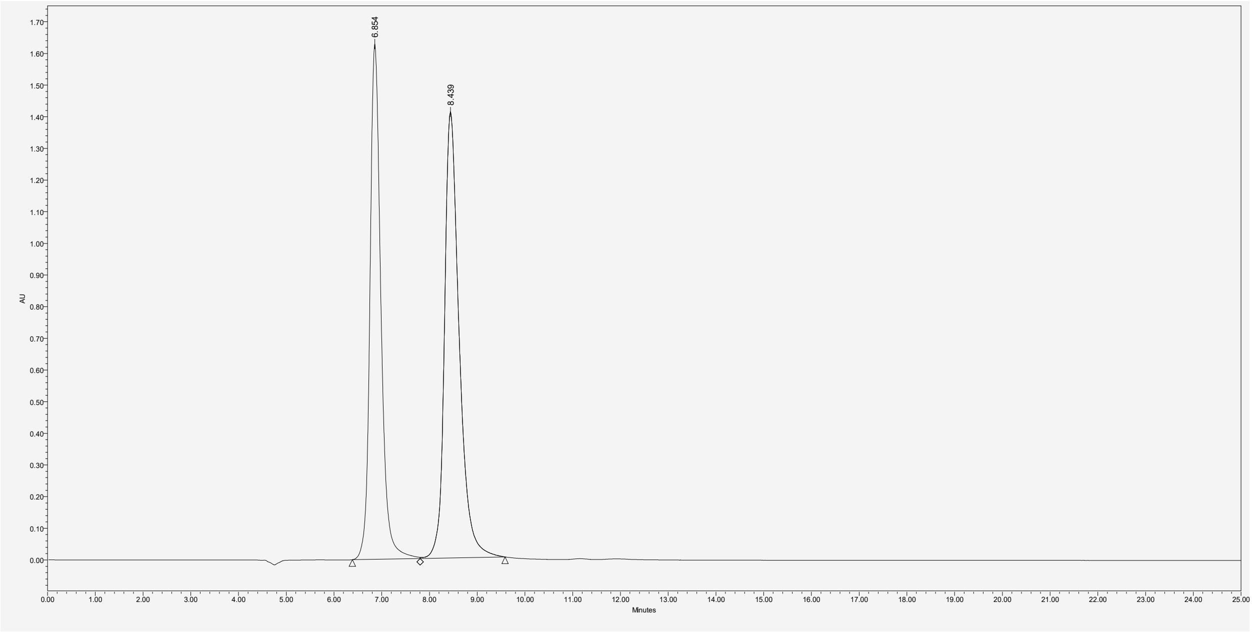

	Name	Retention Time	Area	% Area	Height
1	*(S)*-α-Me-Phe	6.854	25981714	46.28	1627817
2	*(R)*-α-Me-Phe	8.439	30158111	53.72	1408756

Complex №7
ee% =7.88%

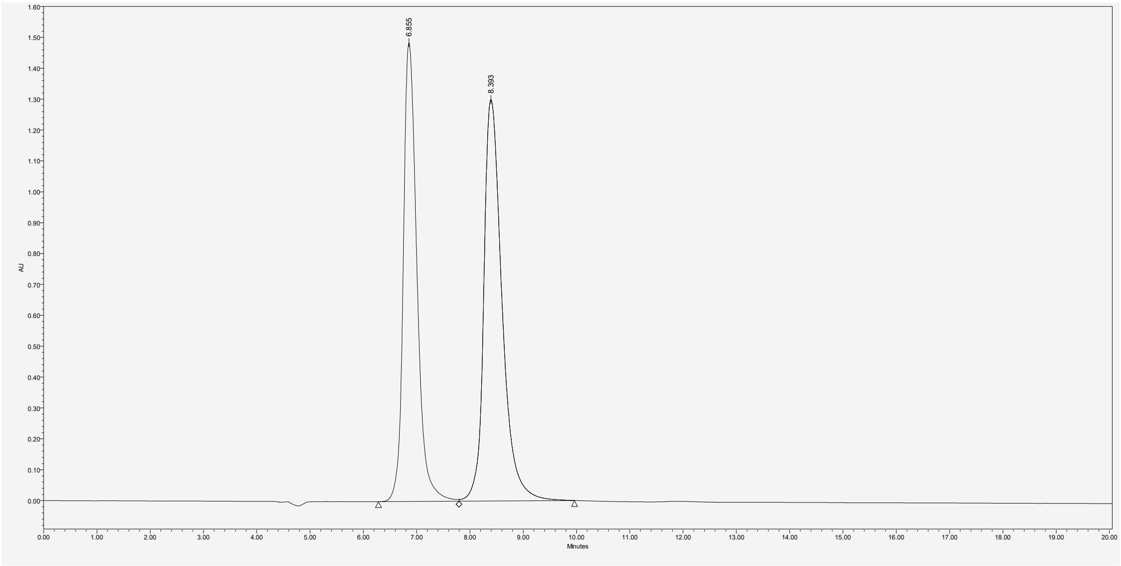

	Name	Retention Time	Area	% Area	Height
1	*(S)*-α-Me-Phe	6.855	26422121	46.06	1486165
2	*(R)*-α-Me-Phe	8.393	30944320	53.94	1300046

Complex №8
ee%=26.23%

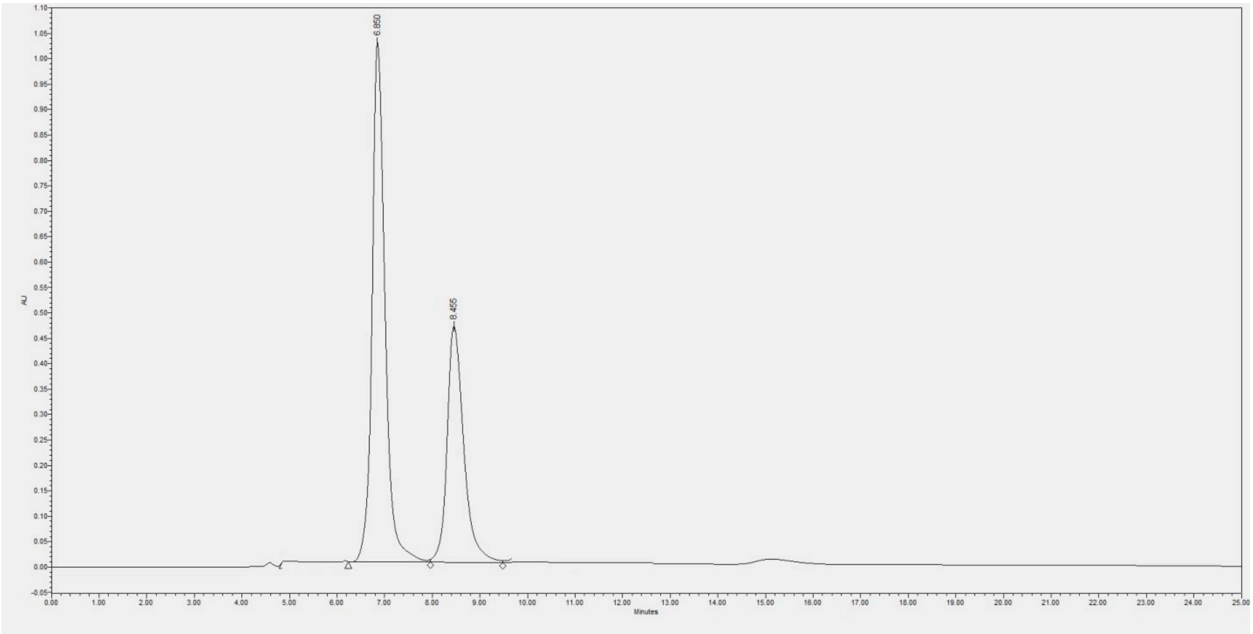

	Name	Retention Time	Area	% Area	Height
1	*(S)*-α-Me-Phe	6.850	19302711	63.12	1022351
2	*(R)*-α-Me-Phe	8.455	11280541	36.88	464206

Complex №9
ee% =10.12%

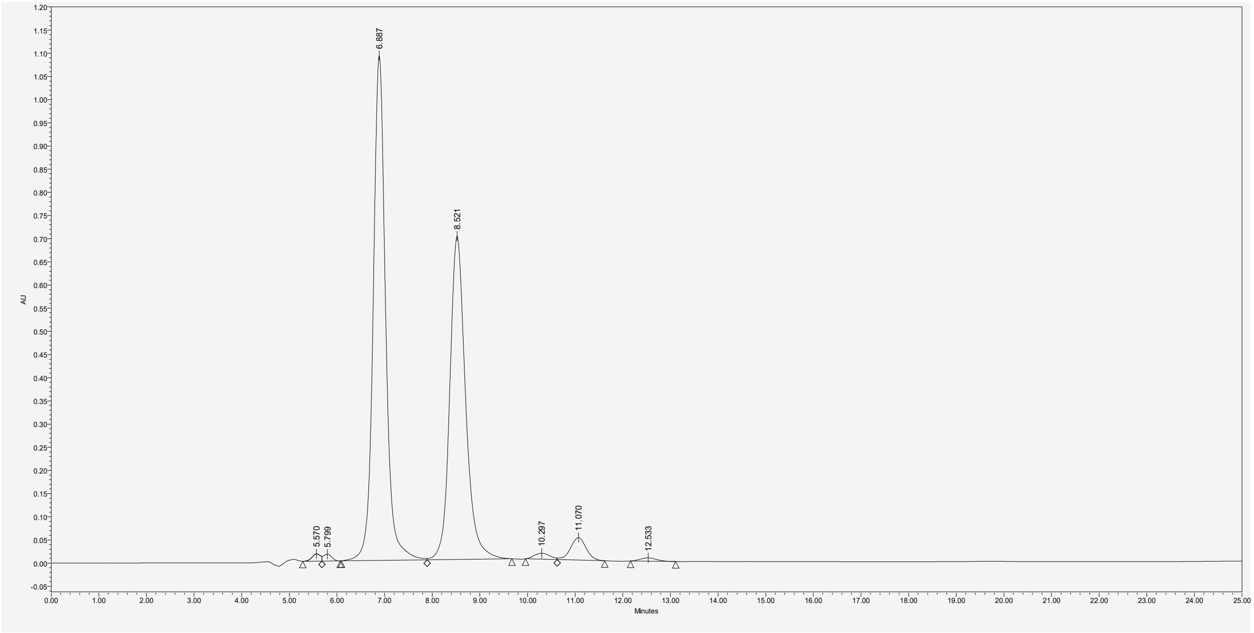

	Name	Retention Time	Area	% Area	Height
1	*(S)*-α-Me-Phe	6.887	19682500	55.06	1089126
2	*(R)*-α-Me-Phe	8.521	16063183	44.94	697692

Complex №10
ee%=3.4%

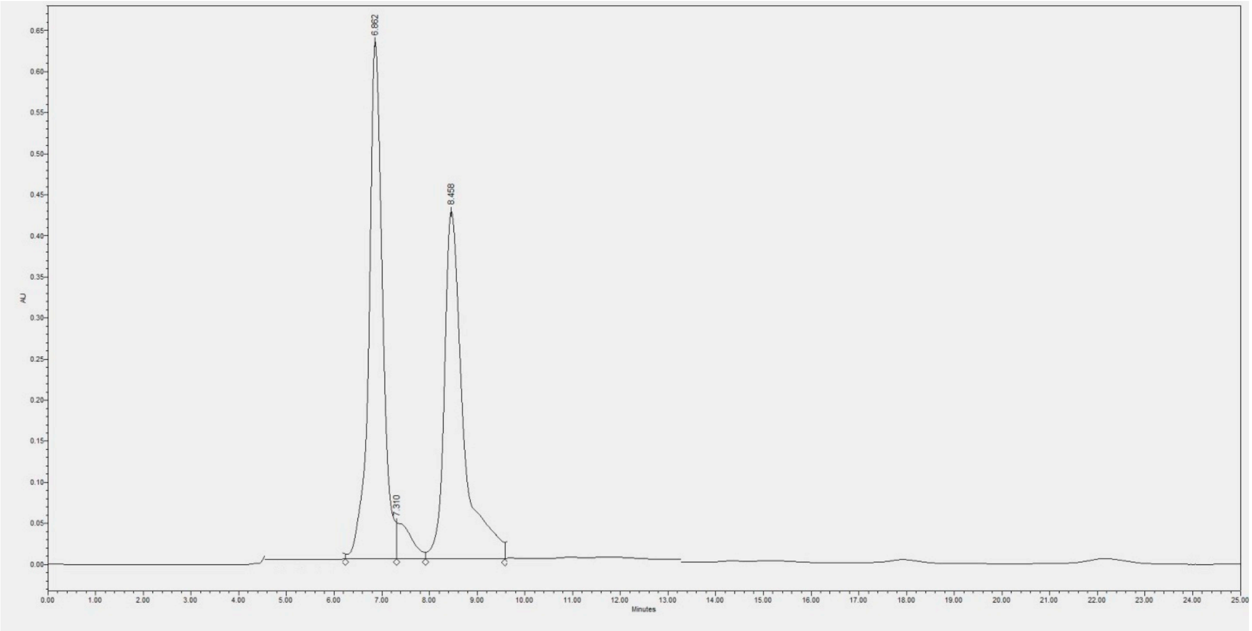

	Name	Retention Time	Area	% Area	Height
1	*(S)*-α-Me-Phe	6.862	12470441	51,7	629154
2	*(R)*-α-Me-Phe	8.458	11648794	48,3	422885

Complex №12
ee%=4.084%

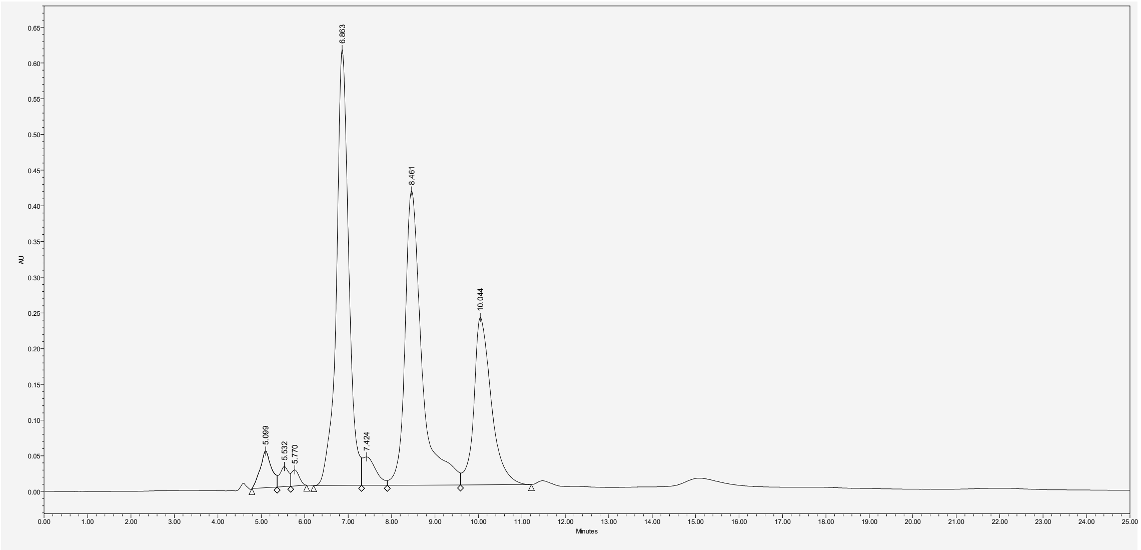

	Name	Retention Time	Area	% Area	Height
1	*(S)*-α-Me-Phe	6.863	12092512	52.04	610355
2	*(R)*-α-Me-Phe	8.461	11142518	47,96	412017

Complex №13
ee%=9.89%

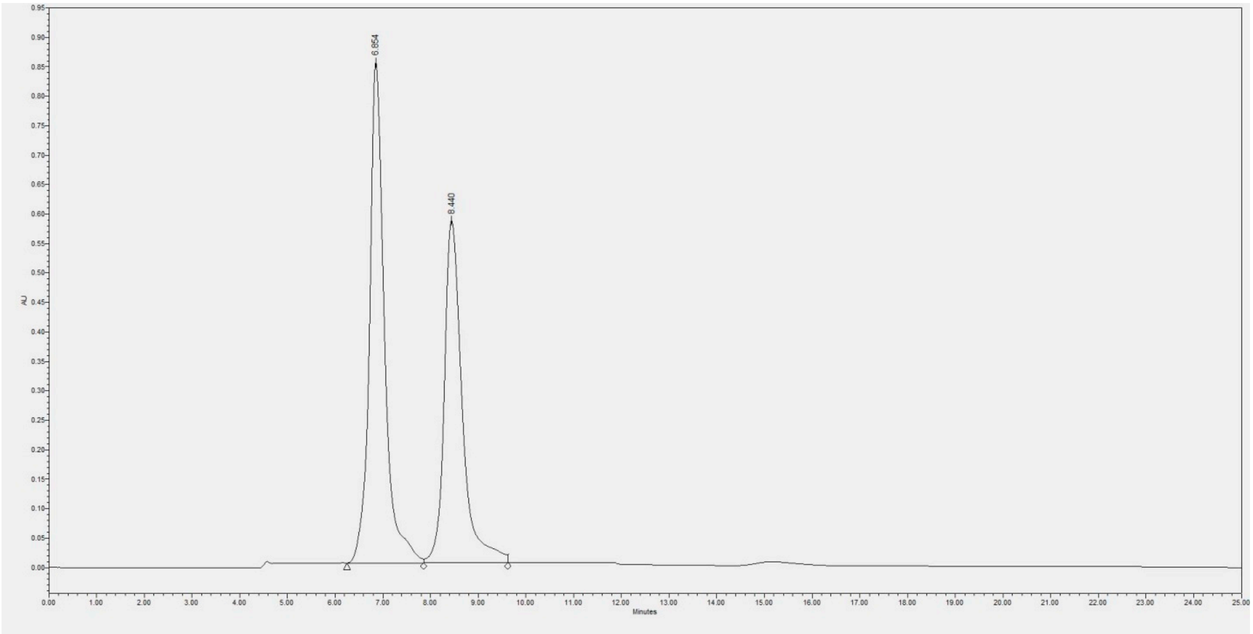

	Name	Retention Time	Area	% Area	Height
1	*(S)*-α-Me-Phe	6.854	18510104	54.95	850318
2	*(R)*-α-Me-Phe	8.440	15175671	45.05	581166

➢

S5.2. Alkylation of substrate 18

Racemate: *(S,R)*-*α*-Me-Phe

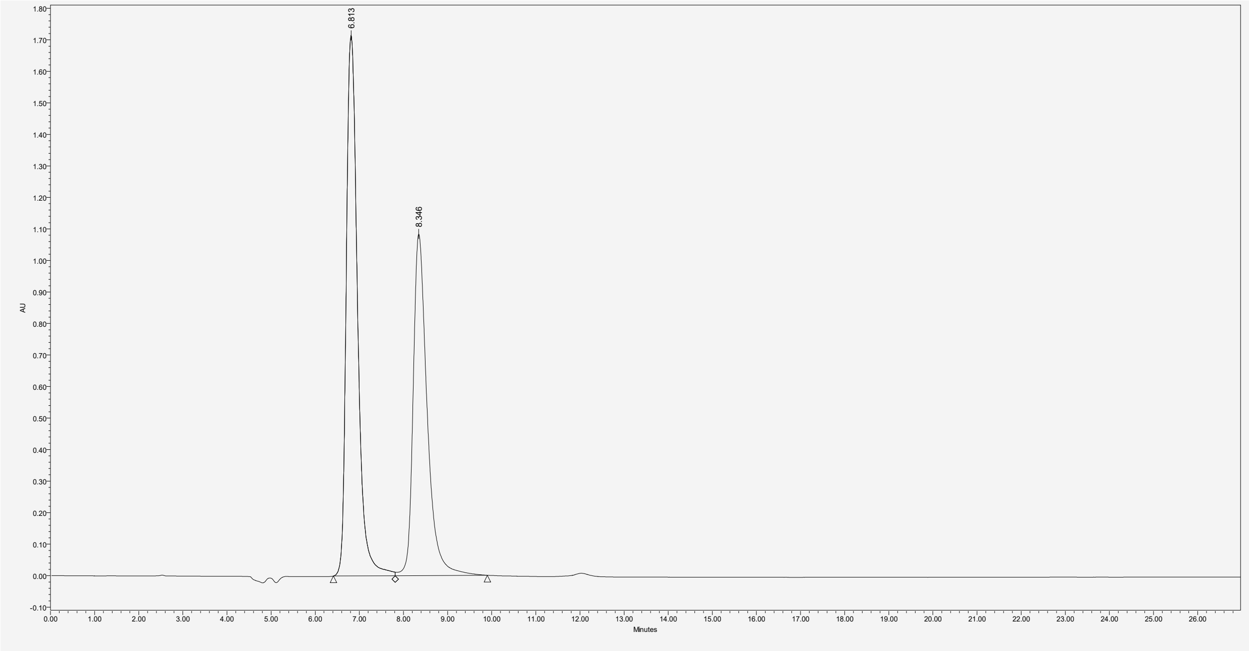

	Name	Retention Time	Area	% Area	Height
1	*(S)*-α-Me-Phe	6.813	30482230	55.98	1715154
2	*(R)*-α-Me-Phe	8.346	23972361	44.02	1083804

Complex 1
ee=70.24%

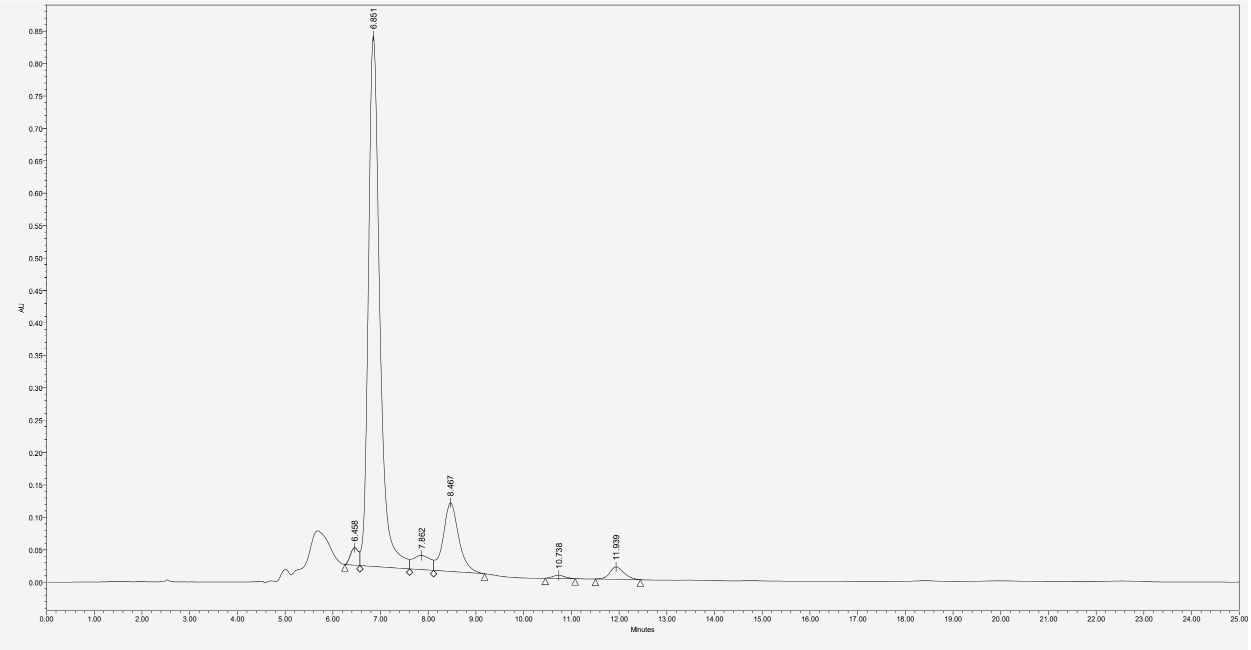

	Name	Retention Time	Area	% Area	Height
1	*(S)*-α-Me-Phe	6.851	13637557	85,12	818431
2	*(R)*-α-Me-Phe	8.467	2384444	14,88	106016

Complex 1, - 20 ^{0}C
ee= 95.78%

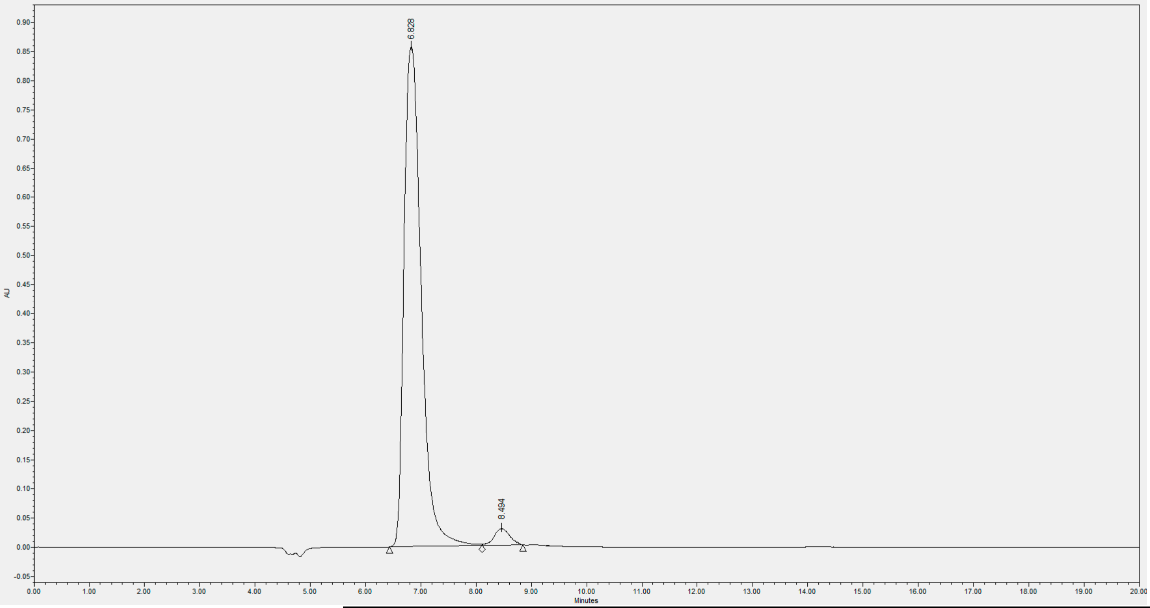

	Name	Retention Time	Area	% Area	Height
**1**	*(S)*-α-Me-Phe	6.828	20190573	97.89	918076
**2**	*(R)*-α-Me-Phe	8.494	435203.9	2.11	19788.95

Complex 2
ee=28,56%

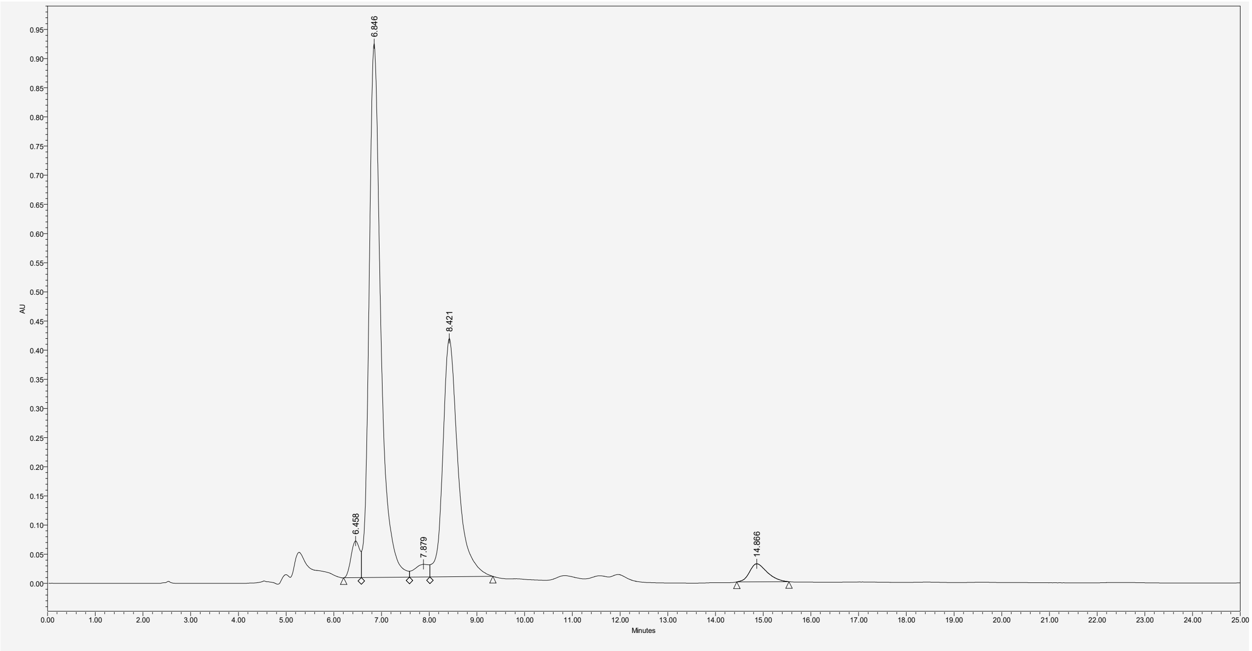

	Name	Retention Time	Area	% Area	Height
1	*(S)*-α-Me-Phe	6.846	15712130	64,28	915595
2	*(R)*-α-Me-Phe	8.421	9031322	35,72	408827

Complex 3
ee=17.95%

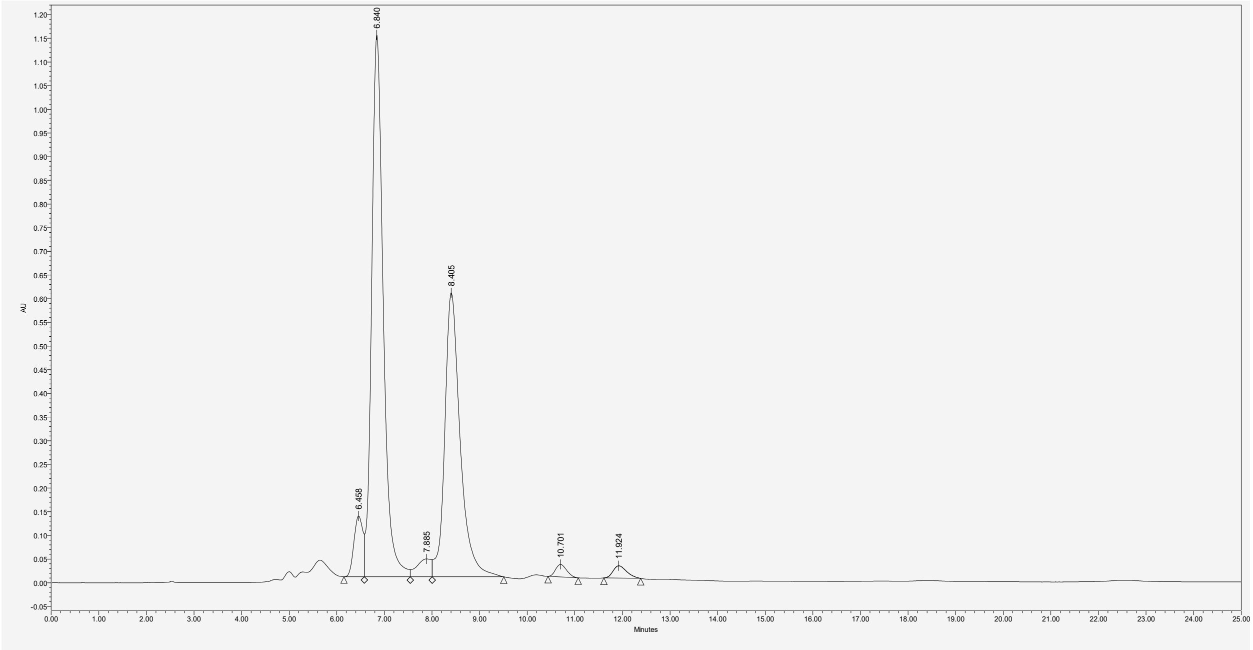

	Name	Retention Time	Area	% Area	Height
1	*(S)*-α-Me-Phe	6.840	19399461	58,98	1144420
2	*(R)*-α-Me-Phe	8.405	13494525	41,02	600092

Complex 4
ee=9.92%

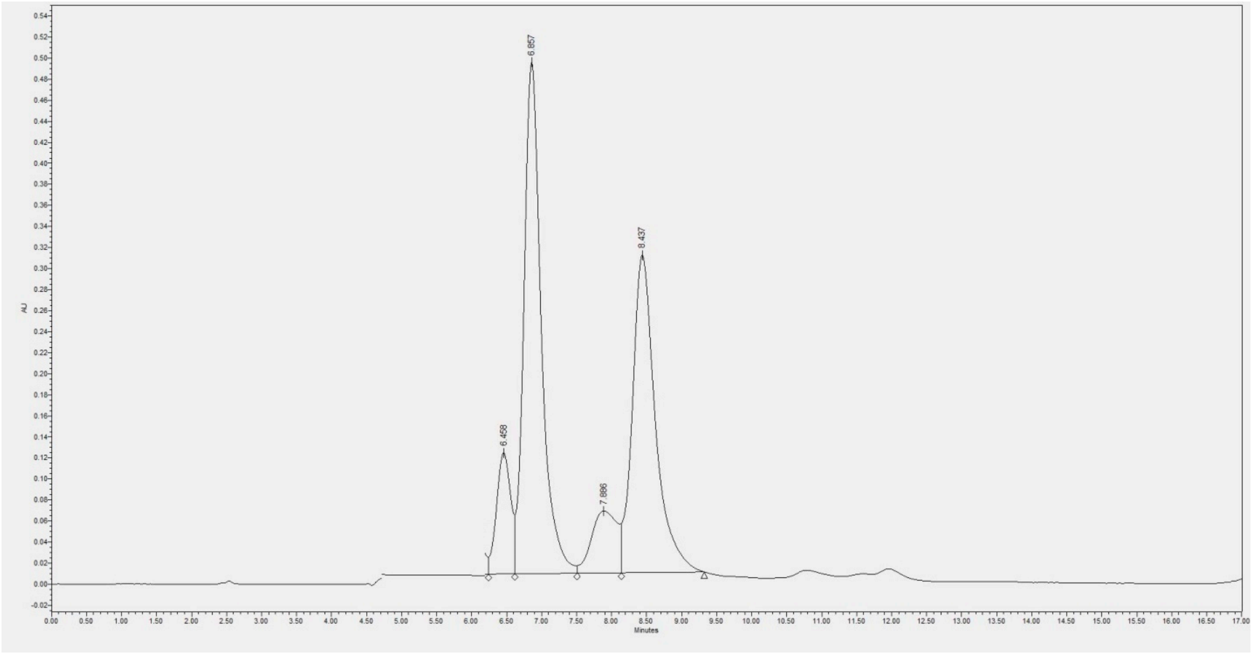

	Name	Retention Time	Area	% Area	Height
1	*(S)*-α-Me-Phe	6.857	8406000	54,96	485962
2	*(R)*-α-Me-Phe	8.437	6888563	45,04	301767

Racemate: *(S,R)*-α-Me-Phenylalanine

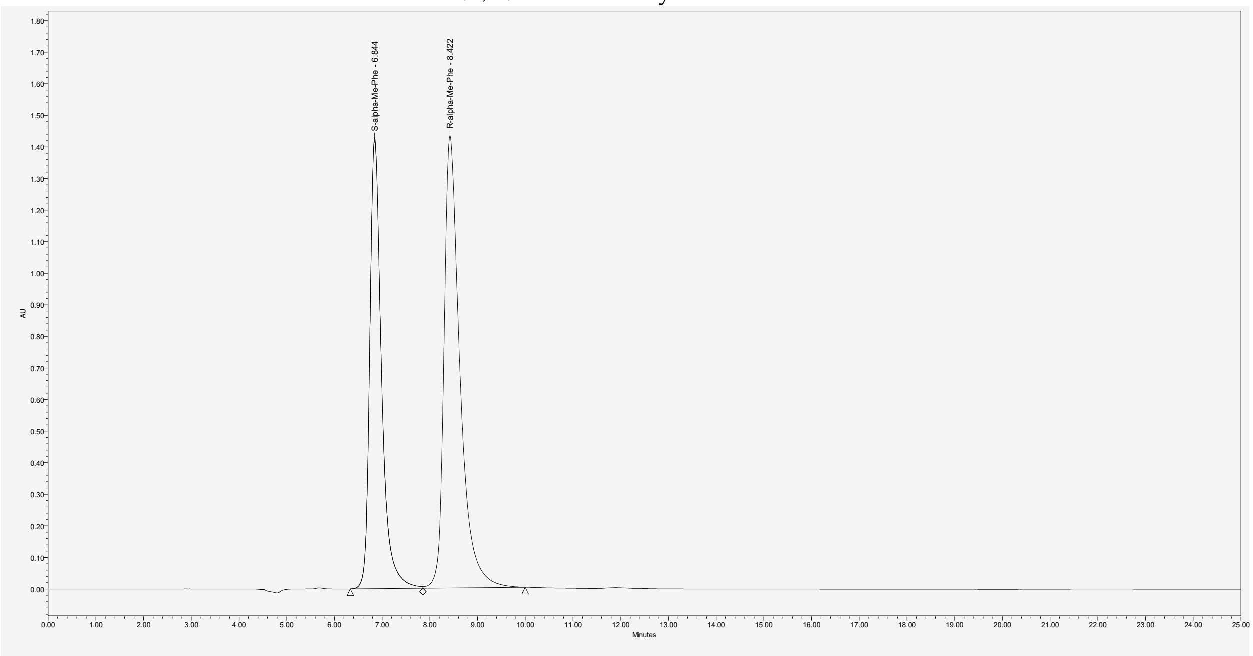

	Name	Retention Time	Area	% Area	Height
1	*(S)*-α-Me-Phe	6.844	24545524	42.45	1428332
2	*(R)*-α-Me-Phe	8.422	33270653	57.55	1432716

Complex 5
ee%=17 %

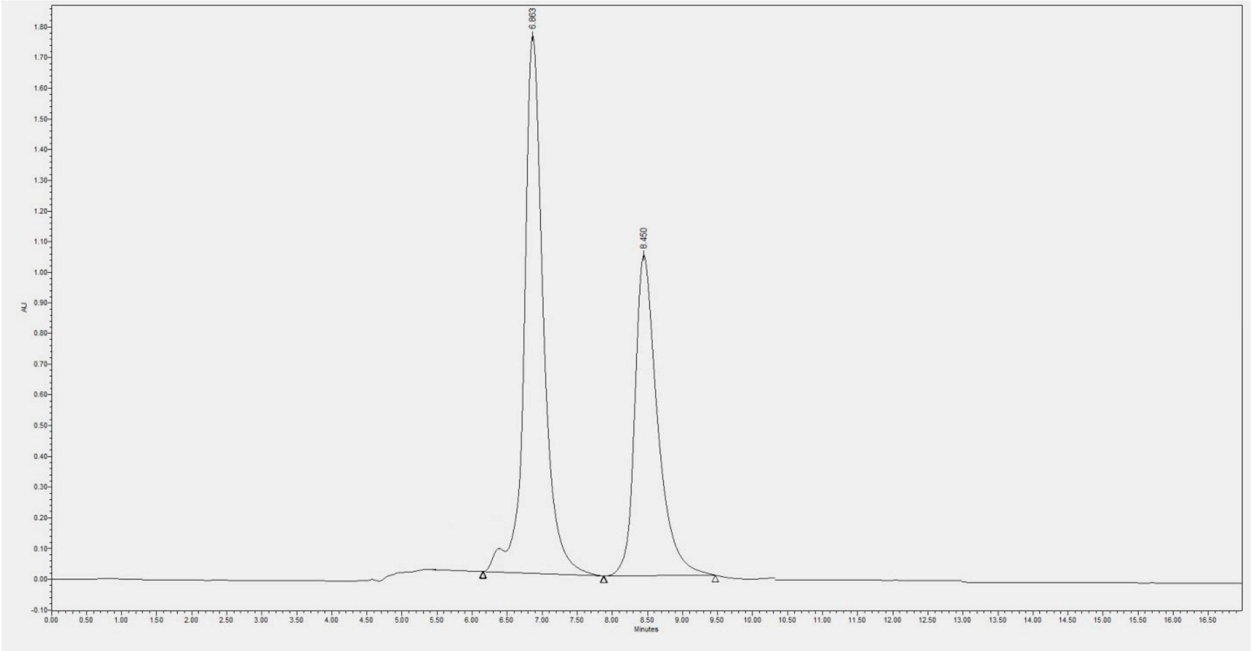

	Name	Retention Time	Area	% Area	Height
1	*(S)*-α-Me-Phe	6.863	32637263	58,14	1751291
2	*(R)*-α-Me-Phe	8.450	23500607	41,86	1043796

Complex 6
ee=15,1 %

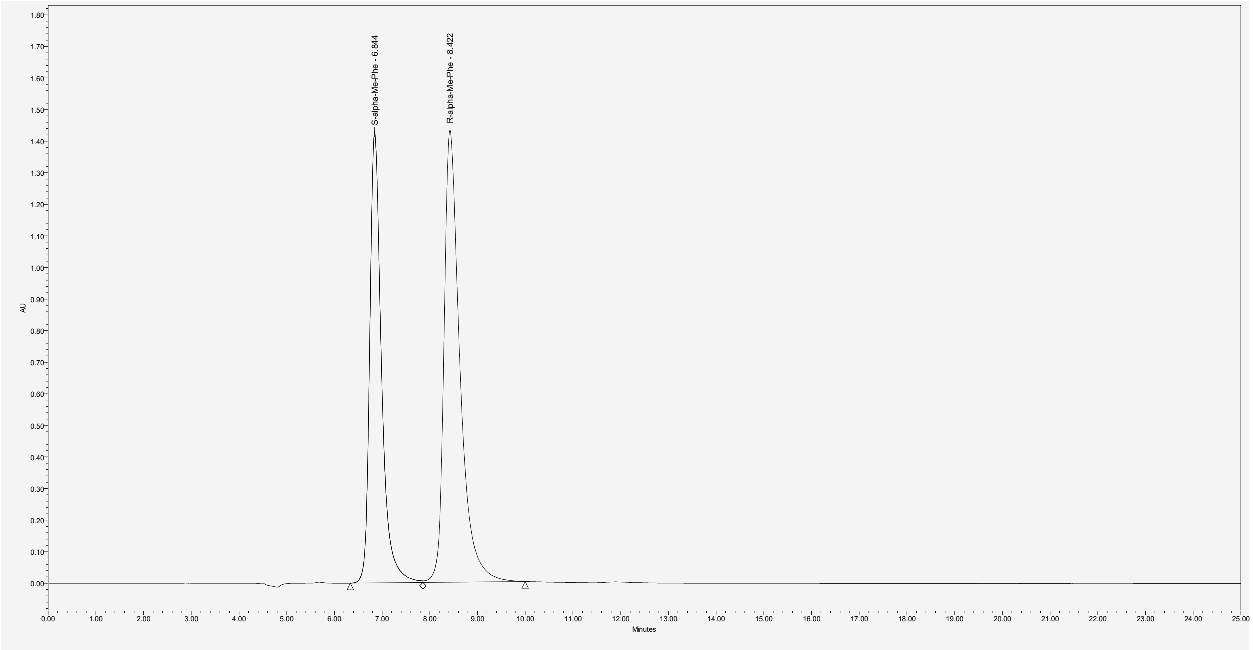

	Name	Retention Time	Area	% Area	Height
1	*(S)*-α-Me-Phe	6.844	24545524	42.45	1428332
2	*(R)*-α-Me-Phe	8.422	33270653	57.55	1432716

Complex 7
ee=14.24 %

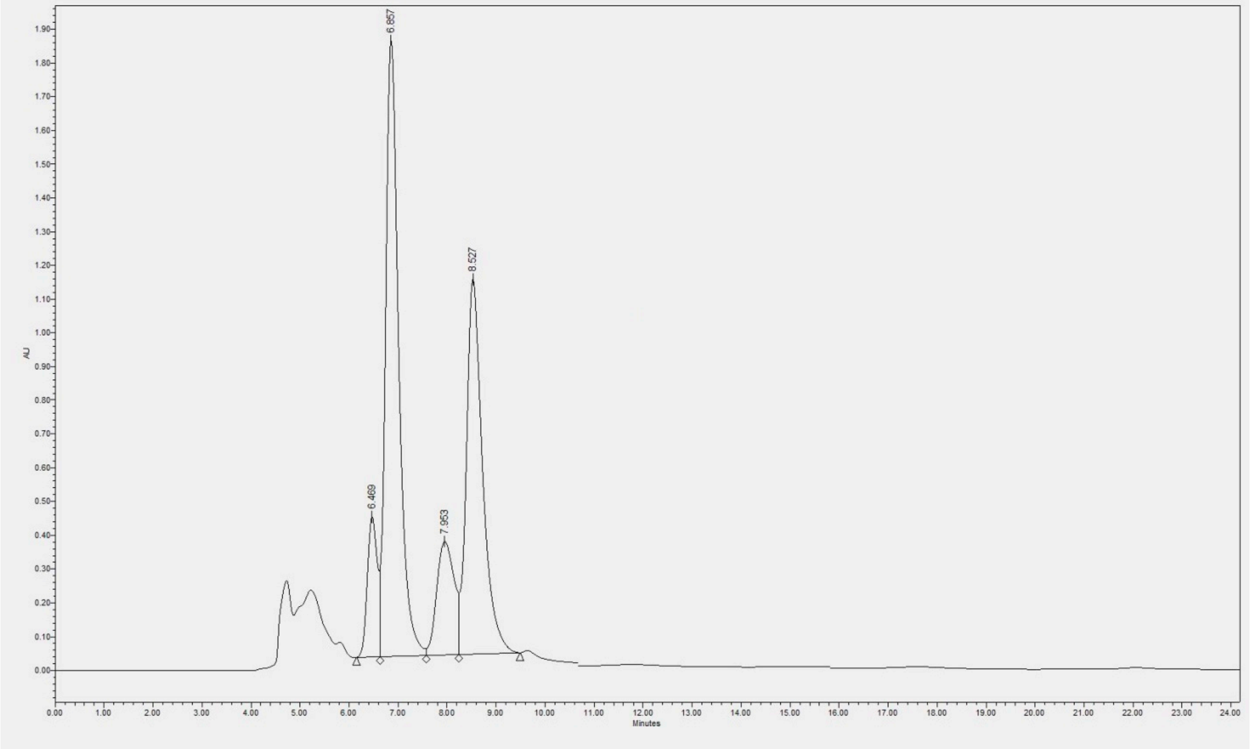

	Name	Retention Time	Area	% Area	Height
1	*(S)*-Me-Phenylalanine	6.857	33992023	57,12	1824668
2	*(R)*-Me-Phenylalanine	8.527	25518997	42,88	1111298

Complex 8
ee=37.97 %

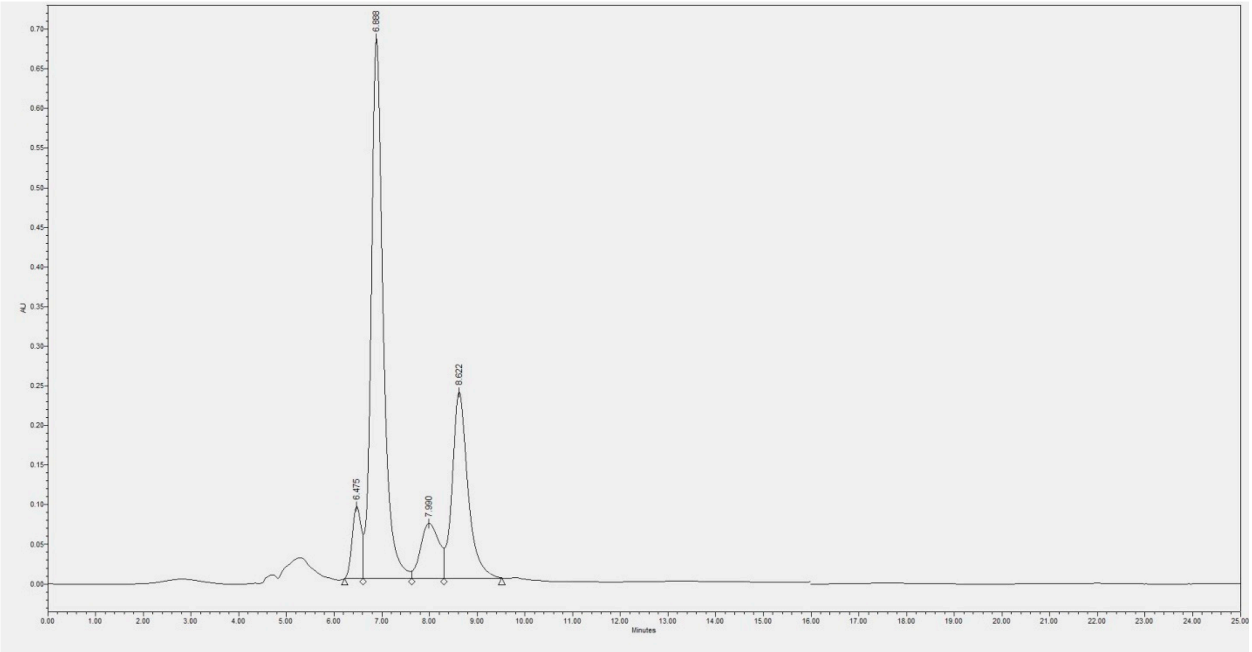

	Name	Retention Time	Area	% Area	Height
1	(S)-Me-Phenylalanine	6.888	11935059	68,99	681410
2	(R)-Me-Phenylalanine	8.622	5365729	31,01	234770

Complex 9
ee=20.15 %

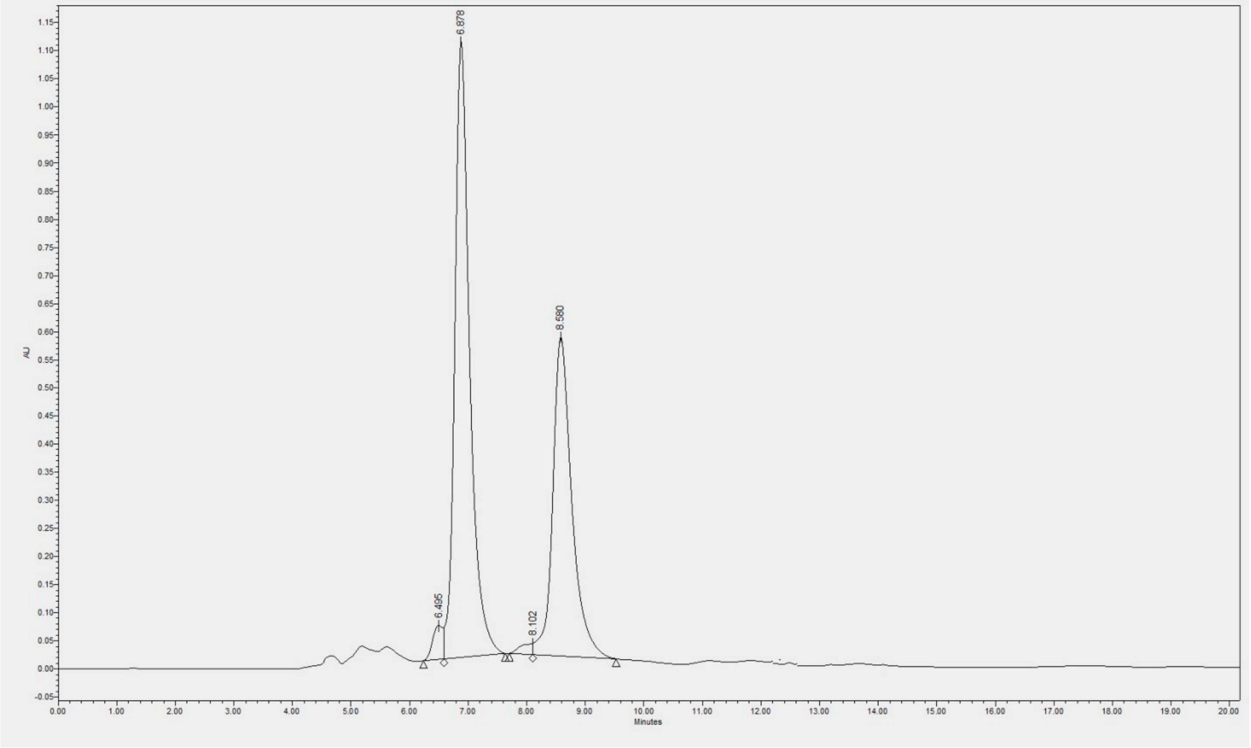

	Name	Retention Time	Area	% Area	Height
1	*(S)*-Me-Phenylalanine	6.878	19336074	60,08	1094648
2	*(R)*-Me-Phenylalanine	8.580	12849392	39,92	567323

Complex 10
ee=17,6%

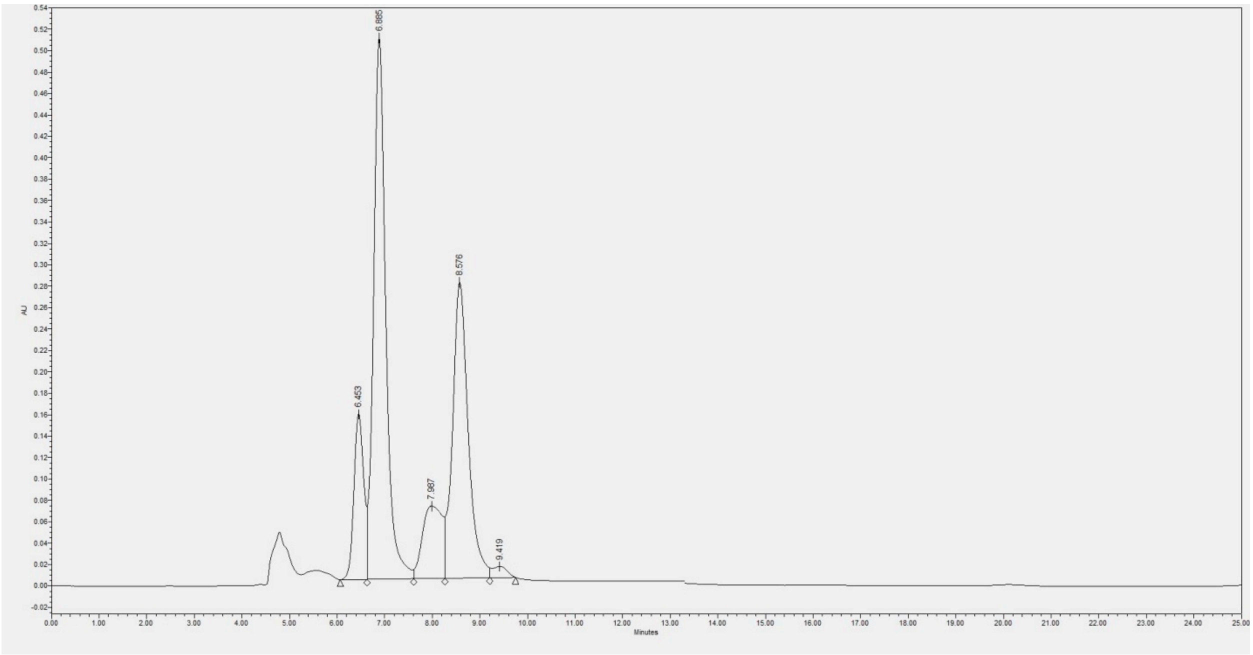

	Name	Retention Time	Area	% Area	Height
1	*(S)*-α-Me-Phe	6.885	9019781	58,80	505998
2	*(R)*-α-Me-Phe	8.576	6318581	41,20	276432

Complex 11
ee=0 %

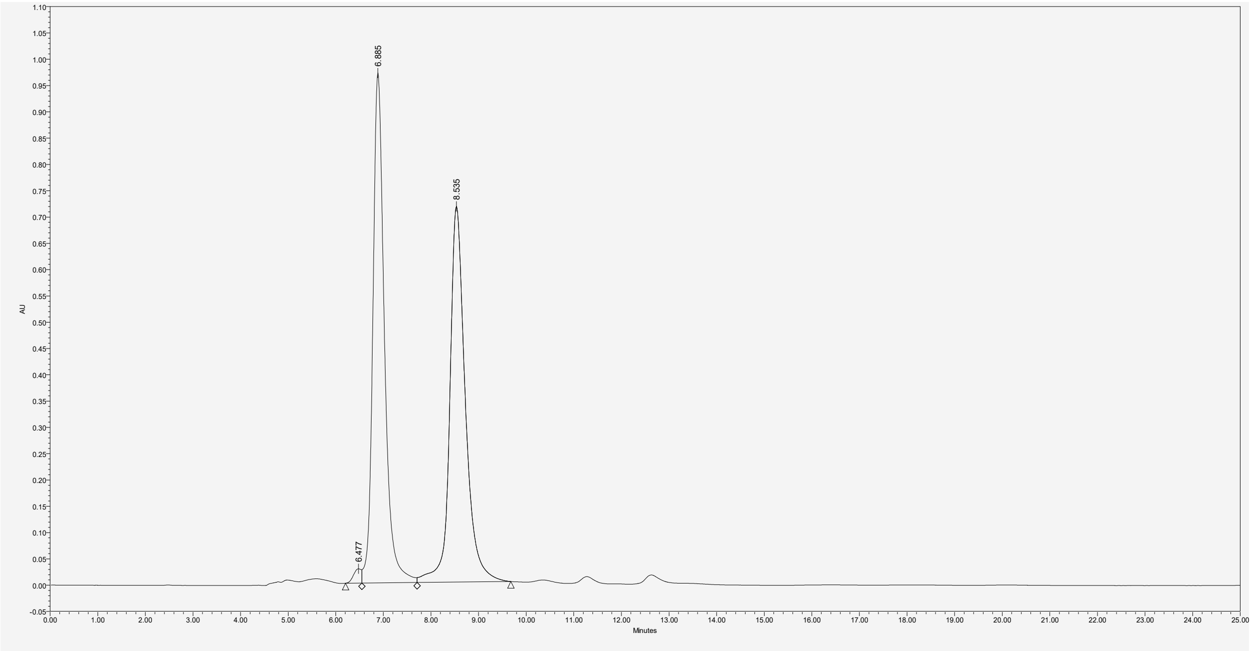

	Name	Retention Time	Area	% Area	Height
1	*(S)*-α-Me-Phe	6.885	16396136	50,20	969655
2	*(R)*-α-Me-Phe	8.535	16266103	49,80	714311

Complex 12
ee=17.52 %

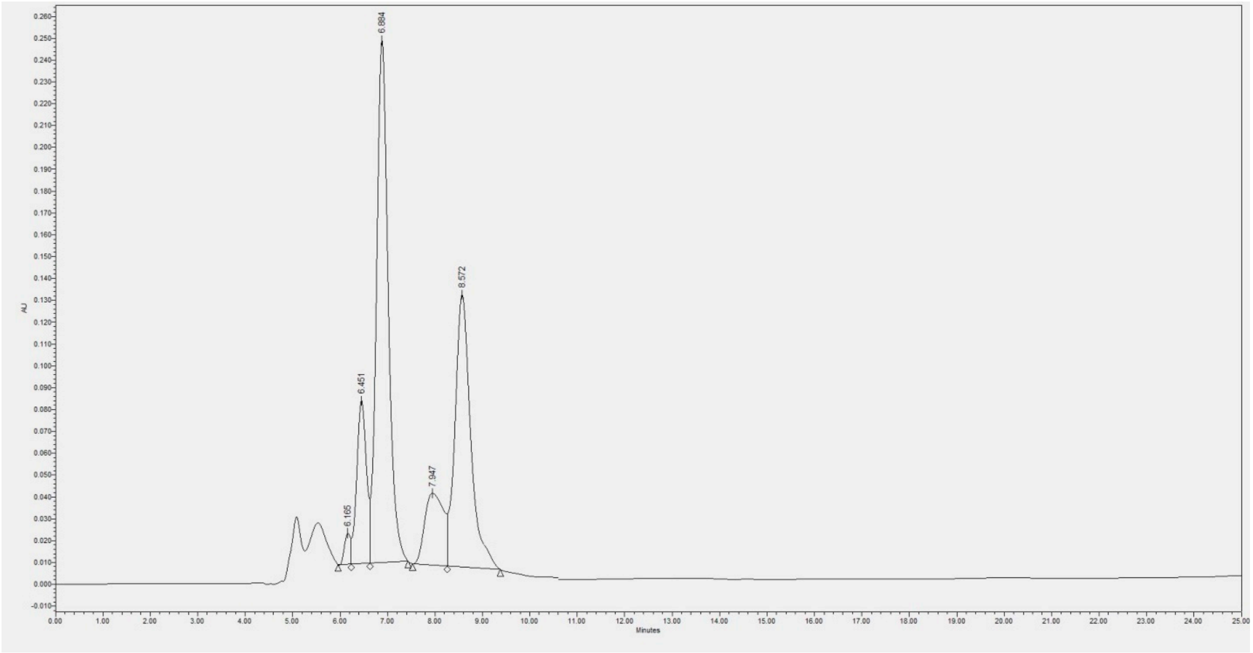

	Name	Retention Time	Area	% Area	Height
1	*(S)*-α-Me-Phe	6.884	4103149	58,76	239150
2	*(R)*-α-Me-Phe	8.572	2879591	41,24	124761

Complex 13
ee=17,6%

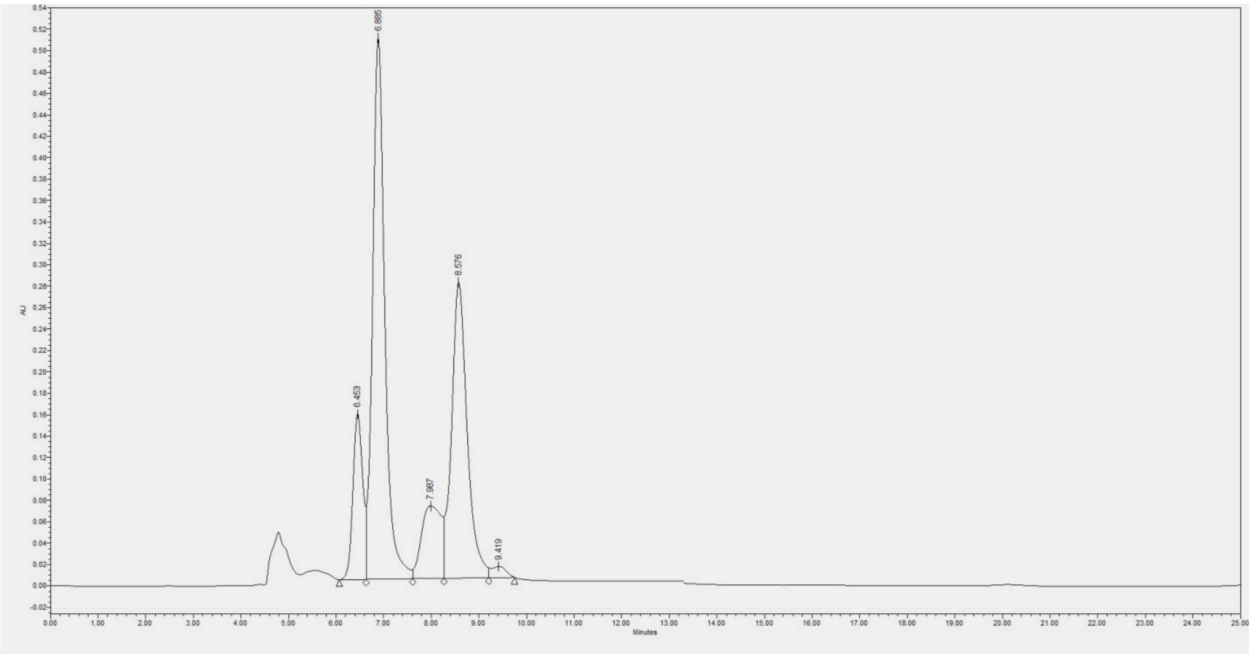

	Name	Retention Time	Area	% Area	Height
1	*(S)*-α-Me-Phe	6.885	9019781	58,80	505998
2	*(R)*-α-Me-Phe	8.576	6318581	41,20	276432

Complex 14
ee = 0

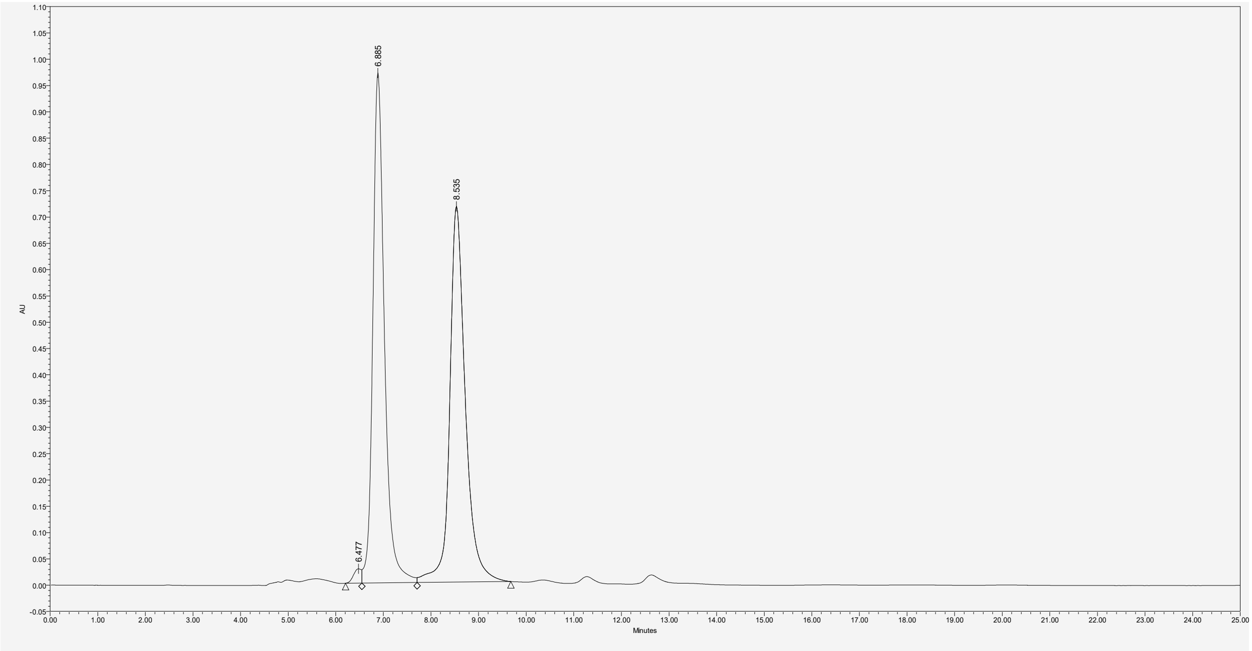

	Name	Retention Time	Area	% Area	Height
1	*(S)*-α-Me-Phe	6.885	16396136	50,20	969655
2	*(R)*-α-Me-Phe	8.535	16266103	49,80	714311

➢

S5.3. Alkylation of amino acid derivative 19

Racemte *(S,R)*-Me-Phenylalanine

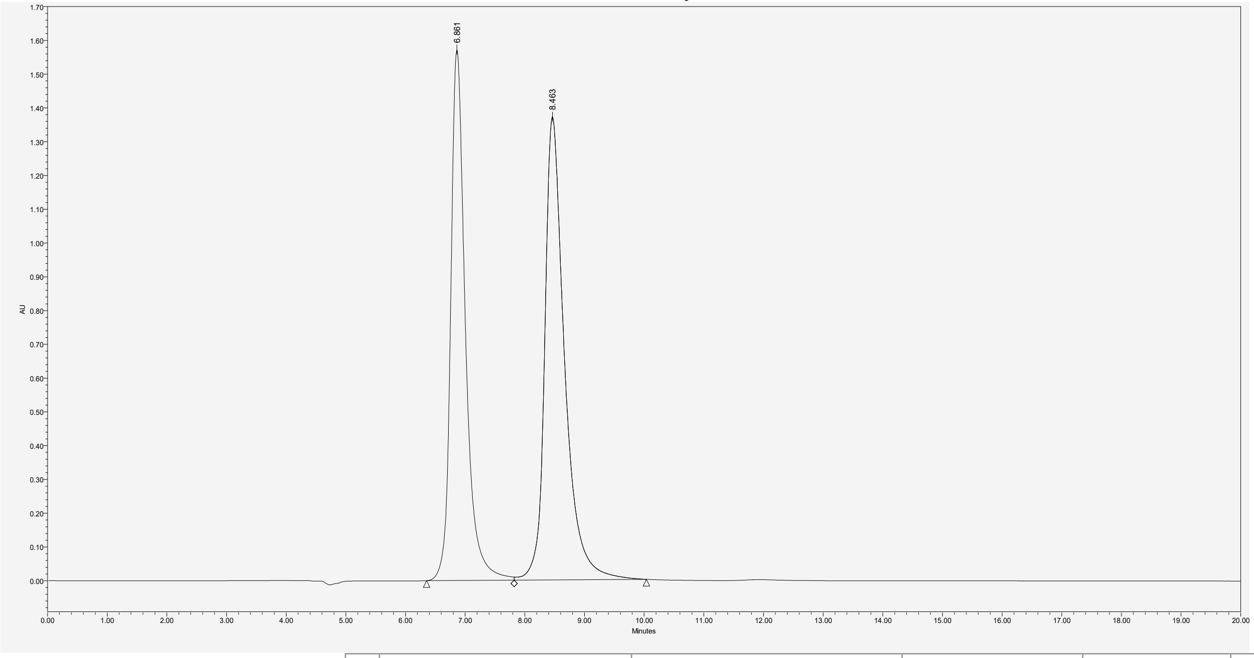

	Name	Retention Time	Area	% Area	Height
1	*(S)*-α-Me-Phe	6.861	27060139	46.01	1571897
2	*(R)*-α-Me-Phe	8.463	31756877	53.99	1371294

Complex 1
ee% =78.24%

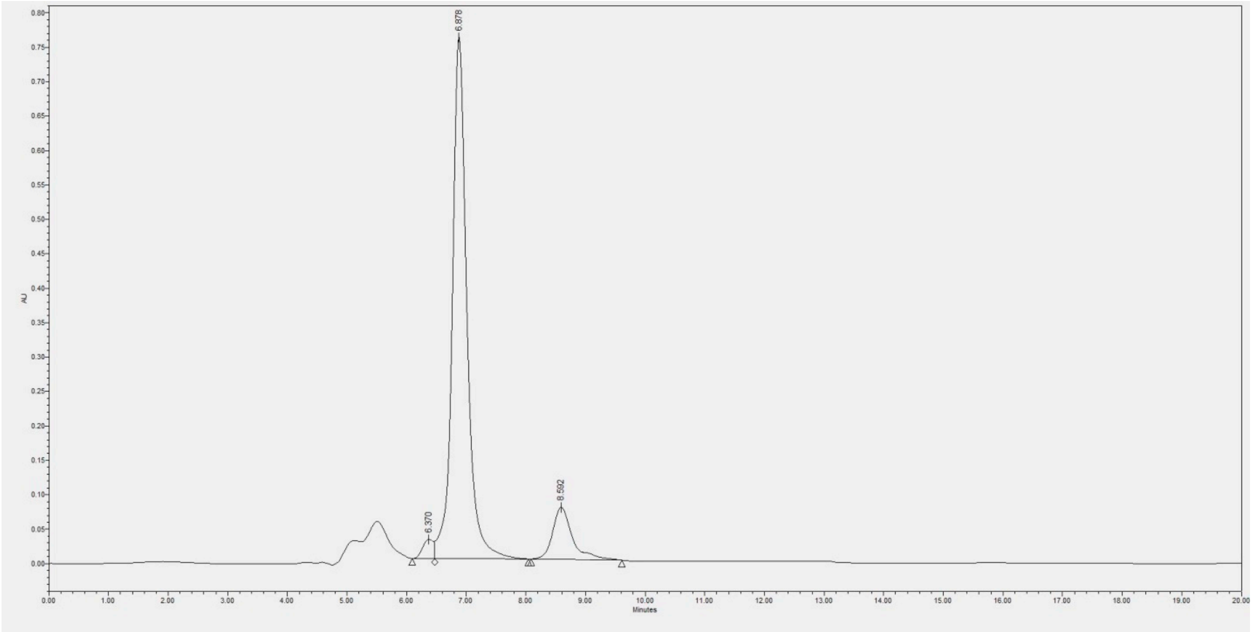

	Name	Retention Time	Area	% Area	Height
1	*(S)*-α-Me-Phe	6.878	12862515	89.12	757392
2	*(R)*-α-Me-Phe	8.592	1569871	10.88	75642

Complex 1

ee% = 98.38 %, - 20 ^{0}C

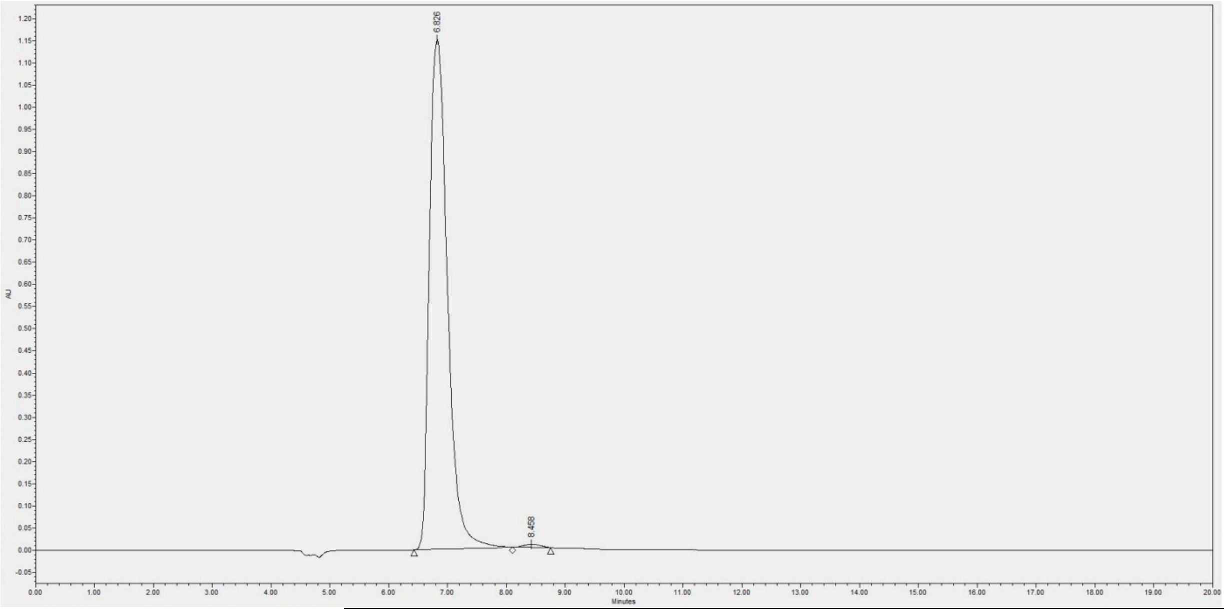

	Name	Retention Time	Area	% Area	Height
**1**	*(S)*-α-Me-Phe	6.826	26692610	99.19	1234755
**2**	*(R)*-α-Me-Phe	8.458	217975.7	0.81	10083.19

Complex 2
ee%=27.67%

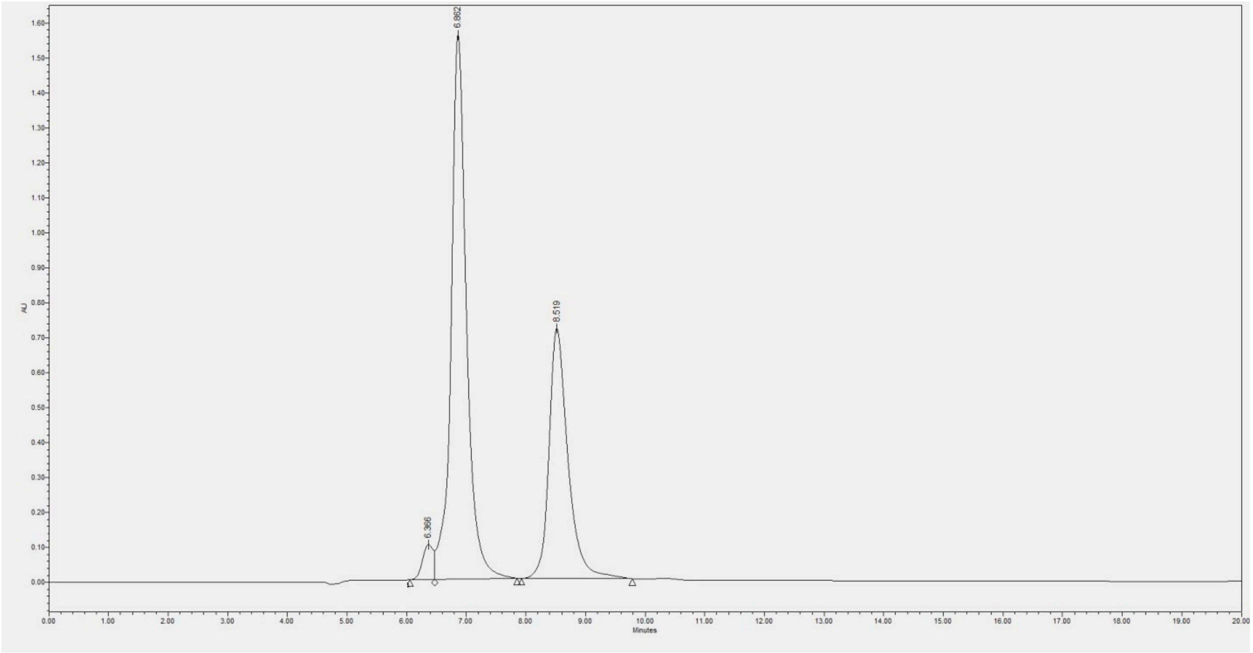

	Name	Retention Time	Area	% Area	Height
1	*(S)*-α-Me-Phe	6.862	27875056	63,84	1556662
2	*(R)*-α-Me-Phe	8.519	15791558	36,16	714977

Complex 5
ee% =30.36%

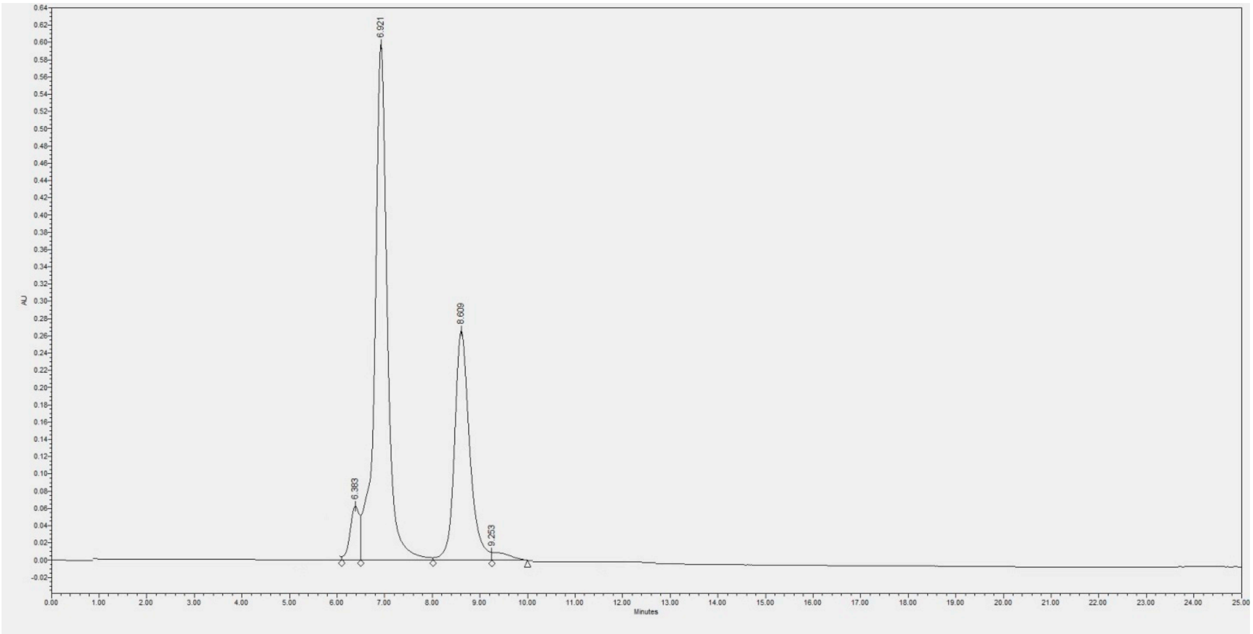

	Name	Retention Time	Area	% Area	Height
1	*(S)*-α-Me-Phe	6.921	10942870	65,18	597932
2	*(R)*-α-Me-Phe	8.609	5844929	34,82	265587

Complex 6
ee% =21.52%

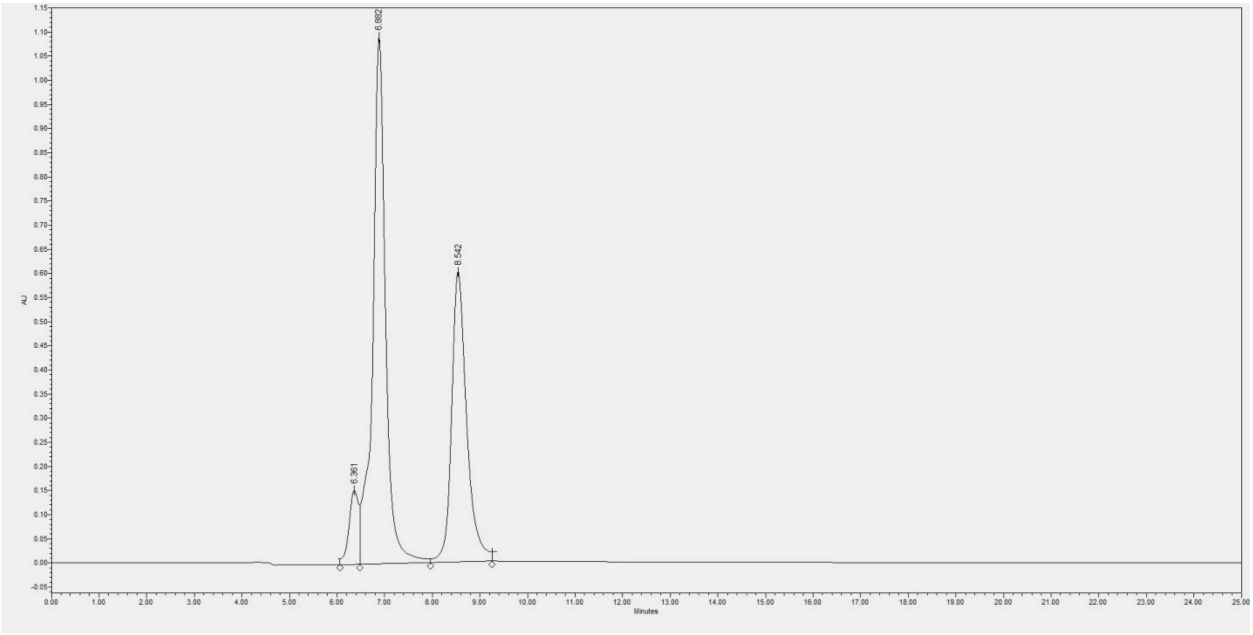

	Name	Retention Time	Area	% Area	Height
1	*(S)*-α-Me-Phe	6.882	20474059	60,76	1091780
2	*(R)*-α-Me-Phe	8.542	13220358	39,24	600936

Complex 8
ee% =42.88%

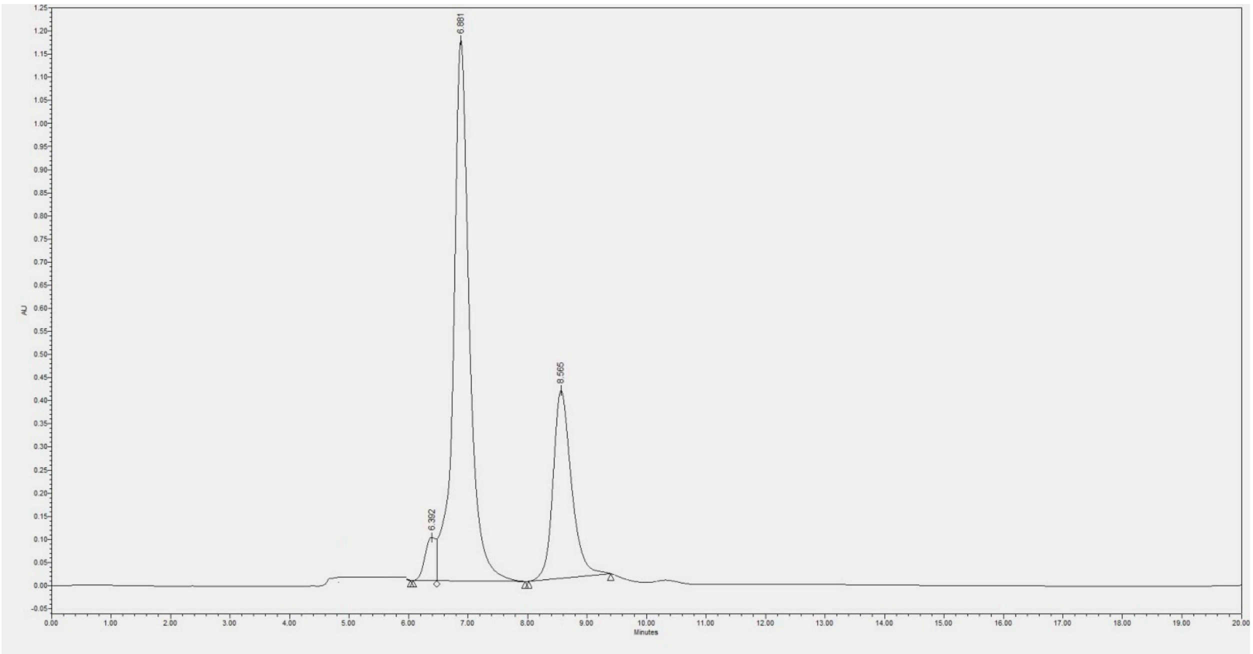

	Name	Retention Time	Area	% Area	Height
1	*(S)*-α-Me-Phe	6.881	21845807	71.44	1170263
2	*(R)*-α-Me-Phe	8.565	8732603	28,56	406914

Complex 8, - 20 ⁰C
ee% =79.78%

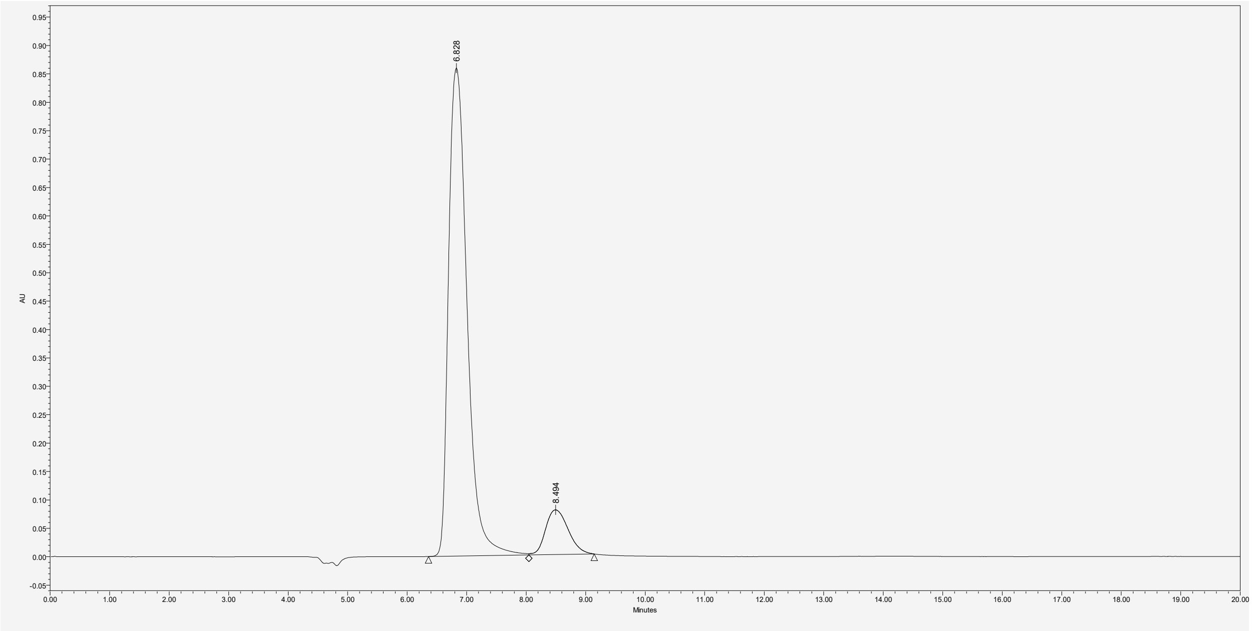

	Name	Retention Time	Area	% Area	Height
1	*(S)*-α-Me-Phe	6.828	18541492	89.89	859184
2	*(R)*-α-Me-Phe	8.494	2084285	10.11	78681

Complex 9
ee%=16.28%

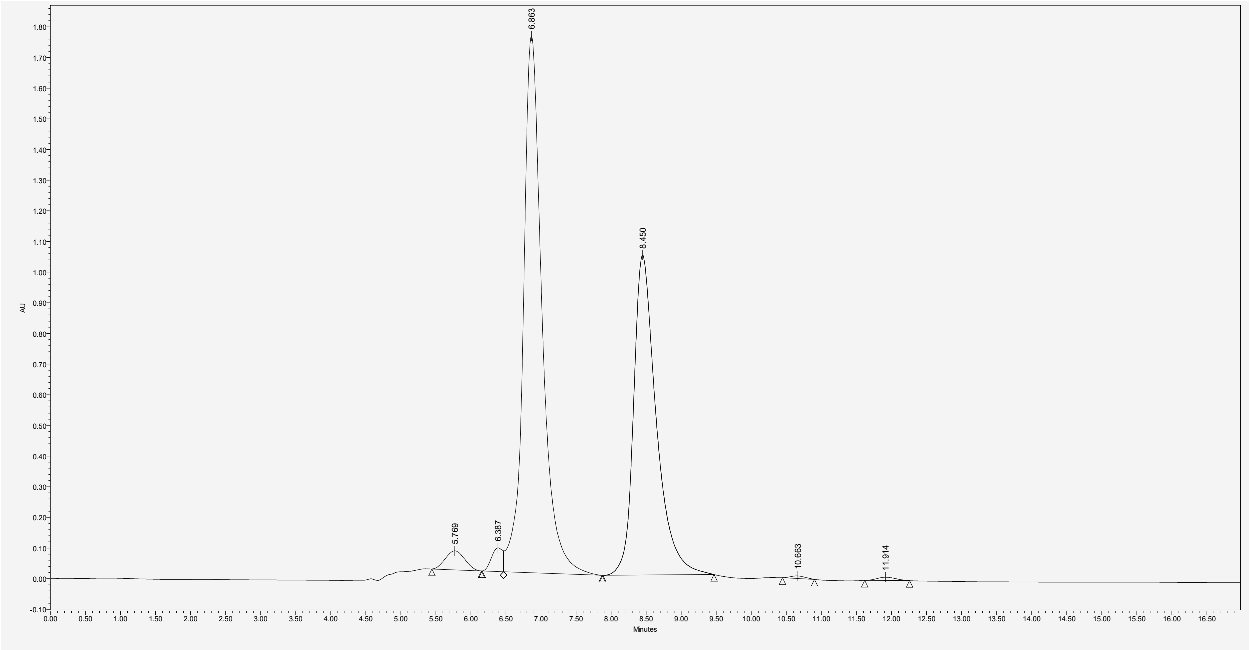

	Name	Retention Time	Area	% Area	Height
1	*(S)*-α-Me-Phe	6.863	32637263	58,14	1751291
2	*(R)*-α-Me-Phe	8.450	23500607	41,86	1043796

Complex 12
ee%=15.6%

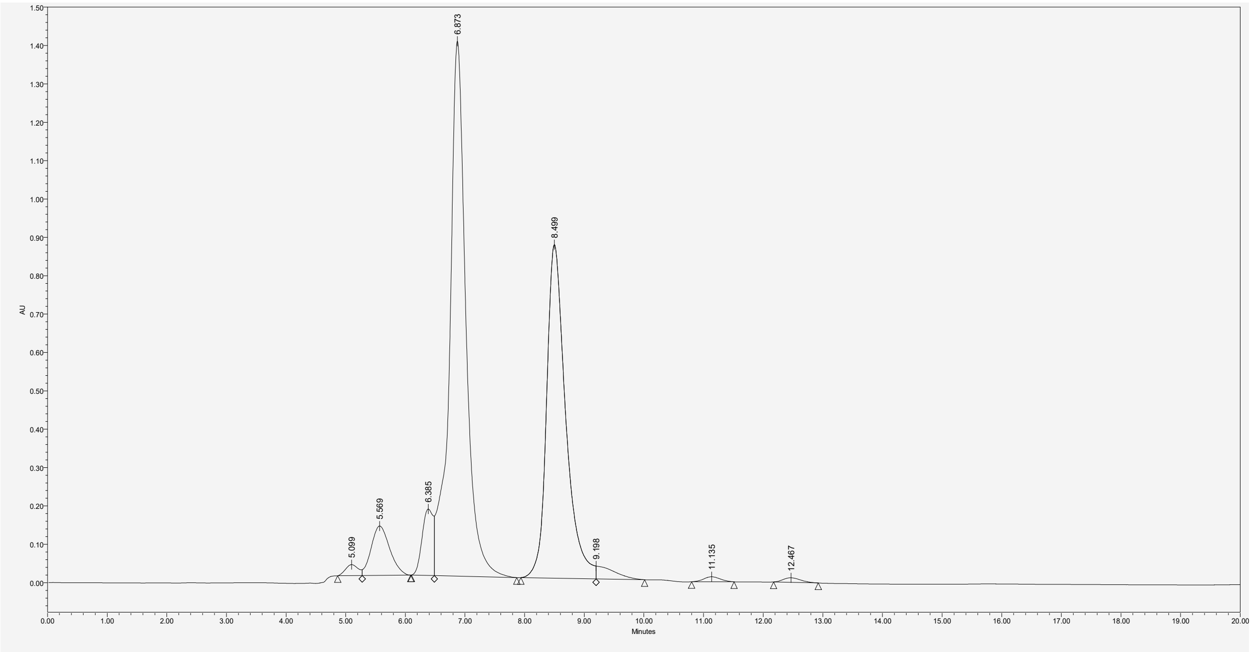

	Name	Retention Time	Area	% Area	Height
1	*(S)*-α-Me-Phe	6.873	26564985	57,79	1394126
2	*(R)*-α-Me-Phe	8.499	19404967	42,21	869342

Complex 13
ee% =10.97%

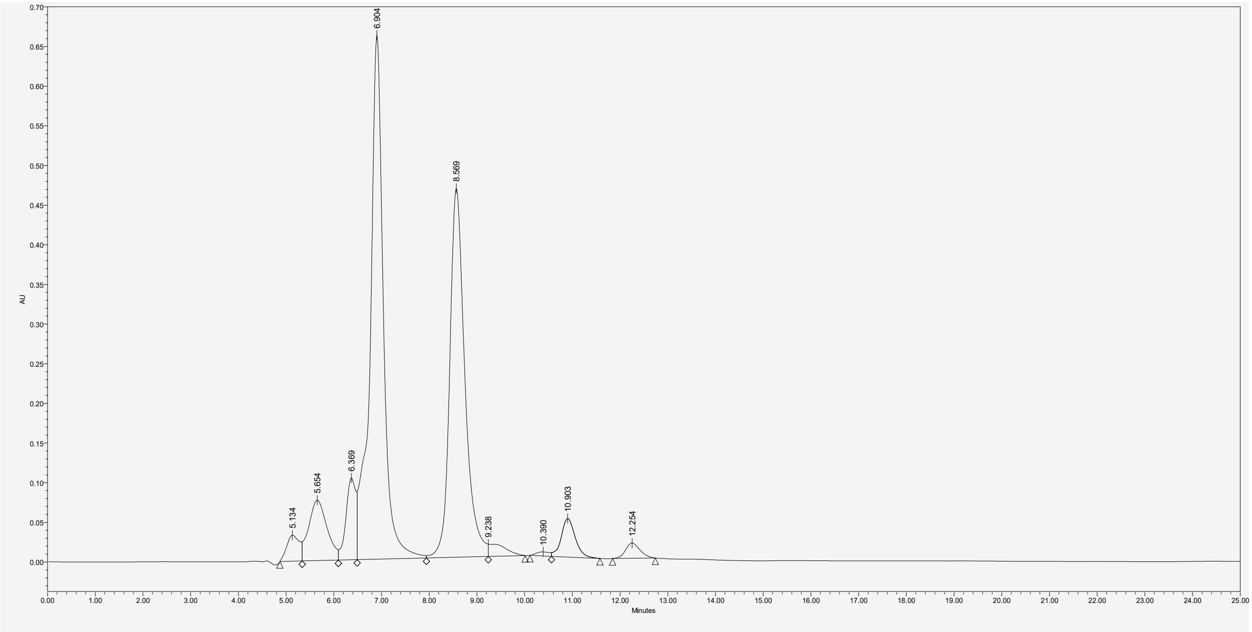

	Name	Retention Time	Area	% Area	Height
1	*(S)*-α-Me-Phe	6.904	12803736	55,49	660485
2	*(R)*-α-Me-Phe	8.569	10270748	44,51	464922

S6. FT-IR spectroscopy

Compound 1

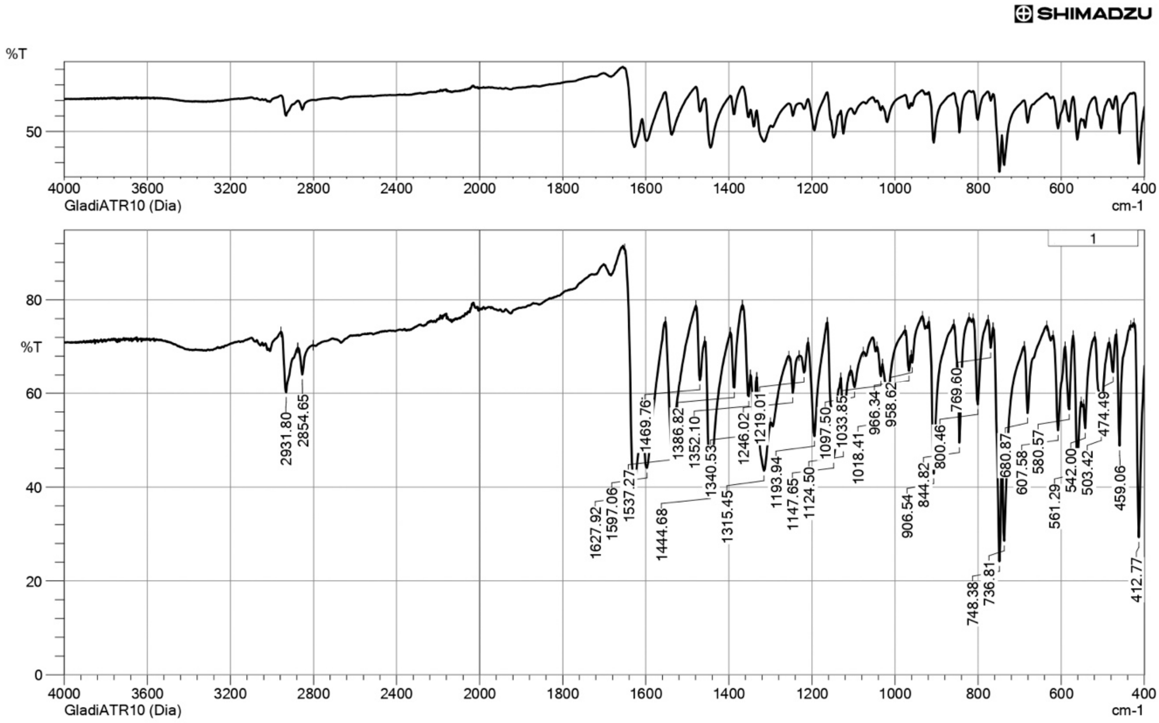

Compound 2

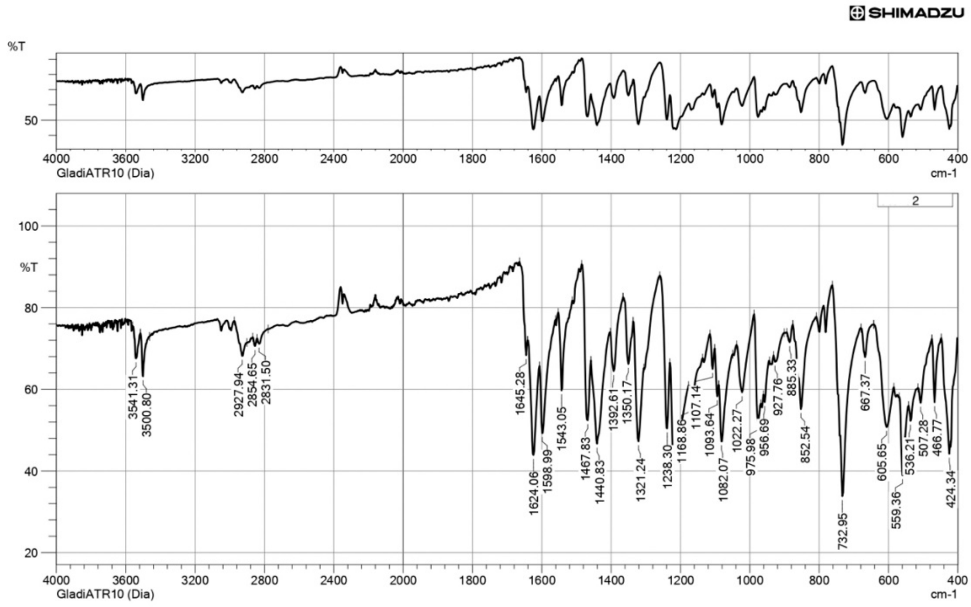

Compound 3

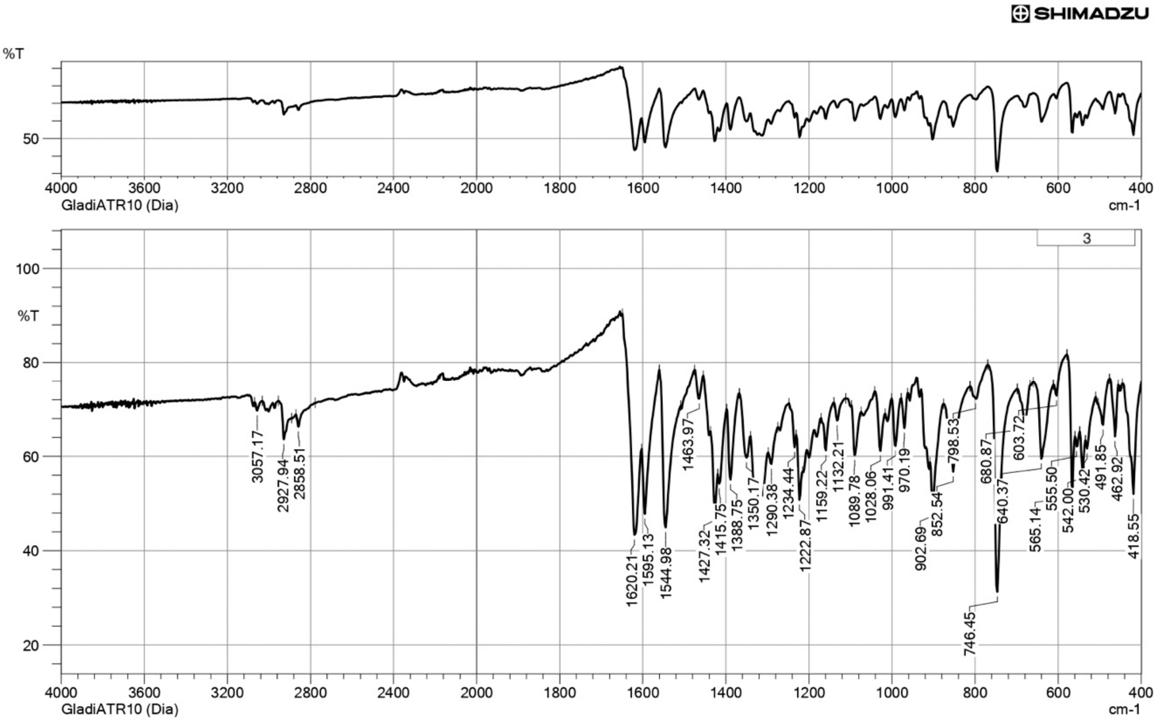

Compound 4

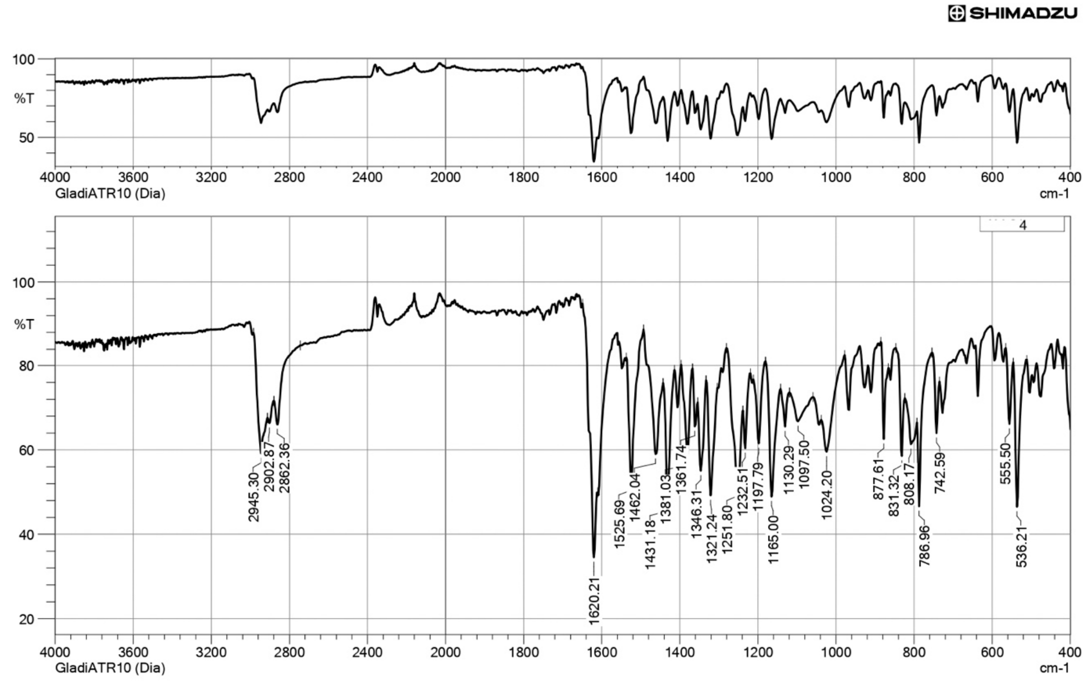

Compound 5

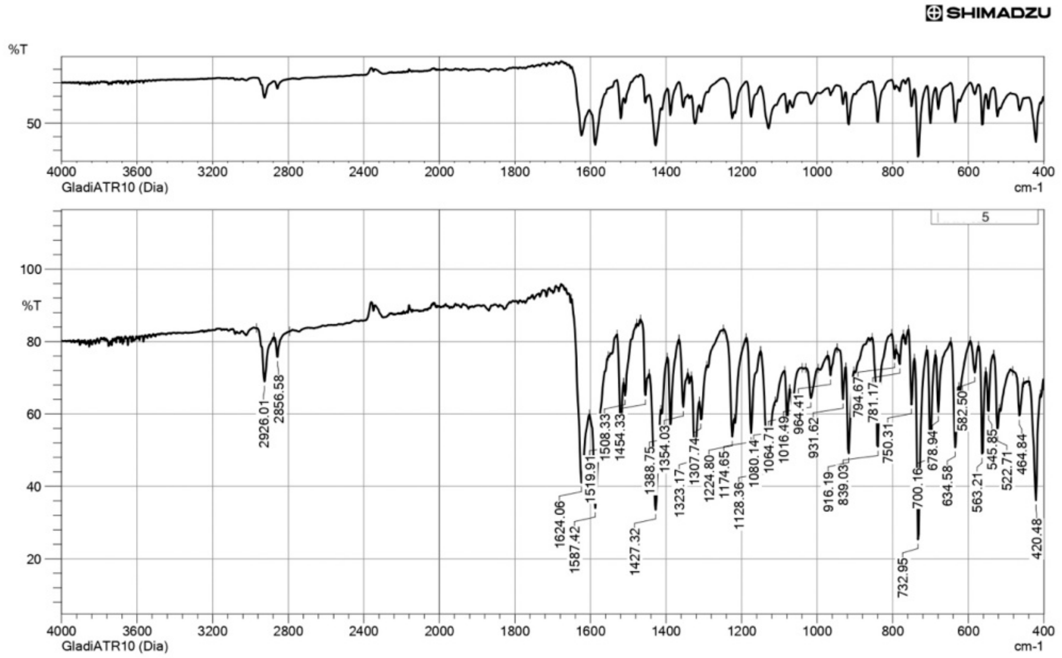

Compound 6

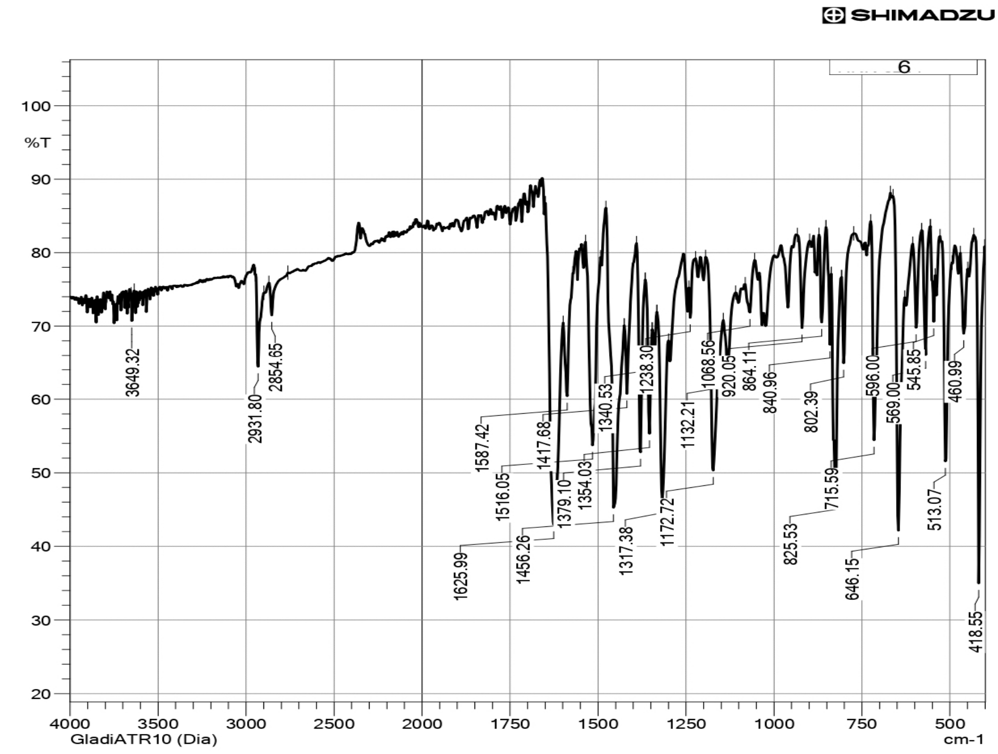

Compound 7

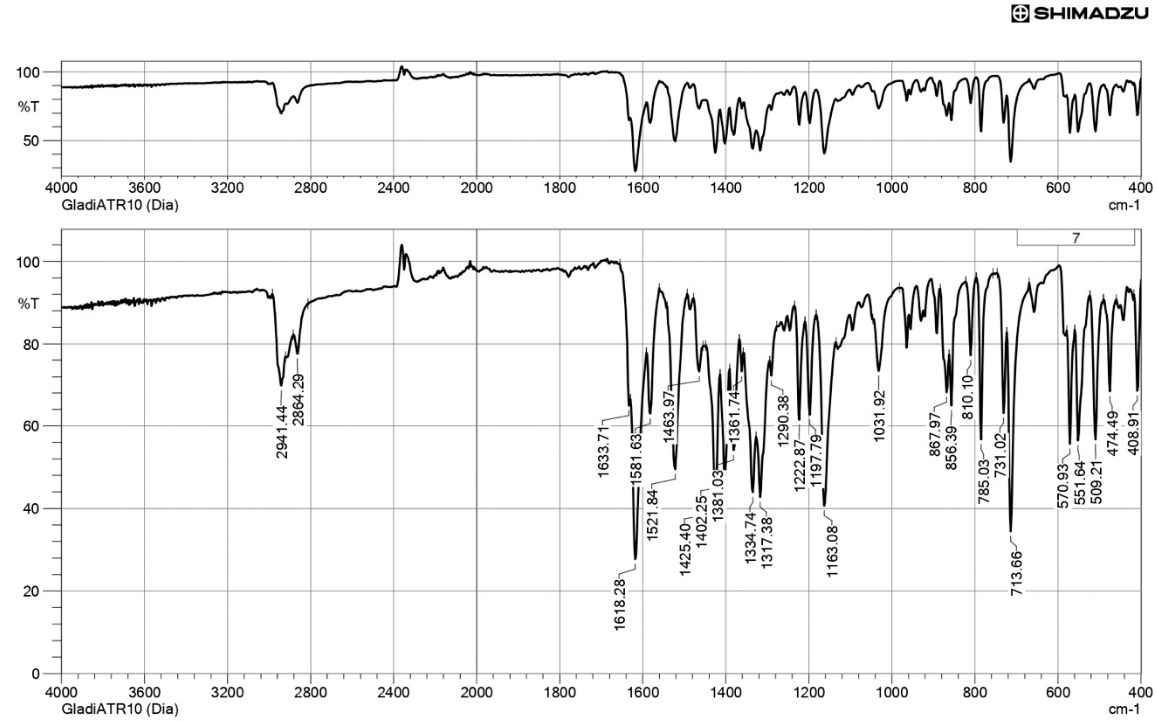

Compound 8

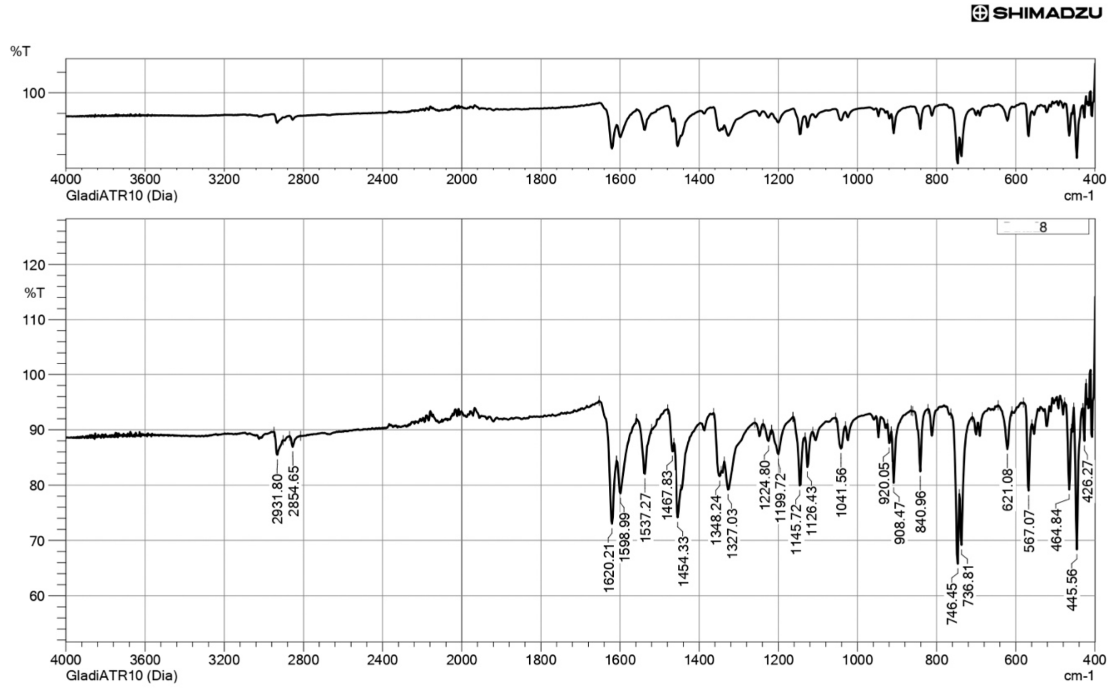

1

Compound 9

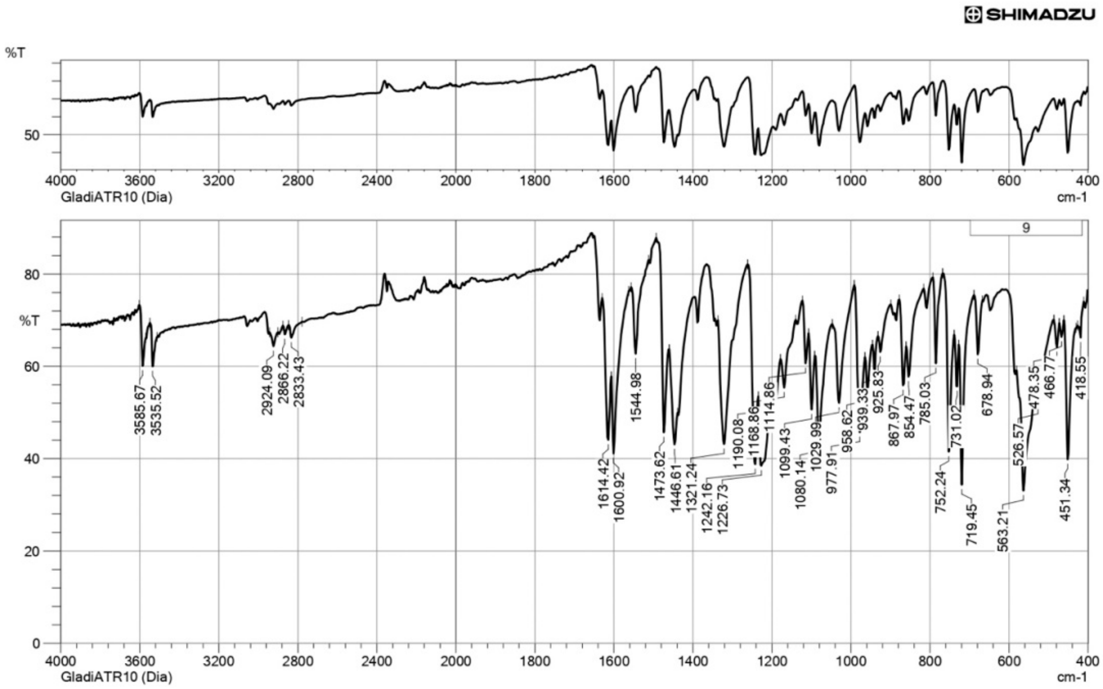

Compound 10

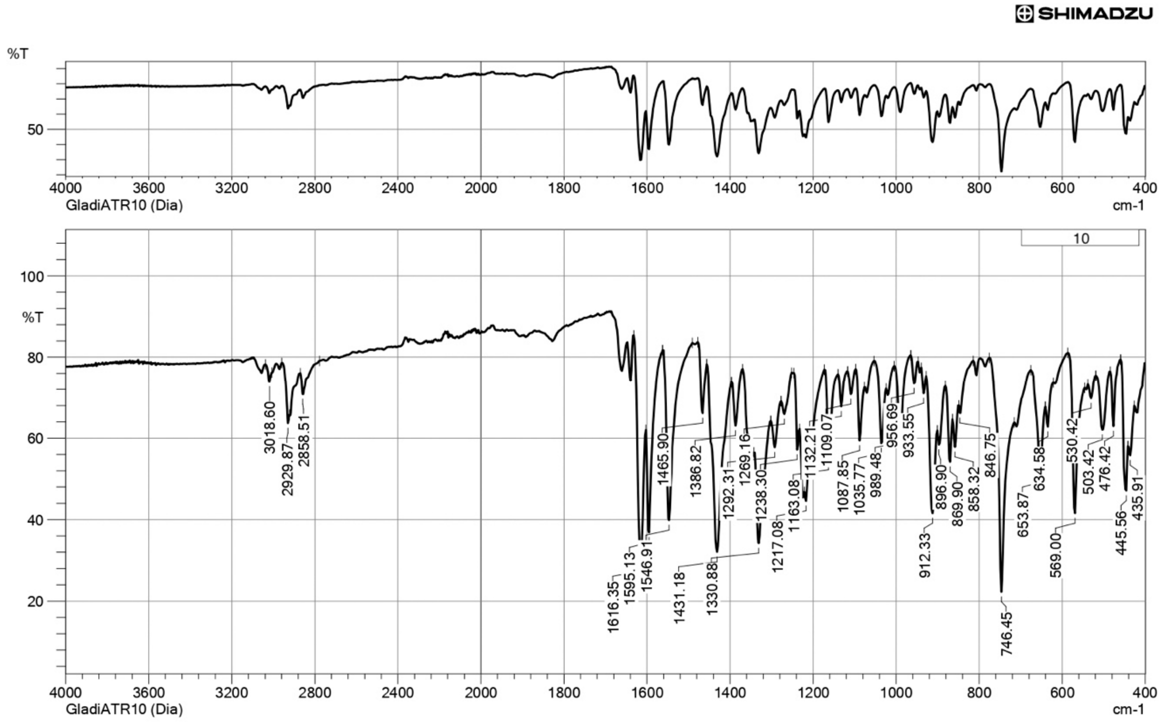

Compound 11

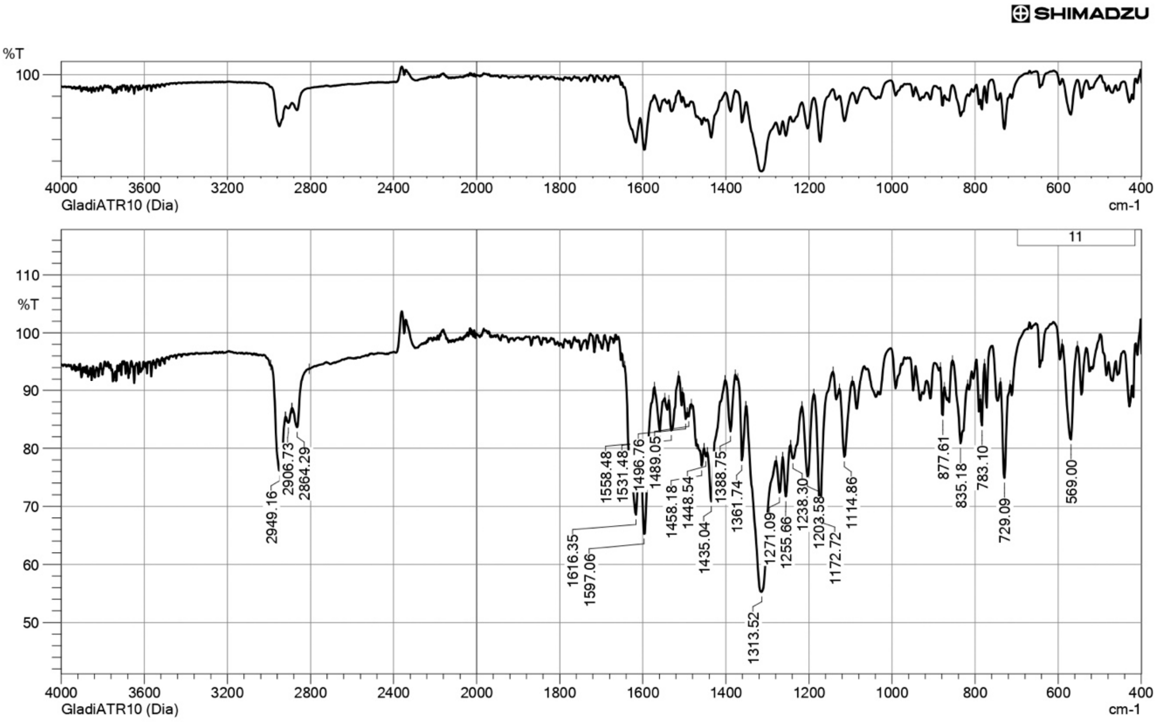

Compound 12

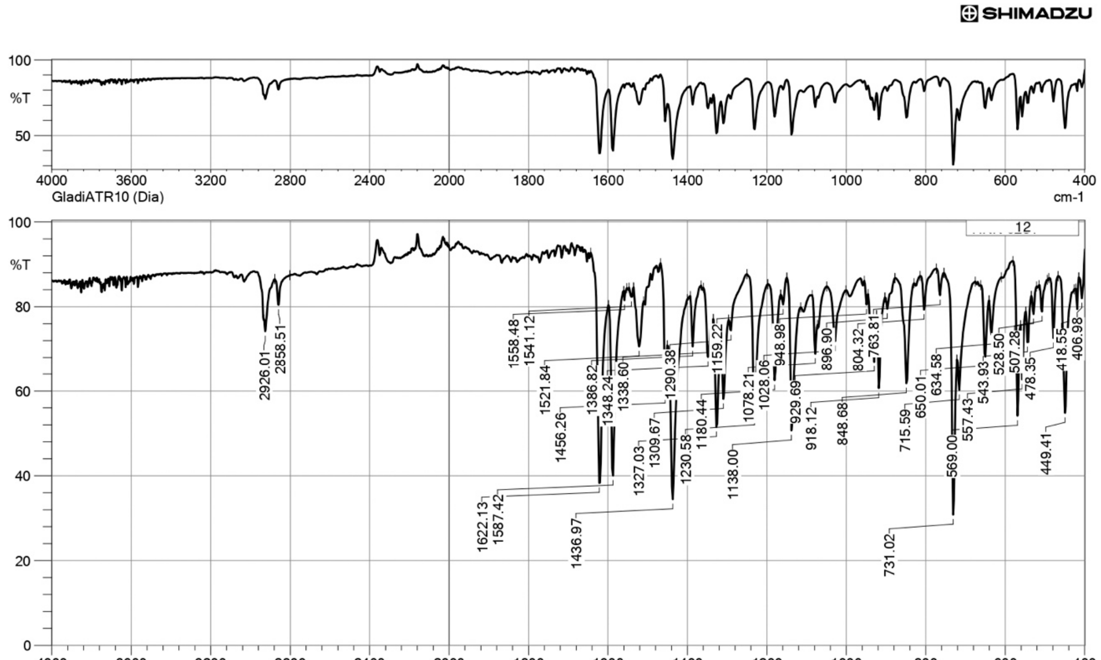

Compound 13

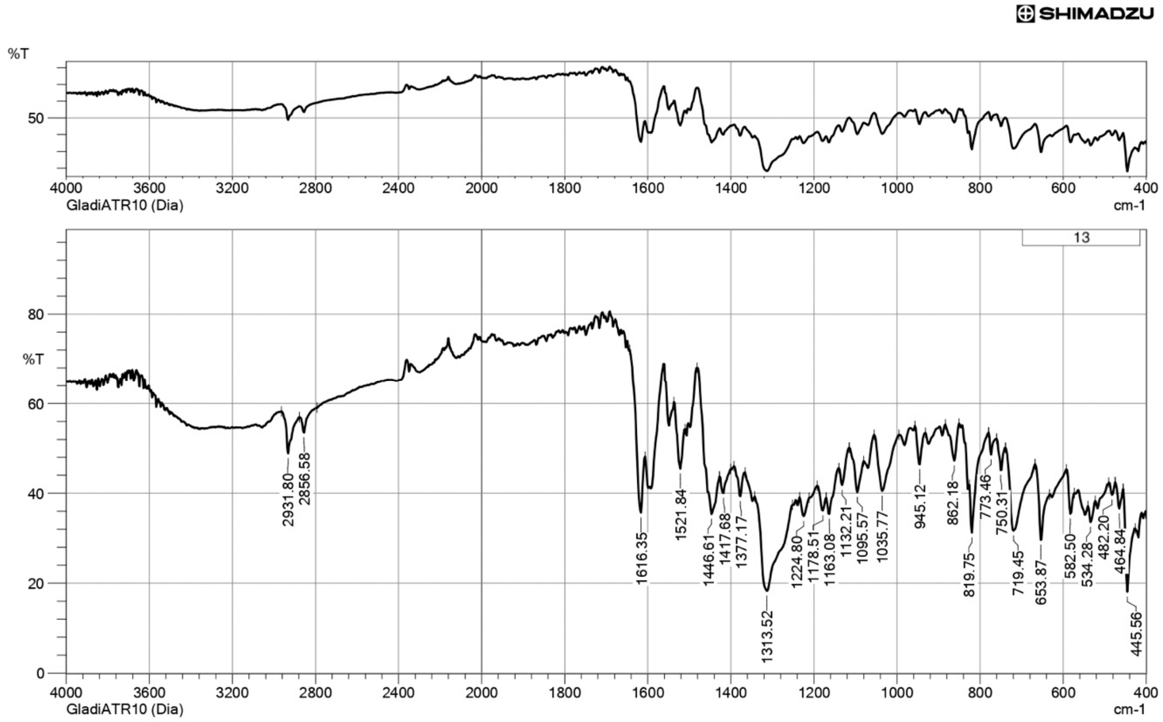

Compound 14

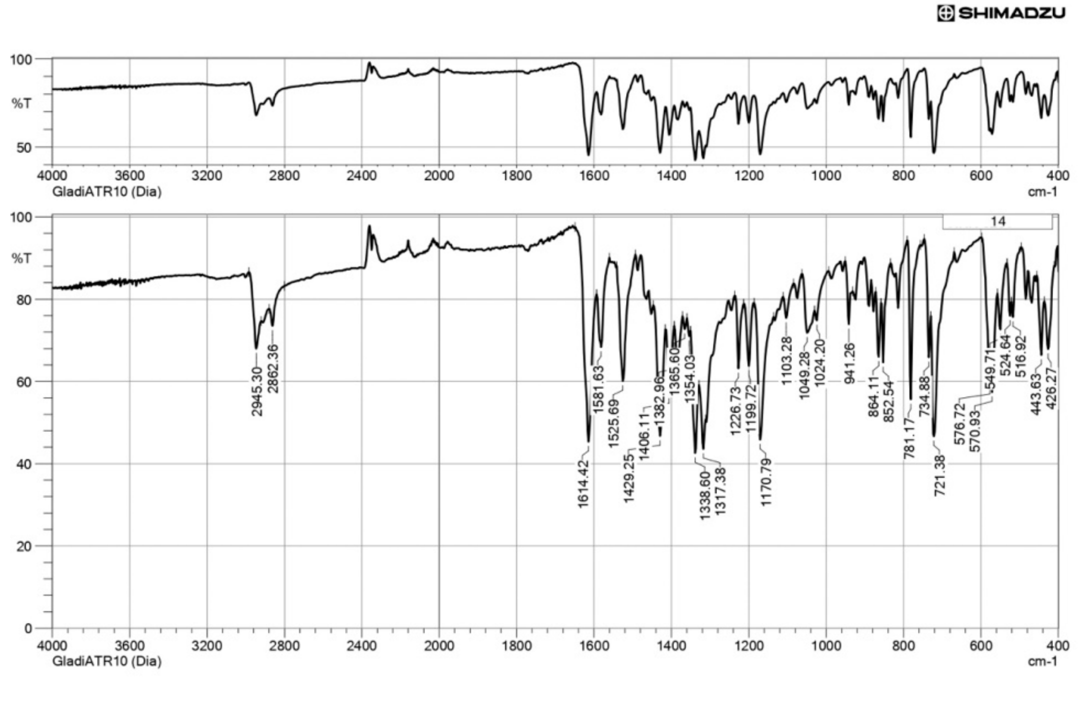

References

1.Tetrahedron Letters 40 (1999) 6105-6108

2.G.M. Sheldrick "Crystal structure refinement with SHELXL", Acta Cryst., (2015), C71, 3-8.

3.A. C. T. North, D. C. Phillips and F. S. Mathews, A Acta Cryst. (1968), A24, 351-359.
